# Using long ssDNA polynucleotides to amplify STRs *loci* in degraded DNA samples

**DOI:** 10.1371/journal.pone.0187190

**Published:** 2017-11-03

**Authors:** Martín E. Mautner, Agustín Pérez Santángelo, Rodrigo M. Corti Bielsa, Andrea Sala, Santiago Ginart, Daniel Corach

**Affiliations:** 1 Biodynamics SRL, Buenos Aires, Argentina; 2 Laboratorio de Neurociencia, Universidad Torcuato Di Tella, Buenos Aires, Argentina; 3 Department of Forensic Genetics and DNA Fingerprinting Service, School of Pharmacy and Biochemistry, Universidad de Buenos Aires, Buenos Aires, Argentina; University of Helsinki, FINLAND

## Abstract

Obtaining informative short tandem repeat (STR) profiles from degraded DNA samples is a challenging task usually undermined by *locus* or allele dropouts and peak-high imbalances observed in capillary electrophoresis (CE) electropherograms, especially for those markers with large amplicon sizes. We hereby show that the current STR assays may be greatly improved for the detection of genetic markers in degraded DNA samples by using long single stranded DNA polynucleotides (ssDNA polynucleotides) as surrogates for PCR primers. These long primers allow a closer annealing to the repeat sequences, thereby reducing the length of the template required for the amplification in fragmented DNA samples, while at the same time rendering amplicons of larger sizes suitable for multiplex assays. We also demonstrate that the annealing of long ssDNA polynucleotides does not need to be fully complementary in the 5’ region of the primers, thus allowing for the design of practically any long primer sequence for developing new multiplex assays. Furthermore, genotyping of intact DNA samples could also benefit from utilizing long primers since their close annealing to the target STR sequences may overcome wrong profiling generated by insertions/deletions present between the STR region and the annealing site of the primers. Additionally, long ssDNA polynucleotides might be utilized in multiplex PCR assays for other types of degraded or fragmented DNA, *e*.*g*. circulating, cell-free DNA (ccfDNA).

## Introduction

The amplification of standardized short tandem repeats (STRs) *loci* [1–6] by PCR [7] from different types of DNA samples is a reliable and widespread method for obtaining individual genetic profiles for archaeological, forensic, paternity and DNA database applications [8]. Available commercial multiplex kits [9–11] are routinely used worldwide to investigate human remains of archaeological interest and to help law enforcement agencies and the judicial system in testing forensically relevant evidentiary material [12, 13].

It is noteworthy that none of the current commercially available capillary electrophoresis (CE) kits is well suited to fully amplifying the degraded DNA samples usually found in most archaeological, aged and forensic samples. This downside is—to some extent—not inherent to the PCR technique, but arises from the need to amplify long off-target sequences in order to obtain amplicons of increasing and non-overlapping sizes suitable for CE discrimination. Existing multiplex kits need to amplify amplicons of increasing sizes to fit as many markers as possible in the CE range from 100 to 450nt without creating any overlaps. To do so, extra sequences (off-target), i.e. beyond the repeats, need to be amplified too—but longer amplicons cannot be amplified from degraded DNA

This approach creates many of the *locus* or allele dropouts and peak high imbalances that are observed in CE electropherograms, especially for those markers having large amplicon sizes.

The use of mini-STRs for reducing the extension of the DNA amplification, and thereby improving the PCR yield in degraded DNA samples, has been described by Whitaker JP *et al*. [14] and by Butler JM *et al*. [15]. Hill *et al* have characterized 26 miniSTR with 25 non-CODIS *loci* [16] and described a 26plex autosomal STR assay aimed to amplify degraded DNA samples [17]. Aghanoori MR *et al* have used mini-STRs for fetal sex determination from fragmented, circulating cell-free DNA (ccfDNA) in maternal plasma [18]. The mini-STRs primers target sequences adjacent to the repeats to reduce the extent of intact DNA that is necessary to amplify the desired STR. However, this method gives always short DNA amplicons of approximately 100bp, thereby limiting the number of non-overlapping mini-STRs that can be used in multiplex assays.

Mobility-modifiers [19] were introduced by Applied Biosystems (Foster City, USA) in 2001 to provide larger DNA amplicons without the need to actually amplify the DNA equivalent to its length, thereby reducing the extent of the amplification. By incorporating hexaethylenoxide (HEO) units into a primer the mobility-modifying polymers can provide larger DNA amplicons. Since the mobility-modifying polymer is not amplifiable, to detect the increased amplicon size the polymer must be linked to a fluorescent dye in order to detect the DNA strand where the mobility modifier is incorporated. This limits the length of this polymer to only short sizes to avoid any overlapping with the labelled PCR products present in the electropherogram. Mobility modifiers are currently used for some genetic markers, e.g. CSF1PO, in the commercial kit Identifiler^™^ (Thermo Fisher Scientific Inc., Waltham, USA).

**Fig 1:**
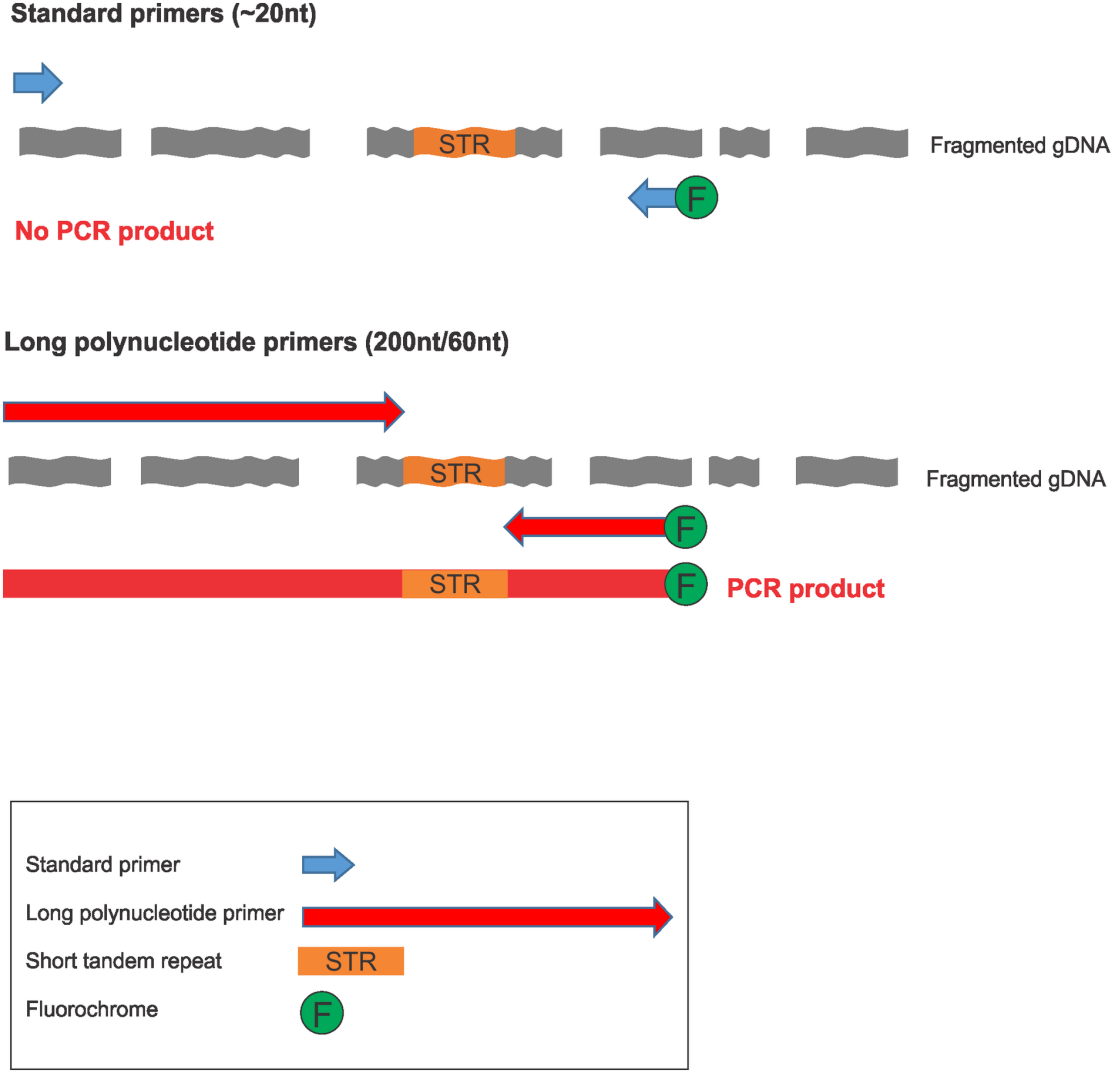
PCR layout for standard vs. long primers on fragmented gDNA.

We herein propose the use of long primers [20, 21] to circumvent the need to extend large stretches of DNA, while at the same time obtaining amplicons suitable for size discrimination (Fig 1).

The long primers can be designed to anneal close to the target repeat regions, making them ideal to amplify fragmented DNA in a similar fashion to the mini-STRs. Differently from the mini-STR approach, the use of long primers provides amplicon sizes of variable lengths that are well suited for CE discrimination in multiplex assays.

We hereby show that the use of long ssDNA polynucleotides as primers on degraded DNA samples can discriminate genotypes otherwise missed by the currently used methods.

## Materials and methods

### Primer sequences

**Fig 2:**
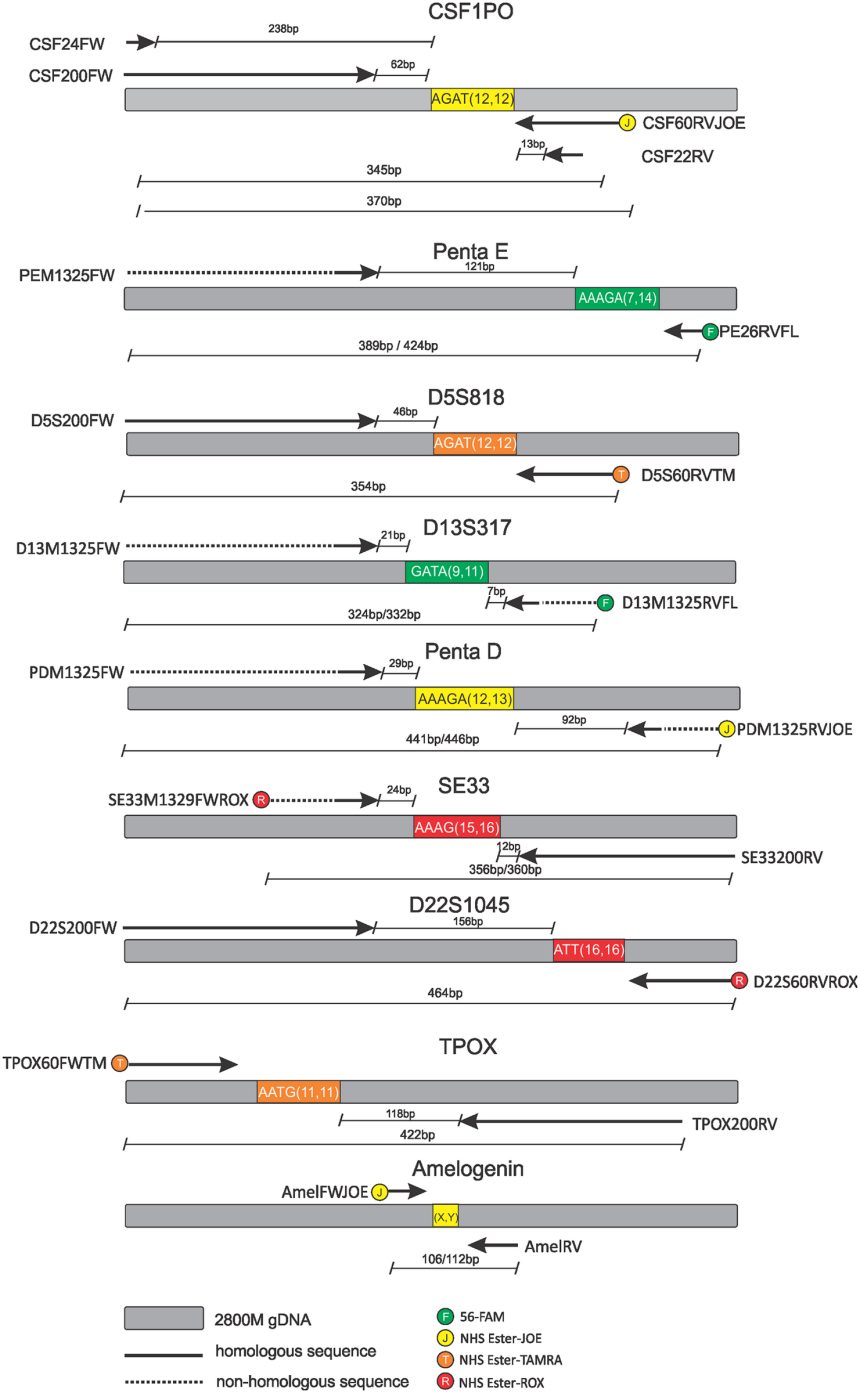
Primer design showing the size of the amplification products for 2800M gDNA.

The PCR primer sequences (Fig 2 and Table 1) were designed based on information provided by the STRbase website [12] and the published genome sequence from the GenBank (Accession numbers X14720 (CSF1PO), AC027004 (Penta E), AC008512 (D5S818), G09017(D13S317), AP001752 (Penta D), V00481 (SE33), AL022314 (D22S1045), M68651 (TPOX) and NG008011 (amelogenin). All single-stranded oligonucleotides and polynucleotides were synthesized by Integrated DNA Technologies, IDT (Coralville, USA). Fluorescently labeled primers were purified by HPLC. Long ssDNA polynucleotides of 200 nucleotides were purified by PAGE. All other primers were desalted.

**Table 1:** Names and sequences of primers.

Primer name	Direction	Label	Sequence^a^	Target *locus*
CSF24FW	Forward	-	ccggaggtaaaggtgtcttaaagt	CSF1PO
CSF22RV	Reverse	-	atttcctgtgtcagaccctgtt	CSF1PO
CSF200FW	Forward	-	cggaggtaaaggtgtcttaaagtgagaaagaataactgcatcttaacctattgggaggtcattgtaaagaggagagtgatggggtcagattgtacagaggaggcacttcgtggtggtcaggagcacacactccagggcagtgttccaacctgagtctgccaaggactagcaggttgctaaccaccctgtgtctcagttt	CSF1PO
CSF60RVJOE	Reverse	NHS Ester-JOE	JOE/atctcctggtgcacacttggacagcatttcctgtgtcagaccctgttctaagtacttcct	CSF1PO
PEM1325FW	Forward	**-**	**ccatttgcgaaatgtatctaatggtcaaactaaatctactcgttcgcagaattgggaatcaactgttacatggaatgaaacttccagacaccgtactttagttgcatatttaaaacatgttgagctacagcaccagattcagcaattaagctctaagccatccgcaaaaatgacc**gctgggtgtggtggtaggcacctgt	Penta E
PE26RVFL	Reverse	56-FAM	6FAM/tgggttattaattgagaaaactccttacaattt	Penta E
D5S200FW	Forward	-	ggcttaccccctcattttgaaaatacatgggagaaaataatacatagccacatttgtaattttctaattcaaaggagtatataattatgtaataattttaaaattaaatactgagacatgcatatgcttttaaagcttctaattaaagtggtgtcccagataatctgtactaataaaagtatattttaatagcaagtatg	D5S818
D5S60RVTM	Reverse	NHS Ester-TAMRA	TAMRA/ccaatcatagccacagtttacaacatttgtatctttatctgtatccttatttatacctct	D5S818
D13M1325FW	Forward	**-**	**acctgatttttgatttatggtcattctcgttttctgaactgtttaaagcatttgagggggattcaatgaatatttatgacgattccgcagtattggacgctatccagtctaaacattttactattaccccctctggcaaaacttcttttgcaaaagcctctcgctattttggttt**taggcagcccaaaaagacagacaga	D13S317
D13M1325RVFL	Reverse	56-FAM	6FAM/**ctggtaaacgagggttatgatagtgttgctcttac**catctaacgcctatctgtatttaca	D13S317
PDM1325FW	Forward	**-**	**ttgctcttactatgcctcgtaattccttttggcgttatgtatctgcattagttgaatgtggtattcctaaatctcaactgatgaatctttctacctgtaataatgttgttccgttagttcgttttattaacgtagatttttcttaaaatcgcataaggtaattcacaatgattaa**tacactccagcctaggtgacagagc	Penta D
PDM1325RVJOE	Reverse	NHS Ester-JOE	JOE/**tcttcccaacgtcctgactggtataatgagccagt**taataggtcatgattttgtgatatc	Penta D
SE33M1329FWROX	Forward	NHS Ester-ROX	ROX/**aattgatgccaccttttcagctcgcgcccca**ggaaggaaggaagaaaaagaaagaaaaag	SE33
SE33200RV	Reverse	-	ccttgcgcatgctggtgcagttgtcgacgacgacgagcgcggtgatagcatcatccatggtgagctggcggcgggtgcggacgcaaggcgcagcggcaaggacaaggttctgtgctcgctgggctgacgcggtctccgcggtgtaaggaggtttatatatatttctacaacatctcccctaccgctatagtaacttgctc	SE33
D22S200FW	Forward	-	tccccctacagggtgactgcatctccgagtcctggcttgtcatgcctgacagagggctgccgagtgagcagcttaaggcatcctgccactgtgcagctgccaaccctacagcccggcagccctgcgggaggaagctctagtgcaggcctcttaggatctggggtccaggatgctgatttcagggccgggaccttgggcac	D22S1045
D22S60RVROX	Reverse	NHS Ester-ROX	ROX/agagtgcccggcacagtgtgagtgatcacgcgaatgtatgattggcaatatttttataac	D22S1045
TPOX60FWTM	Forward	NHS Ester-TAMRA	TAMRA/gatcactagcacccagaaccgtcgactggcacagaacaggcacttagggaaccctcactg	TPOX
TPOX200RV	Reverse	-	actgctgtgtcgtgactccccctgtgtgggaccctcgtggcggctcttacactgctgtgtcgtgactcctcctgtgtgctcacaccaagcactctcgtgtttgcgaccccaacgctcaaacgtgactcctcctgtactgtcctgggcgctcaggggaggaactgggaaccacacaggttaattaagagattcatccaaaa	TPOX
AmelFWJOE	Forward	NHS Ester-JOE	JOE/ccctgggctctgtaaagaa	Amelogenin
AmelRV	Reverse	-	atcagagcttaaactgggaagctg	Amelogenin

^a^Non-homologous, M13 phage sequences are displayed in bold.

### Controlled degradation of genomic DNA

Human genomic DNA (gDNA) (2800M, Promega, Madison, USA) at 100pg/μl was incubated for 0, 30, 45, 60, 75 and 90 minutes at 95°C in 50μL of nuclease-free water and immediately chilled [22].

### Forensic DNA samples

DNA samples were obtained from decomposed corpse material from actual forensic cases (Table 2). Two sets of degraded human samples were investigated. The first set is a collection of DNA samples obtained from decomposed and fragmented corpse tissues emerging from a mass fatality that occurred in 1994. DNA had been extracted 23 years ago using a proteinase K/SDS (Sodium Dodecyl Sulphate) incubation, followed by organic solvent extraction [23], and stored at -20°C. Sample selection was based on the degree of decay of the original human fragments at the moment of collection. The second set consisted of three samples of fetal tissue remains, formalin-fixed and embedded in paraffin blocks, extracted with Maxwell 16 Casework Pro (Promega) [24]. All samples were quantified with Quantifluor^®^ dsDNA System (Promega) [25] or Plexor^®^ HY (Promega) [26] and analyzed with PowerPlex^®^ Fusion (Promega) or PowerPlex^®^ Fusion 6C (Promega) following the manufacturer directions [27, 28], except that the final reaction volume was 12.5μL. Sample collection, processing and handling were approved by the Ethical Committee of the School of Pharmacy and Biochemistry, Buenos Aires University under Exp.711987/2008.

**Table 2:** Description of the forensic samples.

Sample ID#	Source	Tissue type	Time elapsed from event to sample collection
39	Mass disaster victim (1994)	Muscle, bone, skin	7 d
60	Mass disaster victim (1994)	Lung	15 d
62	Mass disaster victim (1994)	Muscle, bone, skin	15 d
63	Mass disaster victim (1994)	Skin	15 d
66	Mass disaster victim (1994)	Skin, muscle	15 d
68	Mass disaster victim (1994)	Liver	15 d
69	Mass disaster victim (1994)	Muscle	15 d
71	Mass disaster victim (1994)	Trachea, lung	15 d
A2275	Fetal remains (2012)	Paraffin embeded tissue	5 y
A2624	Fetal remains (2015)	Paraffin embeded tissue	2 y
A2901	Fetal remains (2014)	Paraffin embeded tissue	3 y

### PowerPlex^®^ Fusion 6C amplification of degraded DNA

Control samples of 2800M gDNA (5μL, 100pg/μL) that had previously been heat-degraded for different times were amplified with PowerPlex^®^ Fusion 6C (Promega). The reactions were performed as specified by the manufacturer, except that the final reaction volume was 12.5μL. The PCR reactions were carried out in an ABI 9700 thermal cycler (Applied Biosystems) for the recommended 29 cycles or extended to 35 cycles.

### Quantitative PCR

Quantitative PCR (qPCR) reactions were performed in a Rotor-Gene RG-6000 cycler (Corbett, Sydney, Australia) in a reaction volume of 20μL containing 1X qPCR GoTaq^®^ qPCR Master Mix (Promega), 800pg of heat-treated 2800M gDNA and 500nM of each primer for pairs CSF24FW+CSF22RV or CSF200FW+CSF22RV (IDT). Cycling conditions included an initial denaturation step of 2 min at 94°C, followed by 40 cycles of 10 s at 94°C, 60 s at 59°C and 45 s at 72°C plus a final incubation of 10 min at 60°C. Dye-fluorescence was detected at 470nm/510nm (excitation/emission) wavelengths. Cycle threshold (Ct) values were determined manually based on the logarithmic graph.

### Long ssDNA polynucleotide triplex PCR mix

A long ssDNA polynucleotide triplex PCR mixture was prepared with 1X Colorless GoTaq^®^ reaction buffer (containing 1.5mM MgCl_2_) (Promega), 200uM of each dNTP (Promega, USA), 1U GoTaq^®^ Hot Start DNA Polymerase (Promega), 500nM of each of the following primers (IDT): PEM1325FW, PE26RVFL, D5S200FW, D5S60RVTM and 250nM of CSF200FW and CSF60RVJOE.

### Long ssDNA polynucleotide nonaplex PCR mix

A long ssDNA polynucleotide multiplex PCR mixture was prepared with 1X Colorless GoTaq^®^ reaction buffer (containing 1.5mM MgCl_2_) (Promega), 0.625 mM of additionally supplemented MgCl_2_ (Promega), 200uM of each dNTP (Promega), 2.5 units of GoTaq^®^ Hot Start DNA Polymerase (Promega) a set of primers (IDT) at the following concentrations: 250nM for PEM1325FW, PE26RVFL, D5S200FW, D5S60RVTM, SE33M1329FWROX, SE33200RV, TPOX60FWTM, TPOX200RV and D22S200FW, D22S60RVROX; 125nM for CSF200FW, CSF60RVJOE, D13M1325FW, D13M1325RVFL, PDM1325FW and PDM1325RVJOE and 62.5nM for AmelFWJOE and AmelRV.

### End-point PCR assays

Triplex and nonaplex PCR amplifications were performed in a MultiGene^™^ Gradient thermal cycler (Labnet, New Jersey, USA) in a 20μL reaction volume containing the long ssDNA polynucleotide triplex or nonaplex mixtures. Cycling conditions encompassed an initial denaturation step of 2 min at 94°C, followed by 35 cycles of 10 s at 94°C, 60 s at 59°C and 45 s at 72°C and a final incubation of 30 min at 60°C. The triplex assay contained 500pg of heat-degraded gDNA standard 2800M and 1ng of forensic DNA samples. The nonaplex assays contained 500pg or 1ng of gDNA standard 2800M and 1 or 10ng of forensic DNA samples.

### Gel electrophoresis

All amplification products were treated with Blue/Orange Loading Dye, 6X (Promega). Aliquots of 5 uL were loaded on a 2% agarose gel (Hispangar, Burgos, Spain) and run at 110V constant voltage in TBE buffer, 1X (Promega) with 5μg/mL ethidium bromide (Promega). The size standard was 100bp DNA ladder (Promega), where the band of 500bp has triple intensity. The DNA products were visualized at 302nm with a UV trans-illuminator (Maestrogen, Hsinchu City, Taiwan).

### Capillary electrophoresis

Sample dilutions of the PCR products were run in an ABI 3500 sequencer (Applied Biosystems) with a 50cm-capillary array and POP7 polymer (Applied Biosystems) using the following parameters: oven temperature, 60°C; pre-run voltage, 15 KV during 180 s; injection voltage, 1.3 KV; injection time, 5 s. Analytical separation was performed at 17.5 KV, run for 25 min and the 5-dye set (Promega) was applied. An aliquot (0.5 or 1 μL) of PCR product was added to a 9.5 μL cocktail (9.0 μL Hi-Di™ Formamide and 0.5 μL WEN Internal Lane Standard (ILS, Promega)). Results were analyzed using GeneMapper^®^ ID-X 1.2 expert software (Applied Biosystems).

### *In silico* primer analysis

*In-silico* analyses of the primer interactions were performed with the AutoDimer software developed by Vallone *et al* [29] at the National Institute of Standards and Technology (NIST). All software parameters were set at default values except for the Total Strand Concentration (0.5uM). Thus, the minimum score for detection (threshold) was set at 7. The potential formation and stability of the primer-dimers are reported as the relevant interactions (hits), *i*.*e*. those involving primer sequences by means of complementarity score (base matches—mismatches), the longest uninterrupted base stretch and the predicted thermodynamic values (Tm and ΔG).

## Results

### Degraded DNA causes a dropout of the larger amplicons in STR profiles

We first performed a controlled degradation of gDNA by heating different samples of the 2800M genomic DNA standard at 95°C for increasing periods of time. We then amplified the resulting fragmented templates using the commercial kit PowerPlex^®^ Fusion 6C, which amplifies 27 markers in a single reaction, including the 20 expanded markers of the Combined DNA Index System (CODIS).

**Fig 3:**
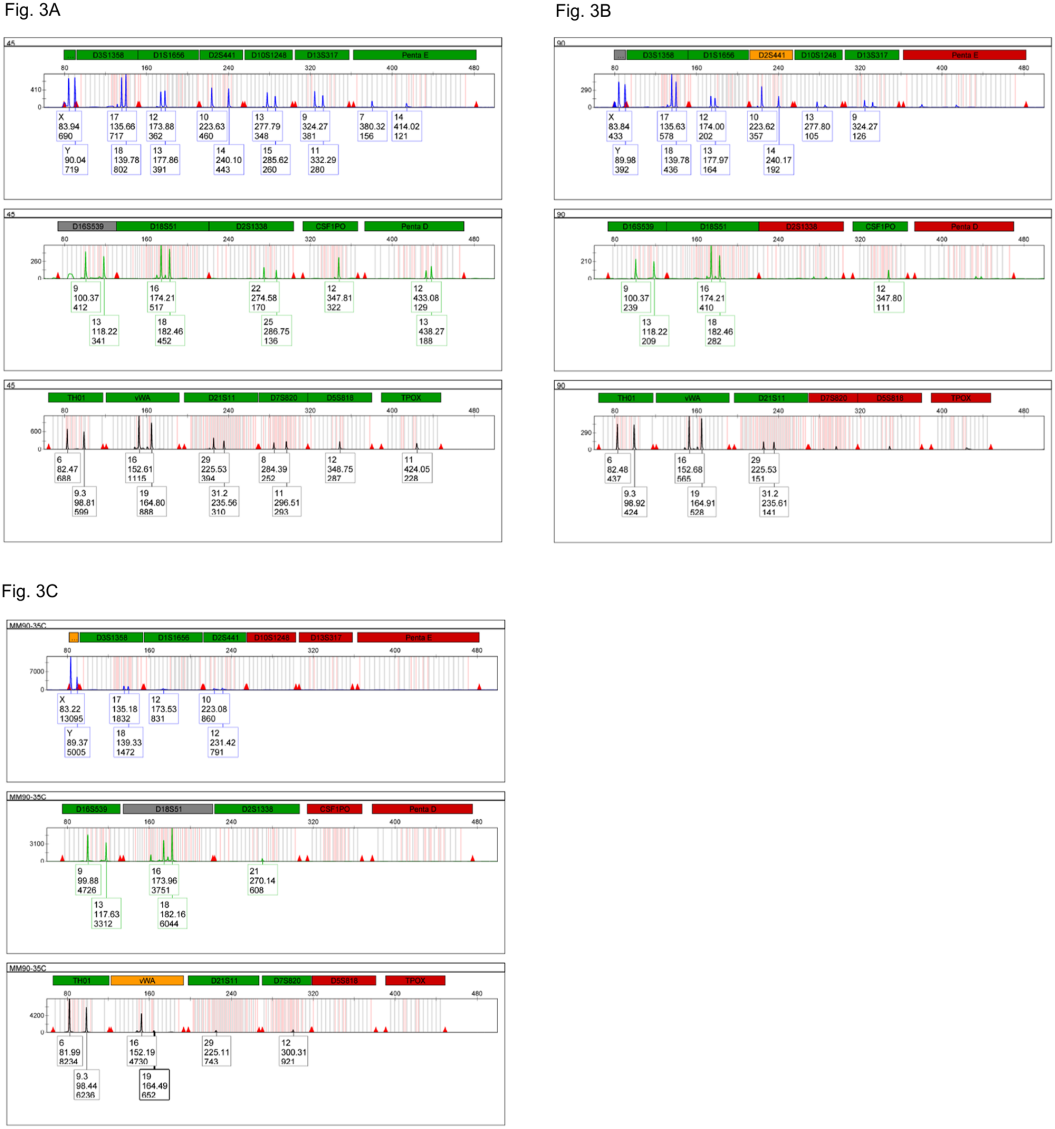
Electropherograms of 2800M gDNA heat-degraded for different times and amplified with PowerPlex^®^ Fusion 6C for 29 or 35 cycles. (A) gDNA heat-degraded for 45 min and amplified for 29 cycles (Fluorescein, JOE and TAMRA channels). (B) gDNA heat-degraded for 90 min and amplified for 29 cycles (Fluorescein, JOE and TAMRA channels). (C) gDNA heat-degraded for 90 min and amplified for 35 cycles (Fluorescein, JOE and TAMRA channels).

As expected, there is an increasing dropout for the large amplicons in the DNA templates heated for longer times (Fig 3). Since the PCR primer pairs in larger amplicons are far apart, longer segments of intact DNA are needed for a PCR amplification to occur. As the DNA becomes more fragmented the amplification yield for the larger markers decreases dramatically. This is clearly seen in the 45-minute preheated sample, where the larger STR markers show lower peak highs in the CE profiles (Fig 3A). Beyond the 60 minutes the large STR markers yield either very low peaks or allele dropouts (Fig 3B). Increasing the number of cycles from 29 to 35 did not revert the drop-out effect, but it generated extra noise and artifacts (*i*.*e*., drop-in events) (Fig 3C).

### Long primers performed better than standard primers in degraded DNA samples

To assess the advantage of using long primers for the amplification of large amplicons in degraded DNA samples, we compared the amplification performance of the CSF1PO STR marker using two different sets of forward primers that share the same 5’-end, but have different lengths of 24 nucleotides (CSF24FW) [30] and 200 nucleotides (CSF200FW). A common reverse primer (CSF22RV) [30] of 22 nucleotides was employed in both reactions. The resulting amplicon bears the same nucleotide sequence and length (345bp for 2800M gDNA) in both cases, but the 3’ end of the long primer anneals much closer to the repeat sequences than the standard primer (Fig 2). We performed qPCR reactions adding either pair of primers to DNA pretreated at 95°C for different periods of time.

**Fig 4:**
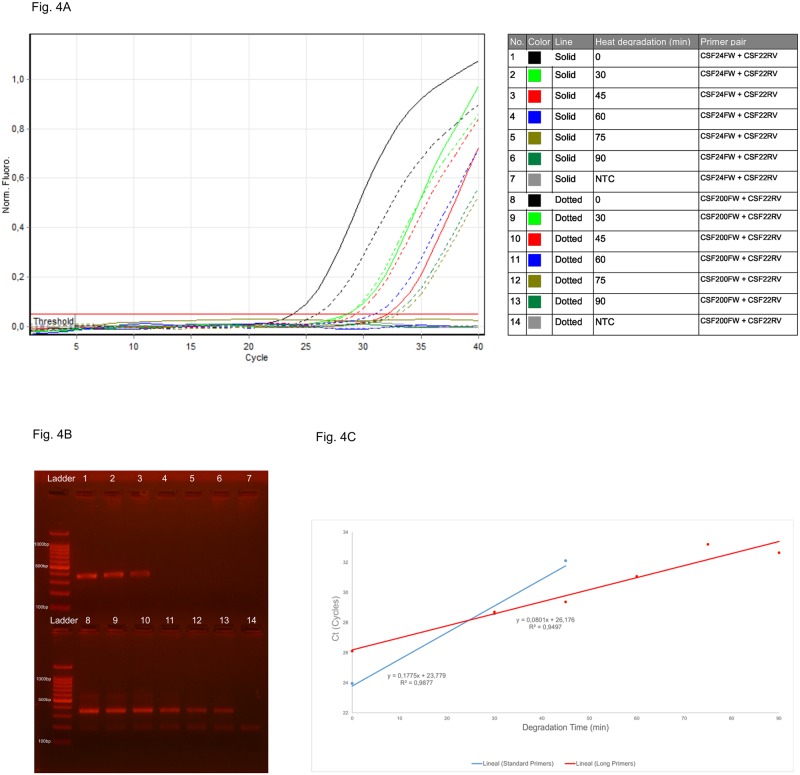
qPCR amplification with primer pairs CSF24FW+CSF22RV *vs*. CSF200FW+CSF22RV on degraded 2800M gDNA. (A) qPCR amplification curve. (B) 2% agarose gel electrophoresis of qPCR amplification products. Upper panel: Ladder (100bp marker, Promega, USA), samples 1–7; Lower panel: Ladder (100bp marker, Promega, USA), samples 8–14. (C) Regression curve of Ct values.

While for intact DNA (time 0) the shorter, standard 24-mer primer (CSF24FW) gives a lower Ct than the 200-mer long primer (CSF200FW), for 30-minute treated DNA both primers reach approximately the same Ct. From that time point onwards, the long primer pair amplifies with greater efficiency (Fig 4B), giving a lower Ct—and yielding a PCR product even at the 90-minute time point (Table 3).

**Table 3:** Ct values from qPCR amplification with primer pairs comparing CSF24FW+CSF22RV *vs*. CSF200FW+CSF22RV on degraded 2800M gDNA.

Heat degradation (min)	Sample No.	Ct values (Standard primer pair CSF24FW + CSF22RV)	Sample No.	Ct values (Long primer pair CSF200FW + CSF22RV)
0	1	23.95	8	26.10
30	2	28.59	9	28.70
45	3	32.11	10	29.37
60	4	-	11	31.07
75	5	-	12	33.20
90	6	-	13	32.64
NTC	7	-	14	-

On the other hand the standard primer pair does not amplify any detectable PCR product beyond the 45-minute pre-heating treatment, even after extending the qPCR to 40 cycles (Fig 4A). These Ct values show that for intact to moderately degraded DNA the standard primers perform better than the long primers, most likely because their small size facilitates their annealing. As the DNA template becomes more degraded, the Ct/degradation-time ratio reverses, since the shorter primers being far apart cannot amplify the increasingly fragmented DNA template, while the long primers by annealing closer to the target sequence can still extend the fragmented stretches of degraded DNA. The Ct values for the long primer pair become lower beyond the intersecting point estimated at 25 minutes (Fig 4C). The above results showed the advantage of using long primers over standard ones for obtaining amplicons of large sizes from degraded DNA.

### Amplification of controlled degraded DNA with a long ssDNA polynucleotide triplex assay (CSF1PO/PENTA E/D5S818)

**Fig 5:**
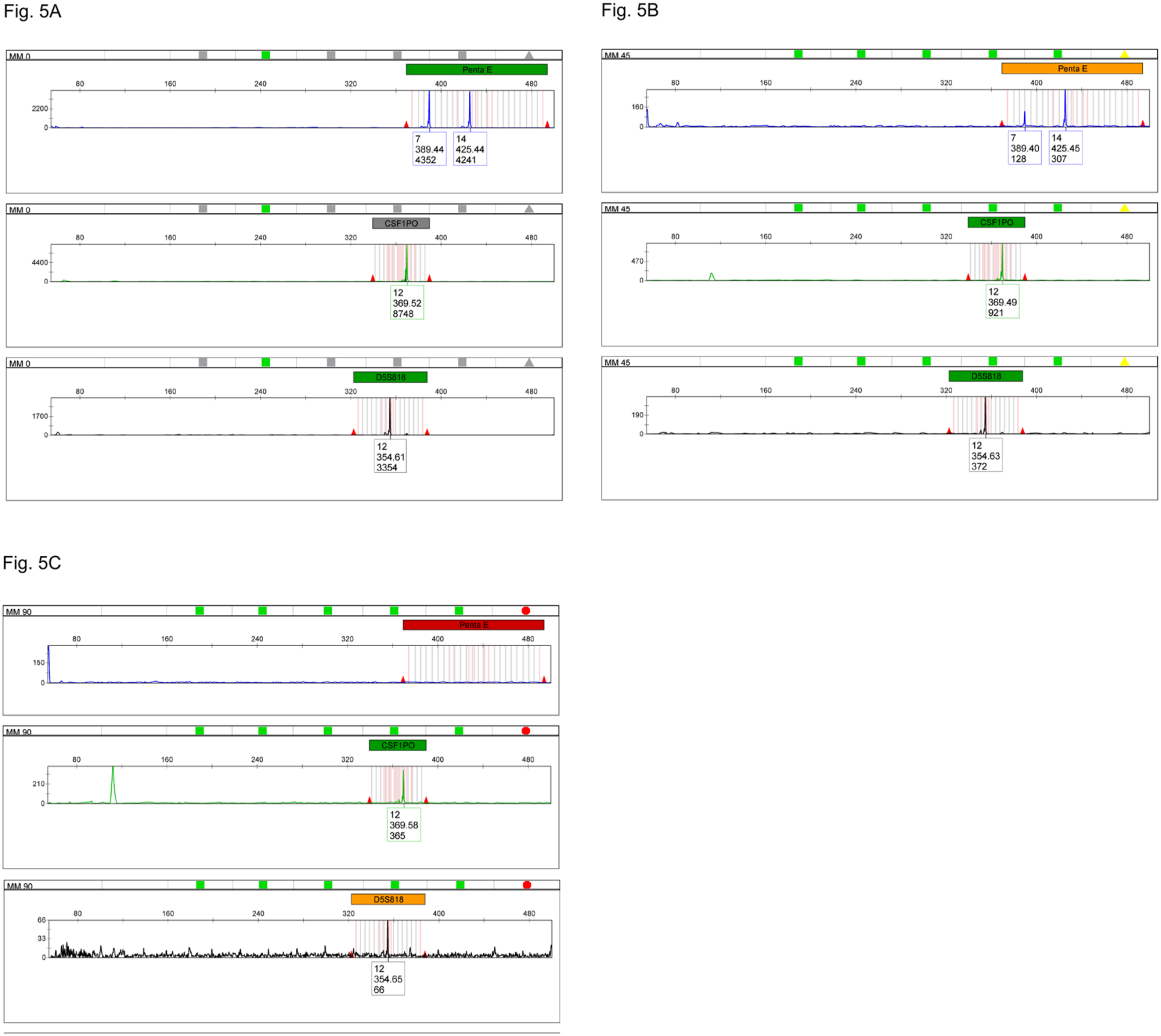
Electropherogram of 2800M gDNA heat degraded for different times and amplified with the CSF1PO/PENTA E/D5S818 long ssDNA polynucleotide triplex. (A) 2800M gDNA, non-heat degraded, 0 min positive control (Fluorescein, JOE and TAMRA channels). (B) 2800M gDNA heat degraded for 45 min (Fluorescein, JOE and TAMRA channels). (C) 2800M gDNA heat degraded for 90 min (Fluorescein, JOE and TAMRA channels).

Based on the previous results, a PCR mixture containing three long primer pairs was designed for the markers CSF1PO, PENTA E and D5S818 in order to test their performance as a polynucleotide triplex reaction on degraded DNA. Each pair of primers comprised of a long ssDNA polynucleotide of 200 nucleotides and a primer of up to 60 nucleotides labeled with following fluorescent dyes: FAM for Penta E, JOE for CSF1PO and TAMRA for D5S818. The long ssDNA polynucleotide mixture was used to amplify 2800M gDNA heat degraded for different times, as described in the Materials and Methods section. The long ssDNA polynucleotide triplex produced a complete STR profile at 45 minutes (Fig 5B) and some detectable profiles even at 90 minutes of preheating the sample (Fig 5C), although some stochastic effects may be observed beyond 60 minutes.

### Long primers do not need to be fully complementary to the target sequence for a successful amplification

In order to verify if long primers with partial non-complementary sequences may also successfully amplify specific DNA regions, the 200-nucleotide long primer for Penta E (PEM1325FW) was designed with only 25 nucleotides fully complementary to the Penta E sequence at the 3’-end, priming region and a non-complementary M13 phage tail of 175 nucleotides on the remaining 5’ region (Fig 2). PEM1325FW successfully amplified the Penta E region in the triplex PCR amplification of long primers, yielding products of the expected size (Fig 5A). The peak imbalance between the two Penta E alleles may be a stochastic effect due to the low amount of available template.

### Amplification of degraded forensic DNA samples with a long ssDNA polynucleotide triplex: CSF1PO/PENTA E/D5S818

The CSF1PO/PENTA E/D5S818 long ssDNA polynucleotide triplex was then used to amplify forensic DNA samples that had previously provided little or none information for these markers with the commercial kit PowerPlex^®^ Fusion. The long ssDNA polynucleotide mixture was able to provide additional allele information in most of the eleven DNA samples (Table 4) obtained from decomposed forensic material (Table 2).

**Table 4:** Assigned genotypes for CSF1PO, PENTA E and D5S818 markers in degraded cadaveric samples using PowerPlex^®^ Fusion and CSF1PO/PENTA E/D5S818 long ssDNA polynucleotide triplex.

	Penta E		CSF1PO		D5S818	
Sample ID#	PowerPlex^®^ Fusion	Polynucleotide triplex	PowerPlex^®^ Fusion	Polynucleotide triplex	PowerPlex^®^ Fusion	Polynucleotide triplex
2800M (control)	7,14	7,14	12,12	12,12	12,12	12,12
39	-	-	-	**12?,13?**	-	-
60	-	-	-	-	-	-
62	-	**15,17**^a^	11,11	**10**,11	-	**11,12**
63	-	-	-	-	-	-
66	-	-	12?,12?	12?,12?	*13*,*13*^b^	-
68	-	-	-	-	-	-
69	-	**7,16**	-	**10,13**	11,11	11,11
71	-	**7,13**	-	**11,11**	-	-
A2275	-	**11,14**	12,12	**10,**12	11,11	11,11
A2624	-	-	-	**12,12**	-	-
A2901	-	**5,5**	-	**10,11**	-	**10,12**

^**a**^Additional alleles obtained with the long primers are highlighted in bold.

^b^Allele pair obtained only with the standard primers are in italics.

(-) Absence of amplification.

(?) Indicates inconclusive typing.

**Fig 6:**
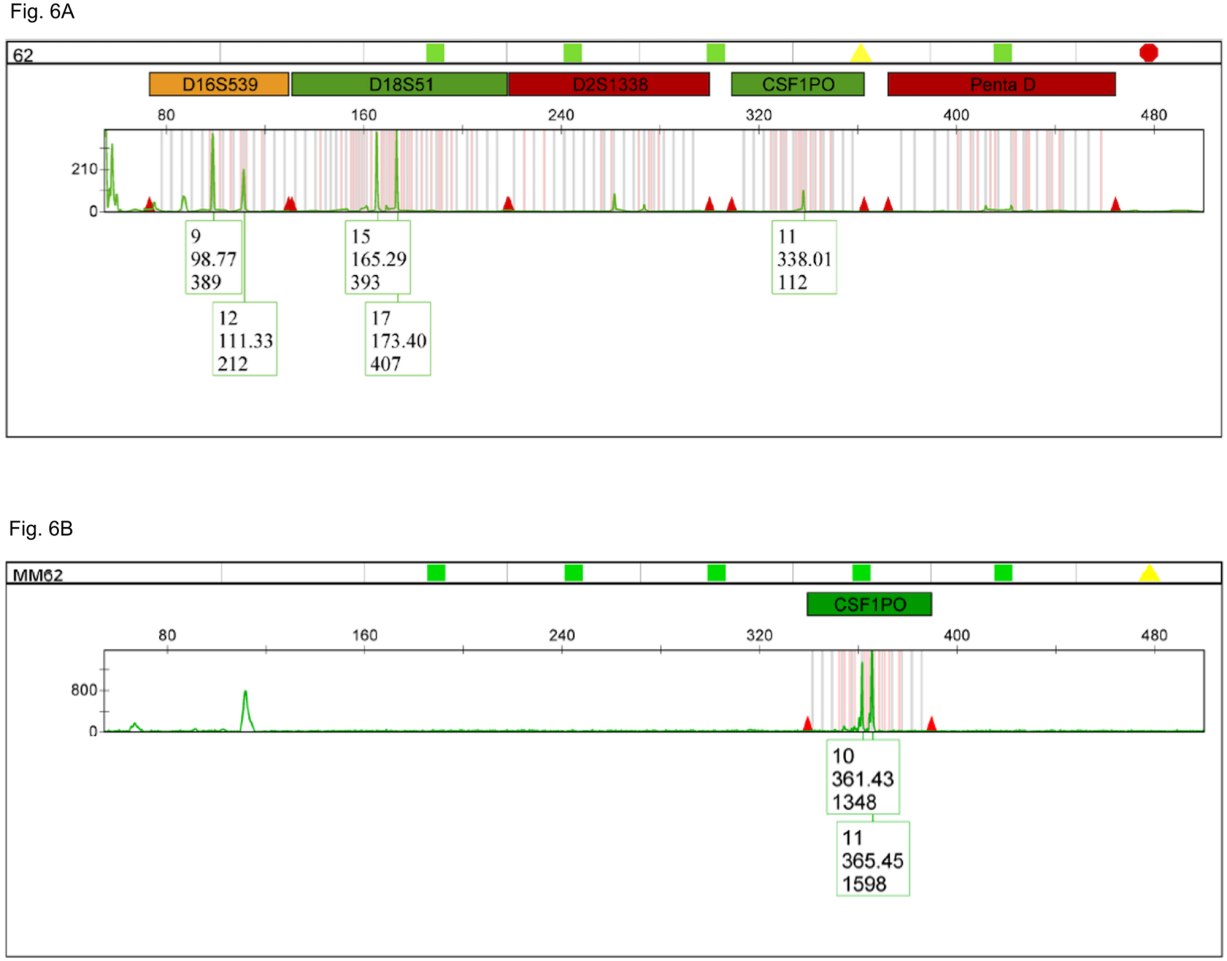
Electropherogram for cadaveric sample #62 amplified with PowerPlex^®^ Fusion vs. CSF1PO/PENTA E/D5S818 long ssDNA polynucleotide triplex. (A) Amplification results with PowerPlex^®^ Fusion showing a single allele (11) for the CSF1PO *locus* (JOE channel). (B) Amplification results with CSF1PO/PENTA E/D5S818 long primer triplex showing both alleles (10, 11) for the CSF1PO *locus* (JOE channel).

For instance, sample #62 which had been originally assigned as homozygote (11, 11) for locus CSF1PO showed to be, in fact, a heterozygote (10, 11) when amplified with the long ssDNA polynucleotide triplex mixture (Fig 6).

Except for one odd sample (#66), the long ssDNA polynucleotide triplex provided additional allele information, which had been missing from the previous results. Also, all the alleles recovered by the new method matched the genotypes of the samples that had an available reference profile (A2624: CSF1PO (12, 12); #71: Penta E (7, 13), CSF1PO (11, 11); #62: Penta E (15, 17), CSF1PO (10, 11), D5S818 (11, 12); #69: Penta E (7, 16), CSF1PO (10, 13)).

Hence, the long ssDNA polynucleotide triplex supplied more complete and accurate genotypes than the commercial assay at the standard concentration of 1ng of DNA sample.

### Amplification of degraded forensic DNA samples with a long ssDNA polynucleotide nonaplex: CSF1PO/Penta E/D5S818/ D13S317/Penta D/TPOX/SE33/D22S1045/Amelogenin

**Fig 7:**
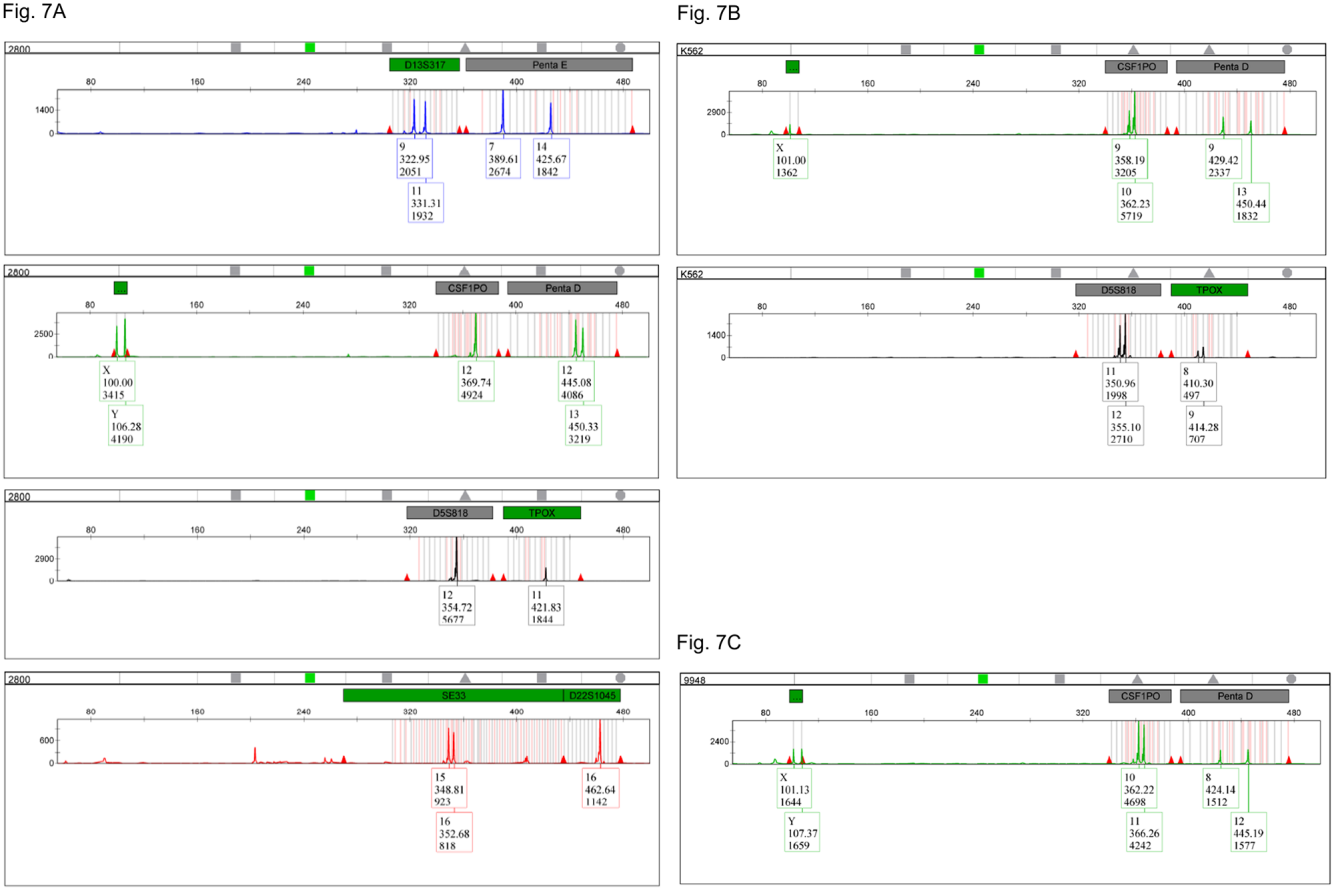
Electropherogram profile of control gDNA amplification with the long ssDNA polynucleotide nonaplex containing eight STR markers plus amelogenin. (A) 2800M DNA (all channels). (B) K562 DNA showing peak imbalances for CSF1PO, D5S818 and TPOX (JOE and TAMRA channels). (C) GM09948 DNA showing peak imbalances for CSF1PO (JOE channel).

In order to demonstrate how the long primers work with a larger, more forensically relevant multiplex, we then designed a nonaplex mixture containing eighteen primers. It encompasses eight long pair of primers that amplify the STR markers CSF1PO, Penta E, D5S818, D13S317, Penta D, TPOX, SE33 and D22S1045 and one pair of short primers that amplify the gender marker amelogenin. The nonaplex mixture containing several long primers of 60 and 200 nucleotides produced clean and informative CE profiles (Fig 7A). The short, standard primers used for amplifying the amelogenin marker showed to be fully compatible with the longer primers.

We also tested the polynucleotide mixture with some other DNA standards from well-established cell lines: K562, GM09947A and GM09948. We observed all the expected allele sizes for all these DNA standards as well as the characteristic heterozygous peak imbalances for markers CS1PO (dominant allele 10), D5S818 (dominant allele 12) and TPOX (dominant allele 9) in cell line K562 [31] (Fig 7B) and CSF1PO (dominant allele 10) in cell line GM09948 [32] (Fig 7C).

We used the nonaplex mixture to amplify 1ng of the four degraded samples that bear a known genotype (#62, #69, #71 and A2624). We then compared these results with the ones that had previously been obtained with the commercial kit PowerPlex^®^ Fusion and our own triplex mixture. All the alleles obtained with the nonaplex agreed with the ones previously assigned by the long polynucleotide triplex. They also matched with the already known genotypes. Additionally, many new alleles that were missing from the analysis with the commercial STR kit were now determined (Table 5).

**Table 5:** Assigned genotypes for CSF1PO, PENTA E, D5S818, D13S317, Penta D, TPOX, SE33, D22S1045 and amelogenin markers for 1ng of degraded cadaveric samples using PowerPlex^®^ Fusion and the long ssDNA polynucleotide nonaplex.

	Sample#62		Sample#69		Sample#71		Sample A2624	
	PowerPlex^®^ Fusion	Nonaplex	PowerPlex^®^ Fusion	Nonaplex	PowerPlex^®^ Fusion	Nonaplex	PowerPlex^®^ Fusion	Nonaplex
D13S317	9,9	9,**11**^a^	12,12	12,**13**	11,11^c^	-	-	**11,12**
Penta E	-	*15*,*17*^b^	-	**7,16**	-	*7*,*13*	-	**13,14**
CSF1PO	11,11	*10*,11	-	**10,13**	-	*11*,*11*	-	**12,12**
Penta D	-	-	12,13	12,13	-	-	-	**10,13**
Amelogenin	-	**Y?**	X,Y	X,Y	X,Y	-	X,X	X,X
D5S818	-	**11,12**	11,11	11,11	-	-	-	**13,13**
TPOX	-		-	**11,11**	-	-	-	-
SE33	NA	17,17	NA	**22,2,28.2**	NA	-	NA	14,27.2
D22S1045	-	**15,15**	15	**13,13**	-	-	-	-

^**a**^Alleles obtained only with the nonaplex mixture are highlighted in bold.

^b^Alleles obtained only with the triplex mixture are in italics.

^c^Alleles obtained only with the PowerPlex^**®**^ Fusion mixture are in gray.

(-) Absence of amplification.

Since the effective template concentration available for amplification in degraded DNA samples may actually be low, we then tested these same samples with the nonaplex mixture and the commercial kit PowerPlex^**®**^ Fusion 6C, but increasing the input DNA by 10 fold (10ng). These improved the allele assignment for both systems, but only the nonaplex mixture was able to assign most alleles for some of the degraded samples, e.g. #71 and A2624 (Table 6).

**Table 6:** Assigned genotypes for CSF1PO, PENTA E, D5S818, D13S317, Penta D, TPOX, SE33, D22S1045 and amelogenin markers for 10ng of degraded cadaveric samples using PowerPlex^®^ Fusion 6C and the long ssDNA polynucleotide nonaplex.

	Sample#62		Sample#69		Sample#71		Sample A2624	
	PowerPlex^®^ Fusion 6C	Nonaplex	PowerPlex^®^ Fusion 6C	Nonaplex	PowerPlex^®^ Fusion 6C	Nonaplex	PowerPlex^®^ Fusion 6C	Nonaplex
D13S317	9,9	9,**11**^a^	12,13	12,13	11,11	11,11	-	**11,12**
Penta E	15,17	15,17	7,16	7,16	-	*7*,*13*^b^	-	**13,14**
CSF1PO	10,11	10,11	10,13	10,13	-	**11,11**	-	**12,12**
Penta D	9,11	9,11	12,13	12,13	-	-	-	**10,13**
Amelogenin	X,Y	X,Y	X,Y	X,Y	X,Y	X,Y	X,X	X,X
D5S818	11,12	11,12	11,11	11,11	-	**13,13**	-	**13,13**
TPOX	8,12	8,12	11,11	11,11	-	-	-	**8,11**
SE33	17,17	17,17	22.2,28.2	22.2,28.2	-	16,23.2	14,27.2	14,27.2
D22S1045	15,15	15,15	12,15	12,15	-	-	-	-

^**a**^Additional alleles obtained with the nonaplex mixture are highlighted in bold.

^b^Allele pair obtained only with the triplex mixture are in italics.

(-) Absence of amplification.

**Fig 8:**
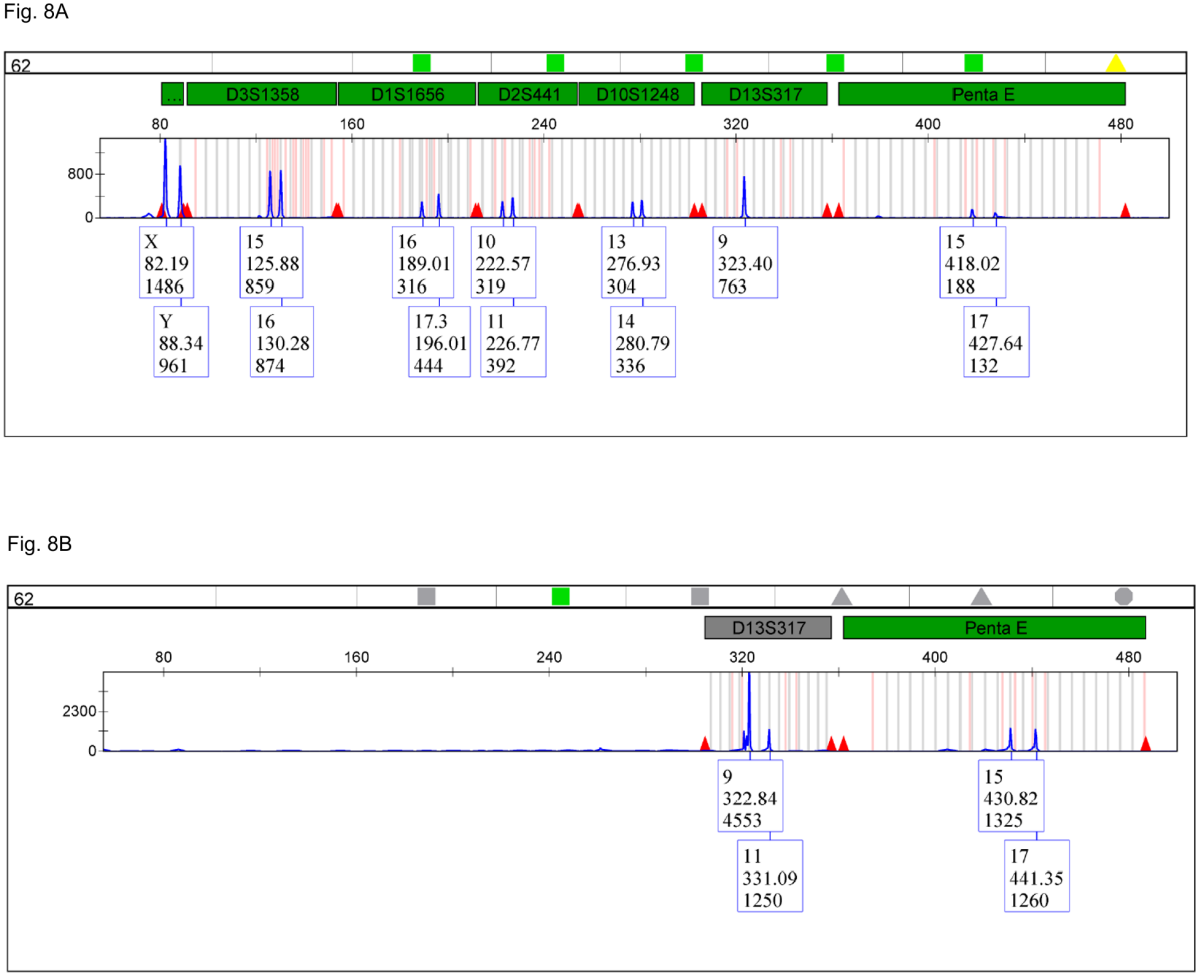
Electropherogram for cadaveric sample #62 amplified with PowerPlex^®^ Fusion 6C vs. the long ssDNA polynucleotide nonaplex. (A) Amplification results with PowerPlex^®^ Fusion 6C showing a single allele (9) for the D13S317 *locus* (Fluorescein channel). (B) Amplification results with the long ssDNA polynucleotide nonaplex showing both alleles (9, 11) for the D13S317 *locus* (Fluorescein channel).

Even for the more complete profile of sample #62 the nonaplex mixture was able to detect the missing allele 11 for marker D13S317 (Fig 8).

### *In silico* analysis of primer interactions

An *in-silico* analysis using the AutoDimer software developed by the NIST [29] was performed to assess any potential interactions that may occur between the unusually long primers included in the triplex and nonaplex multiplex mixtures. Only one hit, involving the self-annealing of the unlabeled primer PEM1325FW (Table 7, first row), was detected for the triplex assay. That interaction was not observed in the CE electropherogram (Fig 5A).

**Table 7:** Potential primer-dimer structures within and between the triplex primers and the primers listed in the NIST STRBase website.

Primer- dimer	Matches	Score	Estimated Tm (°C)	Estimated ΔG _37°_ (kcal/mole)	Stretch (bp)	Fluorescent detection
PEM1325FW / PEM1325FW	16	8	95.4	-17.95	5	No
Ame.PP16R^a^ / PEM1325FW	16	8	100.2	-23.24	9	No
D7S820.PP16F^b^ / PE26RV**FL**	9	7	74.7	-13.21	8	Yes (FL/JOE)
D7S820.PP16F / D5S60RV**TM**	14	7	86.0	-17.52	6	Yes (TMR/JOE)

^**a**^Ame.PP16R: atcagagcttaaactgggaagctg

^b^D7S820.PP16F: JOE/atgttggtcaggctgactatg

In order to assess if these long primers might interact with primers regularly used in STR analysis, we verified their interaction with the ones listed on the NIST STRBase website [12]. Only 3 additional hits were detected from a total of 741 primer-primer comparisons. They involved only two primers: PEM1325FW and D7S820.PP16F (Table 7).

We also determined the potential interactions for all the primers present in the nonaplex mixture, including the amelogenin pair. Some possible primer complementarities (7 out of 171) were found (Table 8), although they were not detected in the CE (Fig 7).

**Table 8:** Potential primer-dimer structures within the nonaplex primers.

Primer- dimer	Matches	Score	Estimated T_m_ (°C)	Estimated ΔG_37°_ (kcal/mole)	Stretch (bp)	Fluorescent detection
PEM1325FW—PEM1325FW	16	8	95.4	-17.95	5	No
AmelRV -PEM1325FW	16	8	100.2	-23.24	9	No
D5S60RVTM—D13M1325RVFL	16	8	100.9	-21.57	6	Yes (TMR/FL)
D22S200FW—D22S200FW	12	8	97.7	-16.82	4	No
D22S60RVROX- D22S60RVROX	12	7	102.6	-18.32	5	Yes (ROX)
D22S60RVROX—TPOX200RV	22	7	133.0	-28.65	5	Yes (ROX)
TPOX60FWTM—TPOX60FWTM	14	8	104.2	-18.38	4	Yes (TMR)

## Discussion

Our study shows the advantage of using long ssDNA polynucleotides as primer surrogates for obtaining STR profiles in degraded DNA samples. By annealing closer to the repeats, these long primers extend much shorter DNA sequences than the standard primers, making them ideal to amplify fragmented DNA, working in a similar way to the mini-STRs. Differently from the mini-STRs, the long primer sequences incorporated into the PCR products make the resulting amplicons suitable for the longer and more diverse product sizes required for CE in multiplex assays.

We have not observed any primer-dimer formation in the long ssDNA polynucleotide reactions, in concordance with previous results obtained from a different long primer triplex (CSF1PO/Penta E/DYS391) [20, 21]. We have not found any major artifacts on agarose gels, qPCR or CE profiles in either the samples or in the no template controls (NTC)–although there is a non-specific peak for CSF1PO in the CE profile at approximately 120bp. More importantly, no special setup or configuration was needed, we have just employed regular PCR solutions and conditions without any further adjustments. We have even used a regular annealing temperature of 59°C to make the reaction fully compatible with standard, short primers. This lack of artifacts had also been reported when spiking a long primer pair to existing kits containing PCR mixtures of 42 and 54 primers [20, 21]. These observations seem to support the idea that more complex multiplex assays may be readily designed by the addition of more primers of different sequences and sizes.

We have also shown that long primers that are either totally complementary or only complementary at the 3’-priming region of the template sequence can be successfully employed. The use of non-homologous sequences may be important for providing flexibility in designing multiplex reactions that require several primers. We have actually used M13 phage sequences in a few of our multiplex primers. The use of primers with non-complementary sequences in the 5’ region—which is not involved in priming—might be important in designing multiplex reactions containing several primers by allowing the use of virtually any sequence of choice.

While we have seen that primers of standard size are more effective for amplifying intact DNA, the advantage of using long primers that anneal next to the target regions is evident for degraded DNA. Moreover, since the long primers are designed to anneal adjacently to the repeats, they prevent the incorporation into the final PCR product of deletions or insertions that may be present in the outlying sequence. This feature makes them a reliable alternative to avoid wrong allele assignments in intact and reference DNA samples as well.

We have demonstrated that several allele sequences present in degraded DNA samples, otherwise not detected by the commercial kits, can be readily discriminated by using long ssDNA polynucleotides. While we have designed the long primer sequences to anneal close to the repeats, these results in some cases could be further improved by using primers slightly longer than 200 nucleotides, capable to anneal exactly at the extremes of the repeat regions. Longer primers of approximately 250 nucleotides or more could be prepared *ad-hoc* for this purpose by means of synthetic chemistry, e.g. Megamers^™^ (IDT) or by PCR amplification followed by strand separation [33].

Alternative methods such as Next Generation Sequencing (NGS) are being developed for forensic applications with degraded DNA (actually fragmentation is a necessary step in NGS sample processing), but they have their own, different challenges [34]. The present study relies in the simplicity of the CE technology, which is the universal method and the gold standard for genetic identity analyses.

The incorporation of long primers to amplify large amplicons in multiplex STR kits may overcome the issues posed by the degradation of DNA for obtaining complete and reliable individual genotype profiles in forensic, aged or archaeological samples. Additionally, long primers may also be employed in applications involving other type of fragmented DNA sequences, for instance in oncogenic and pregnancy testing from circulating cell-free DNA (ccfDNA).

## Supporting information

S1 File Ref [20] Specification USPTO application 15/283,851. (PDF)

S2 File Ref [20] Drawings USPTO application 15/283,851. (PDF)

S3 File Ref [21] Specification and drawings PCT application EP2017/067189. (PDF)
