# Supplementary material for: Using long ssDNA polynucleotides to amplify STRs loci in degraded DNA samples
Source: PLoS One. 2017 Nov 3;12(11):e0187190. doi: 10.1371/journal.pone.0187190 (PMC5669423; doi:10.1371/journal.pone.0187190)
Supplement: S2 File — (PDF) [file pone.0187190.s002.pdf]

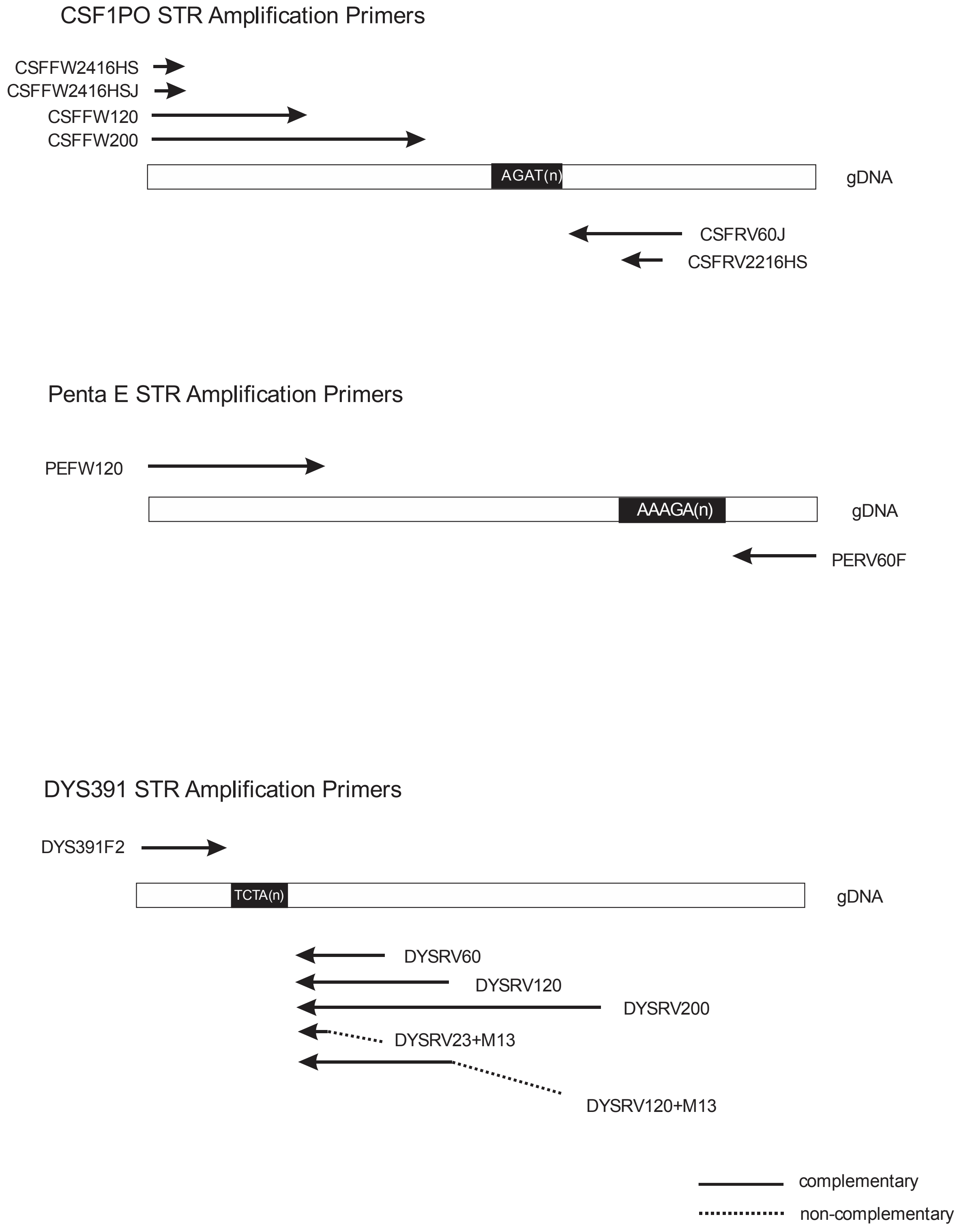
CSF1PO STR Amplification Primers
CSFFW2416HS
CSFFW2416HSJ
CSFFW120
CSFFW200
AGAT(n)
gDNA
CSFRV60J
CSFRV2216HS
Penta E STR Amplification Primers
PEFW120
AAAGA(n)
gDNA
PERV60F
DYS391 STR Amplification Primers
DYS391F2
TCTA(n)
gDNA
DYSRV60
DYSRV120
DYSRV200
DYSRV23+M13
DYSRV120+M13
complementary
non-complementary

Figure 1

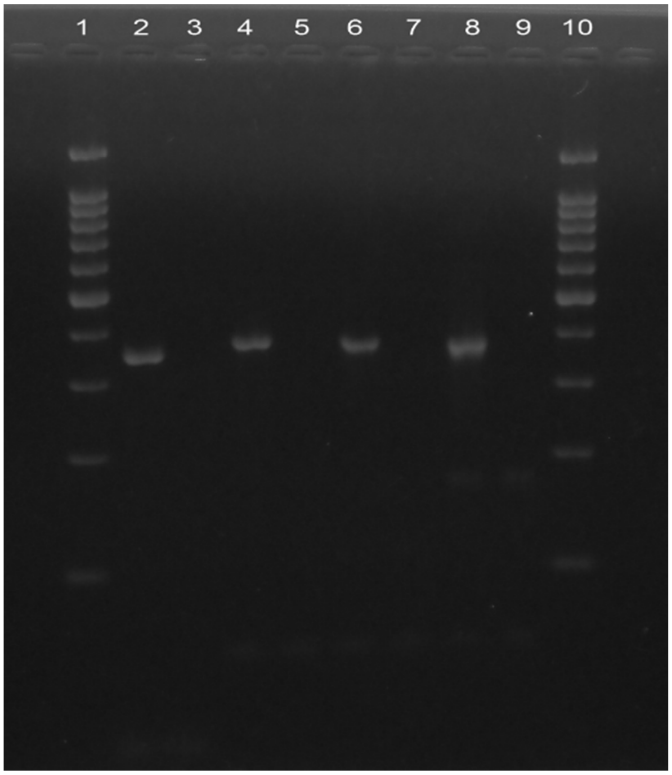

Figure 2A

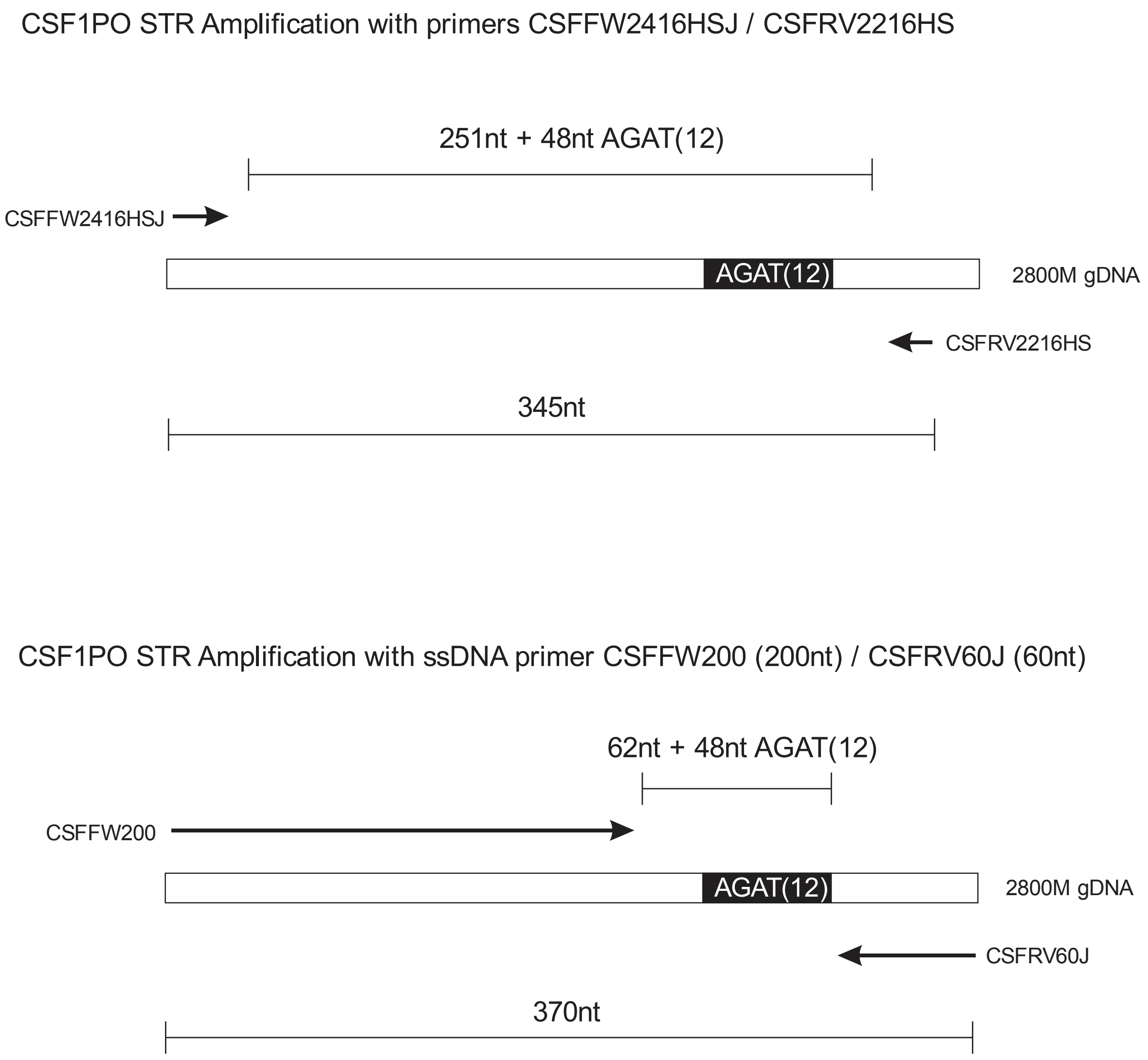

Figure 2B

Chromatogram #1 (2800M)

JOE Channel

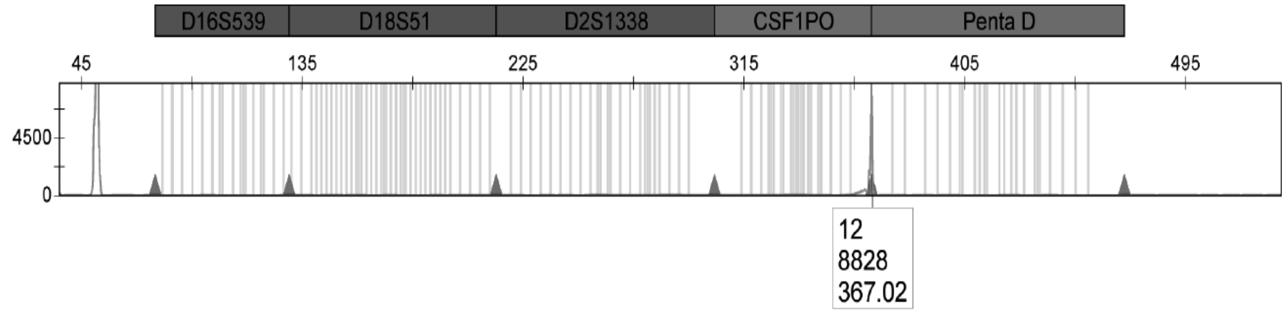

Figure 3A (continued)

Chromatogram #2 (9947A)

JOE Channel

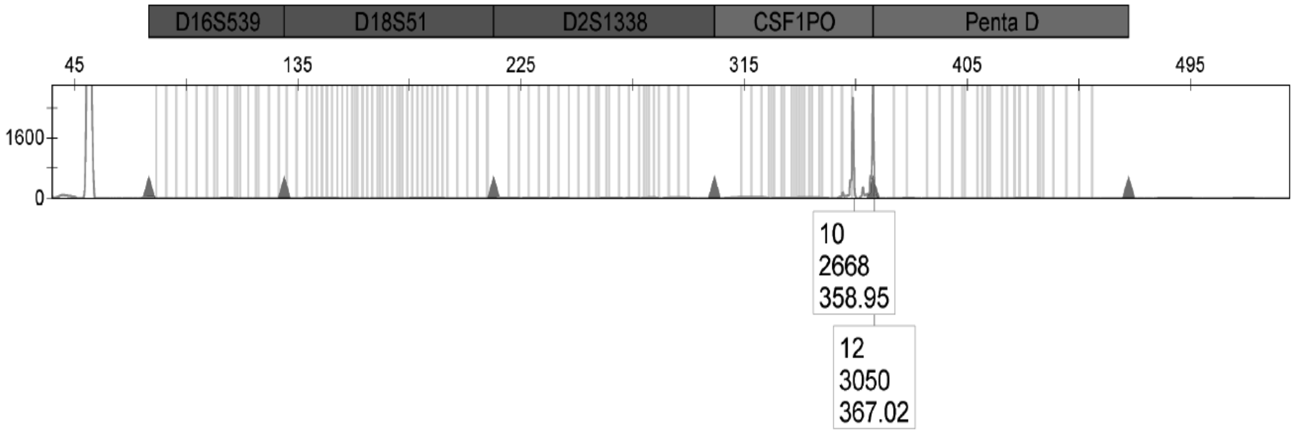

Chromatogram #3 (9948)

JOE Channel

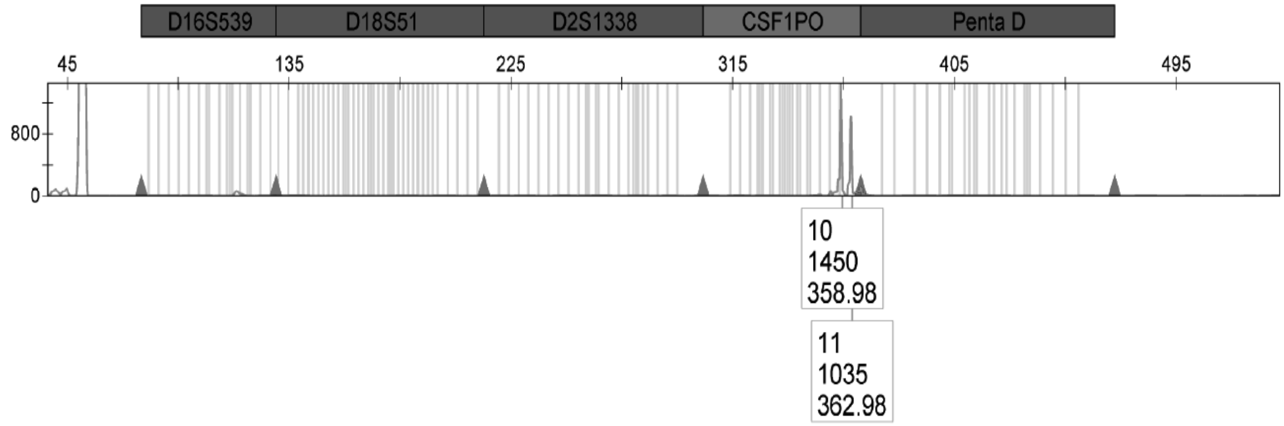

Figure 3A (continued)

Chromatogram #4 (K562)

JOE Channel

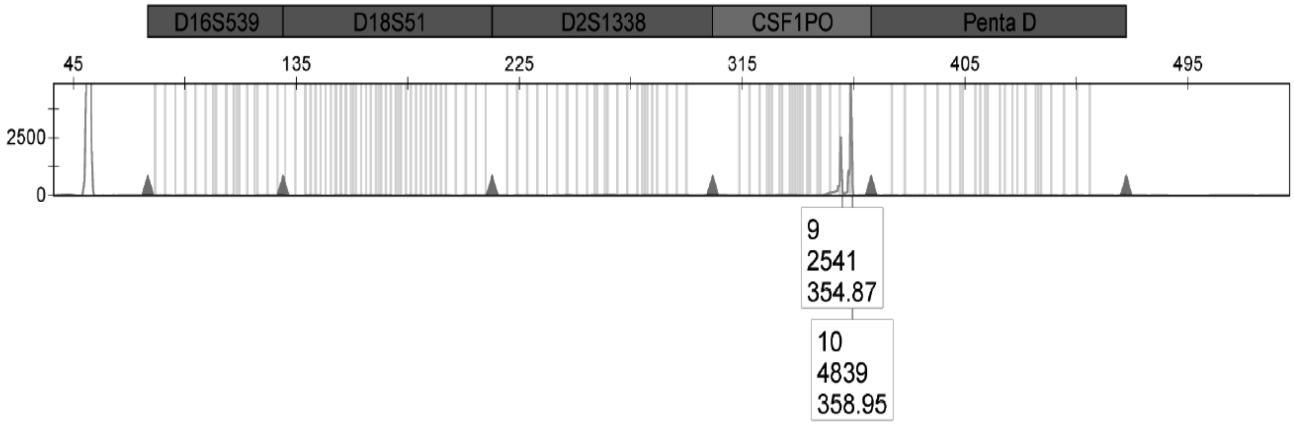

Chromatogram #5 (NTC)

JOE Channel

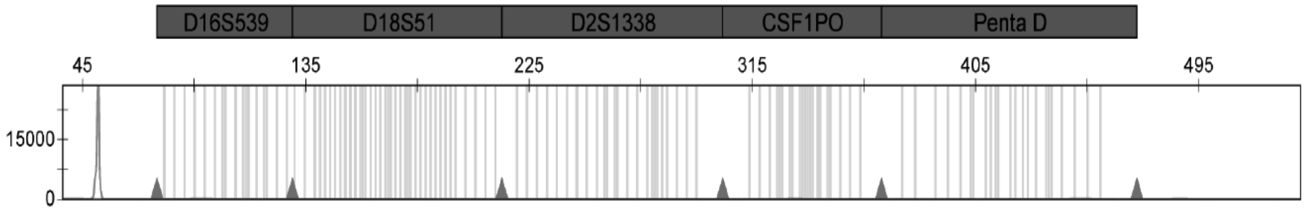

Chromatogram #6 (2800M)

JOE Channel

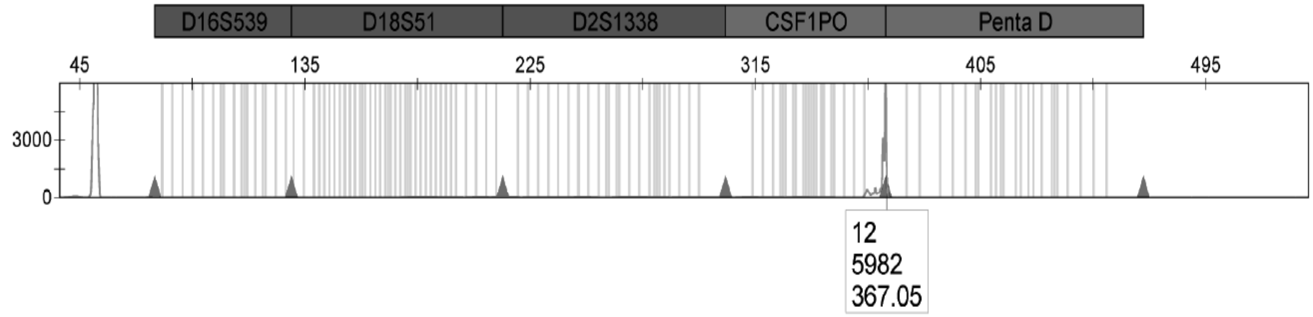

Figure 3A (continued)

Chromatogram #7 (9947A)

JOE Channel

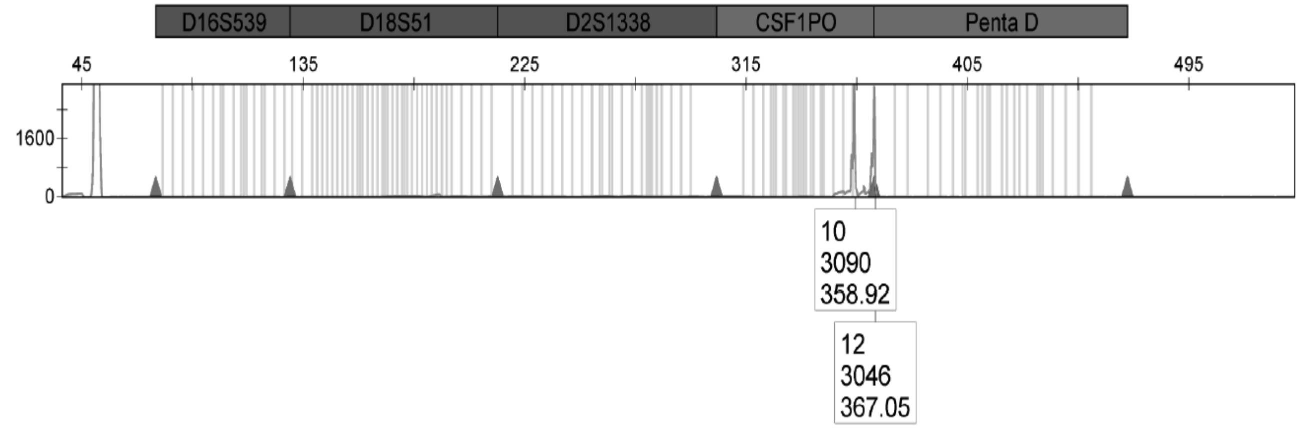

Chromatogram #8 (9948)

JOE Channel

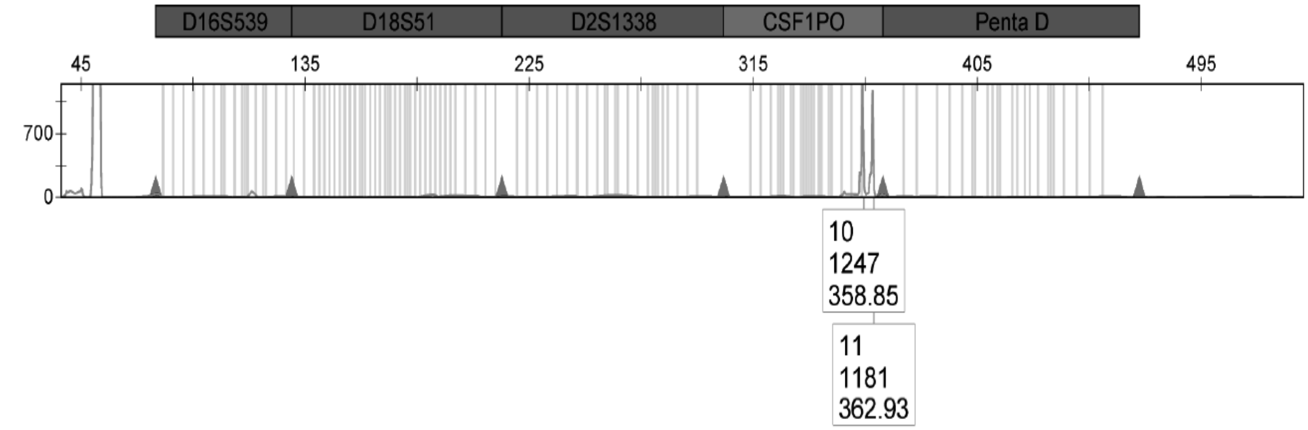

Figure 3A (continued)

Chromatogram #9 (K562)

JOE Channel

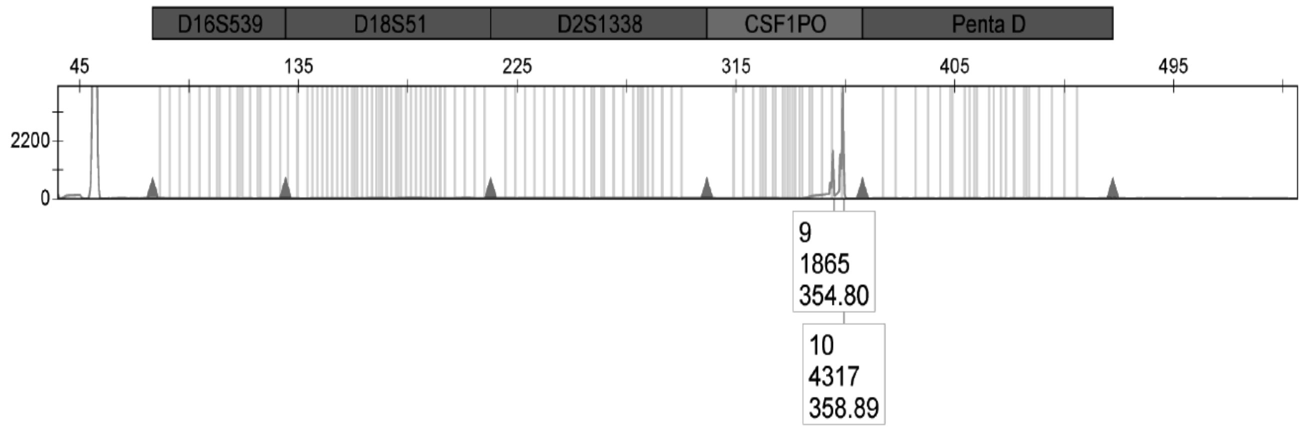

Chromatogram #10 (NTC)

JOE Channel

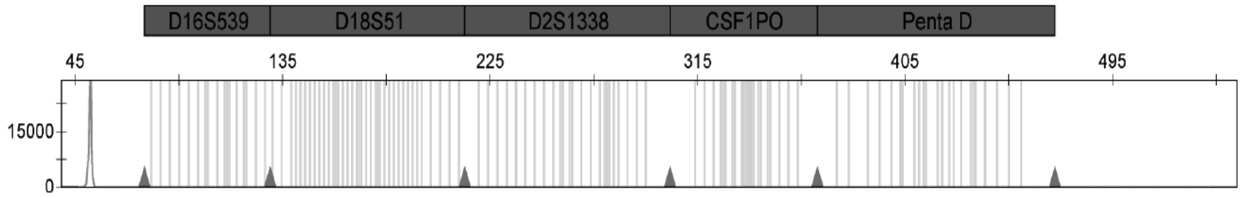

Figure 3A

Chromatogram #3 (inset enlargement)

JOE Channel

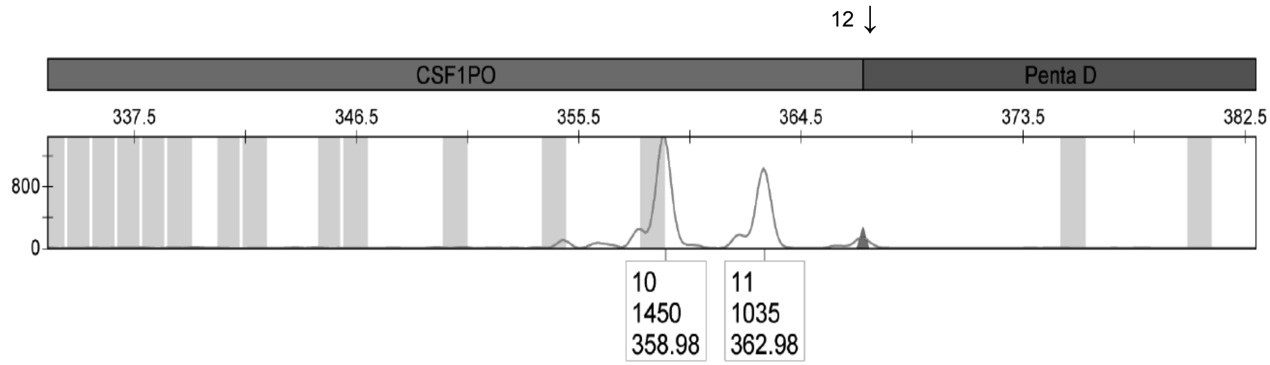

Figure 3B (continued)

Chromatogram #8 (inset enlargement)

JOE Channel

12 ↓

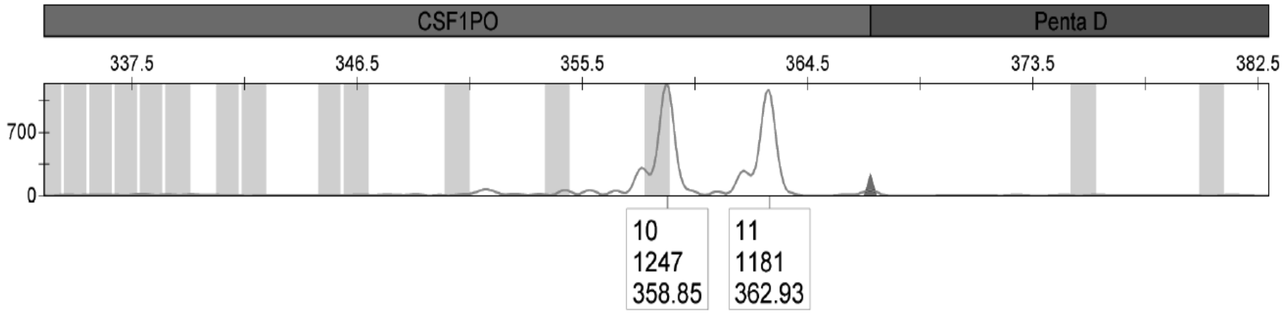

Figure 3B

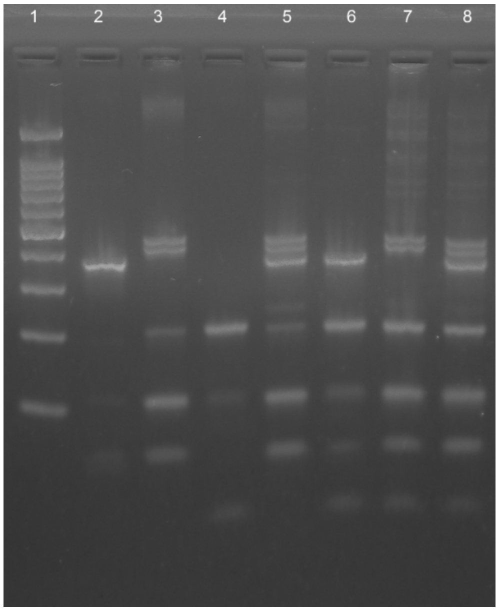

Figure 4A

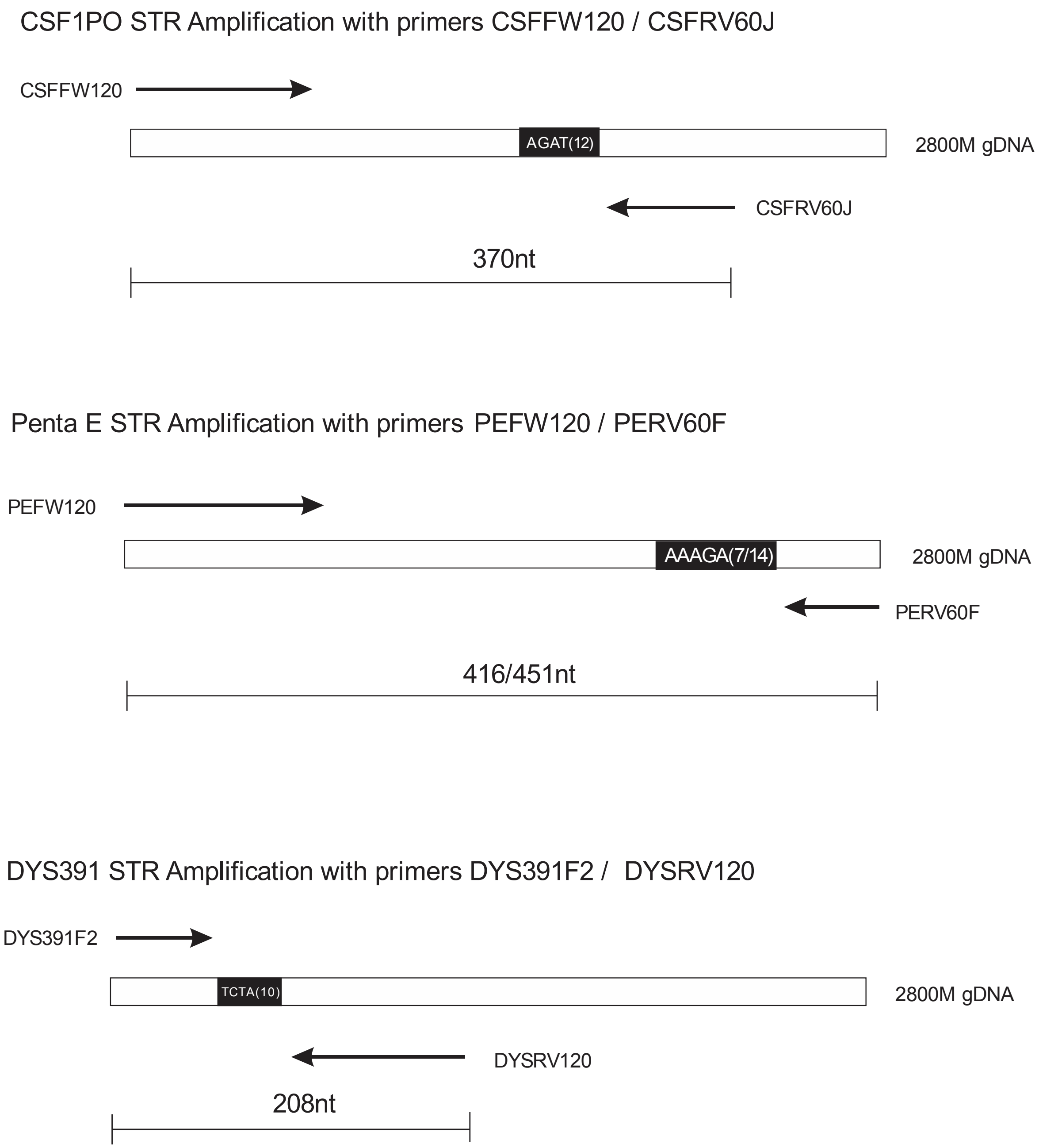
CSF1PO STR Amplification with primers CSFFW120 / CSFRV60J
CSFFW120
AGAT(12)
2800M gDNA
CSFRV60J
370nt
Penta E STR Amplification with primers PEFW120 / PERV60F
PEFW120
AAAGA(7/14)
2800M gDNA
PERV60F
416/451nt
DYS391 STR Amplification with primers DYS391F2 / DYSRV120
DYS391F2
TCTA(10)
2800M gDNA
DYSRV120
208nt

Figure 4B

FAM/Fluorescein Channel

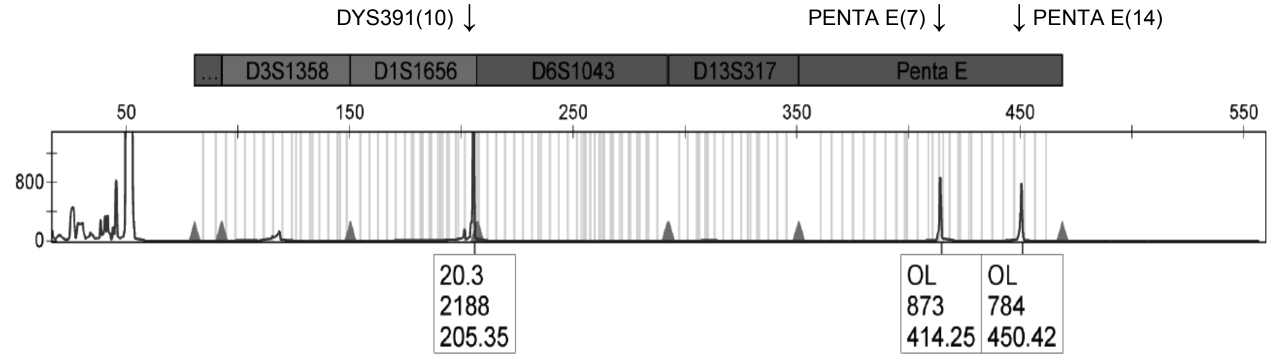

JOE Channel

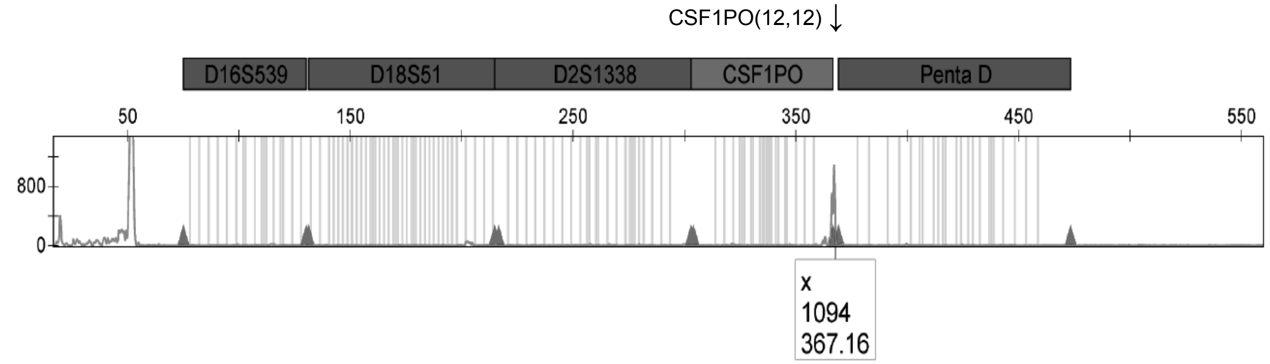

Figure 5

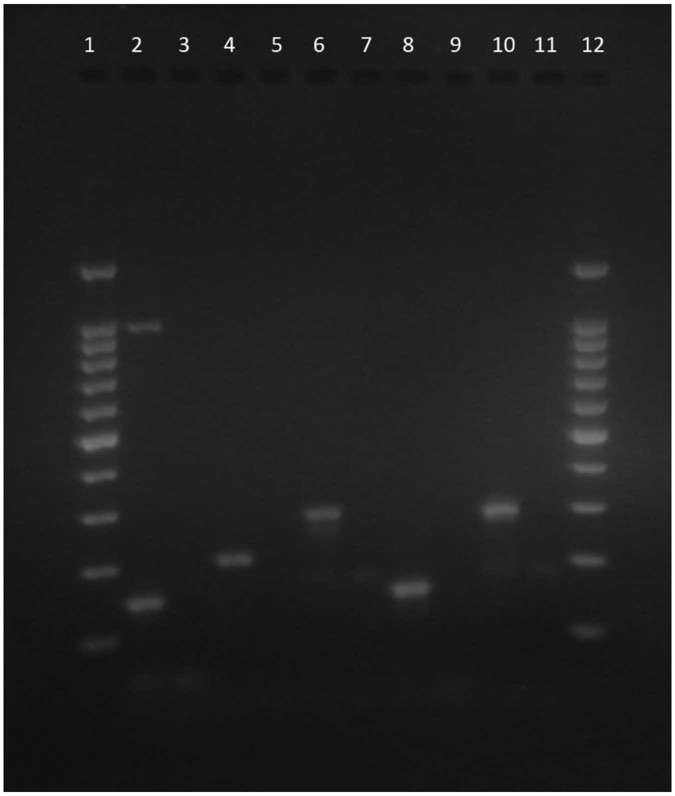

Figure 6

DYS391 STR Amplification with primers DYS391F2/ DYSRV120

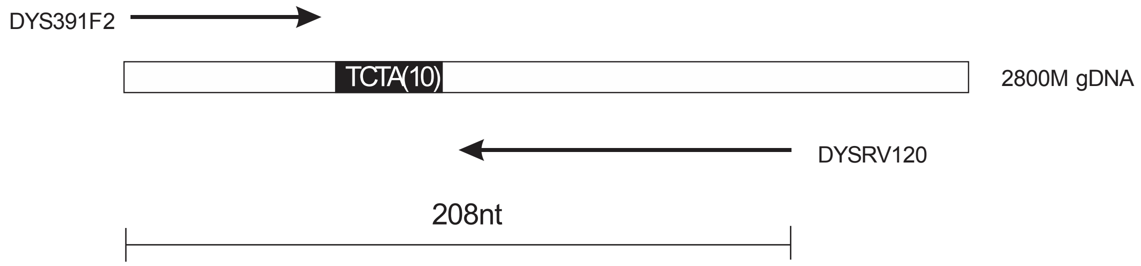

DYS391 STR Amplification with primers DYS391F2 / DYSRV200

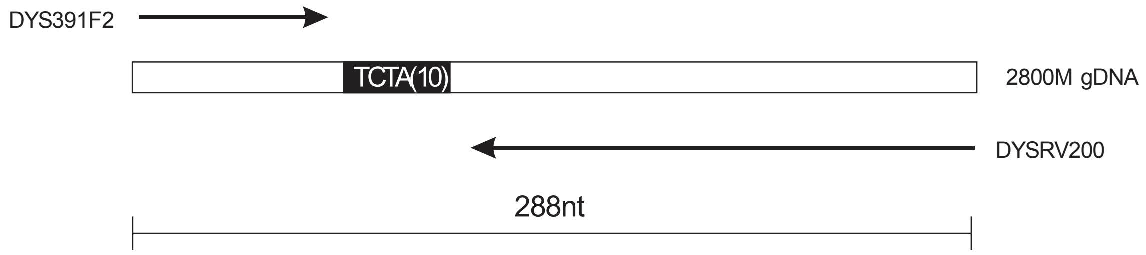

Figure 7A

Chromatogram #1 PowerPlex® 21

FAM/Fluorescein Channel

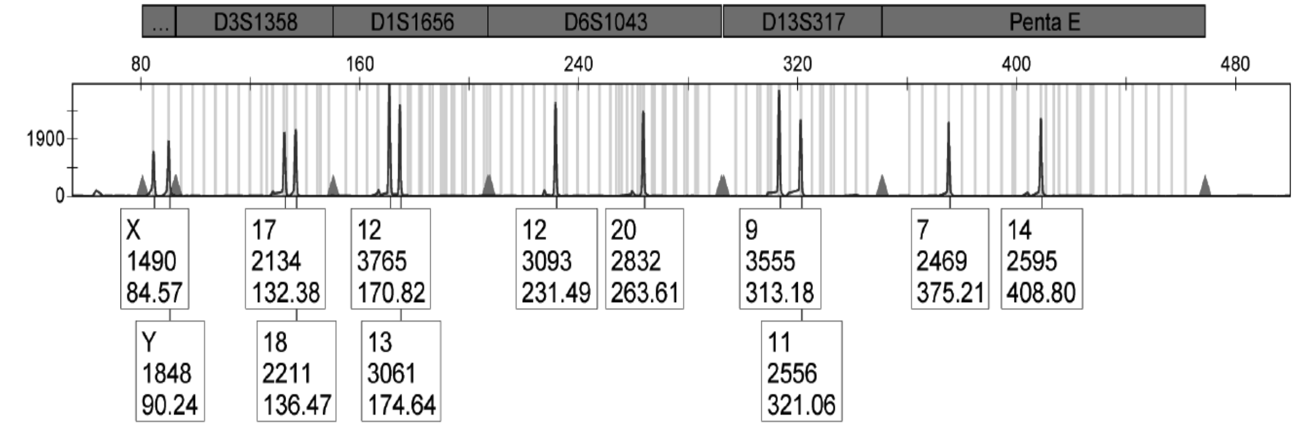

JOE Channel

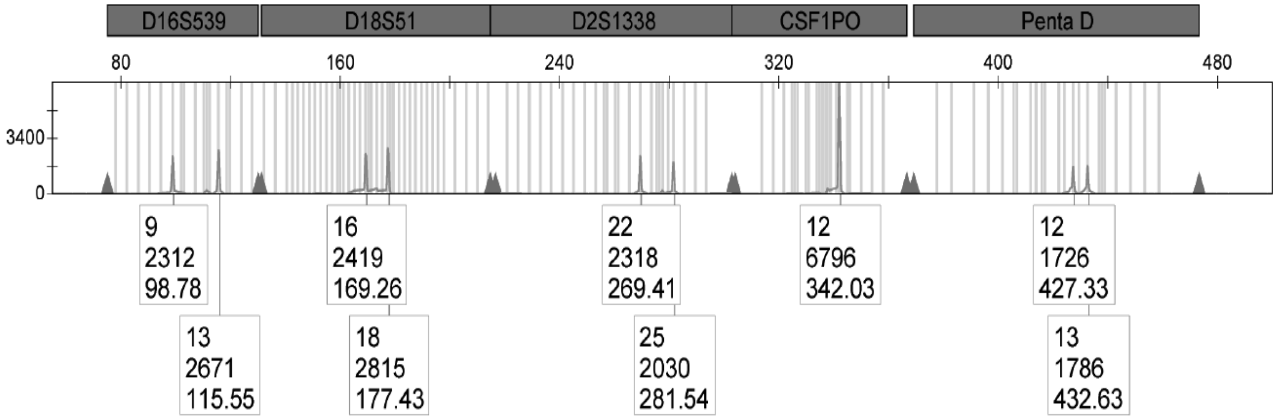

TMR Channel

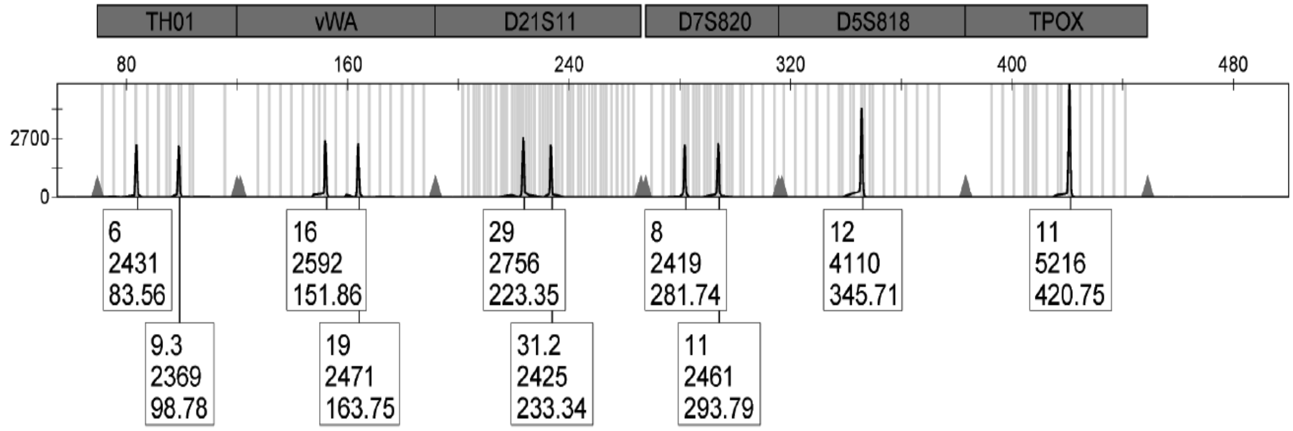

Figure 7B (continued)

CXR Channel

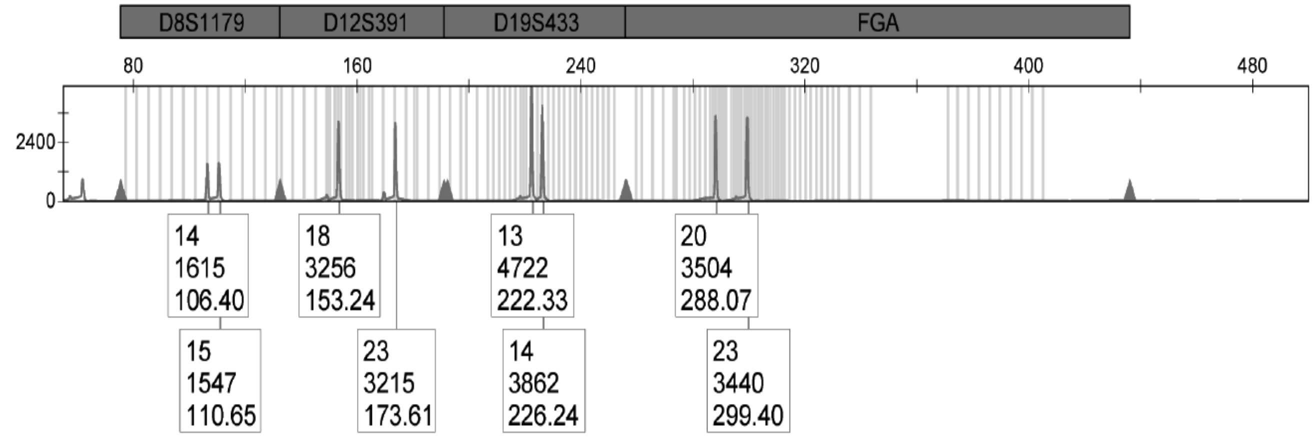

Chromatogram #2 PowerPlex® 21 + DYS391F2 / DYSRV120

FAM/Fluorescein Channel

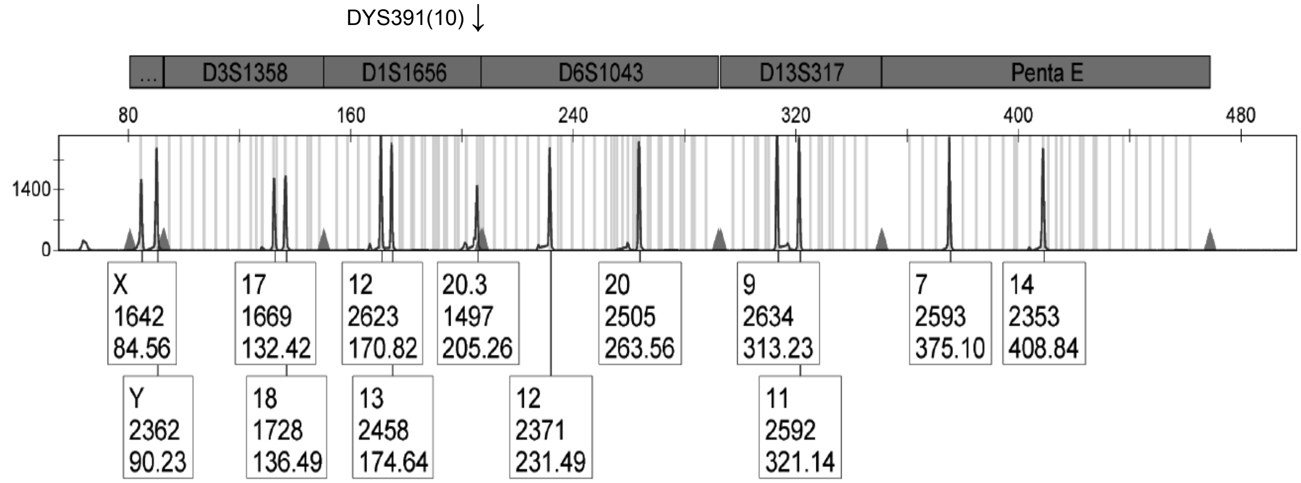

Figure 7B (continued)

JOE Channel

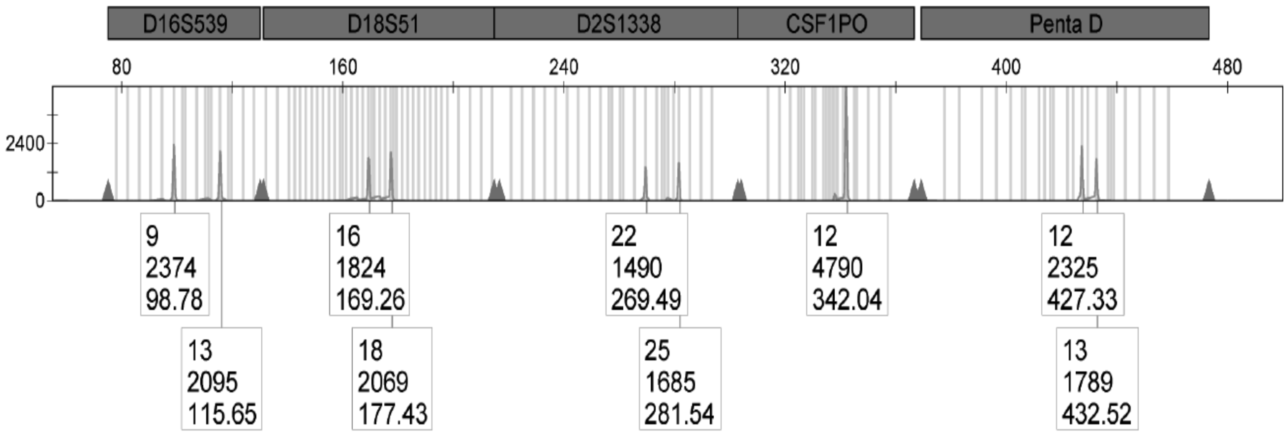

TMR Channel

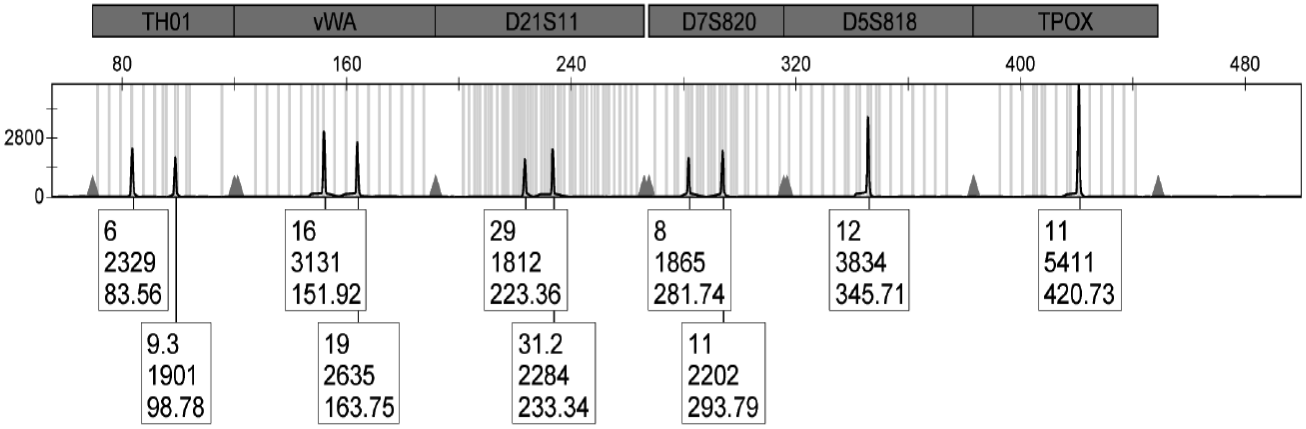

CXR Channel

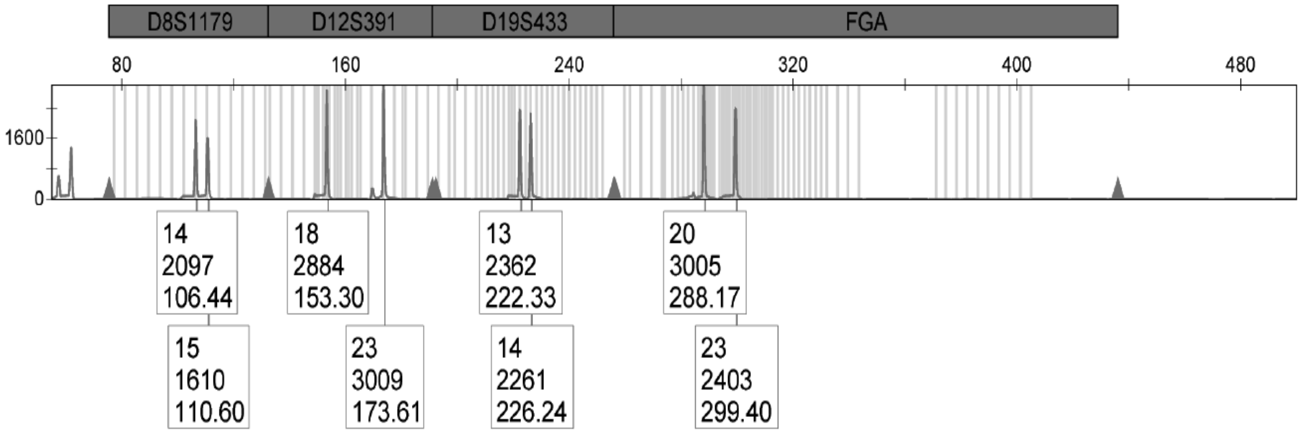

Figure 7B (continued)

Chromatogram #3 PowerPlex® 21 + DYS391F2 / DYSRV200

FAM/Fluorescein Channel

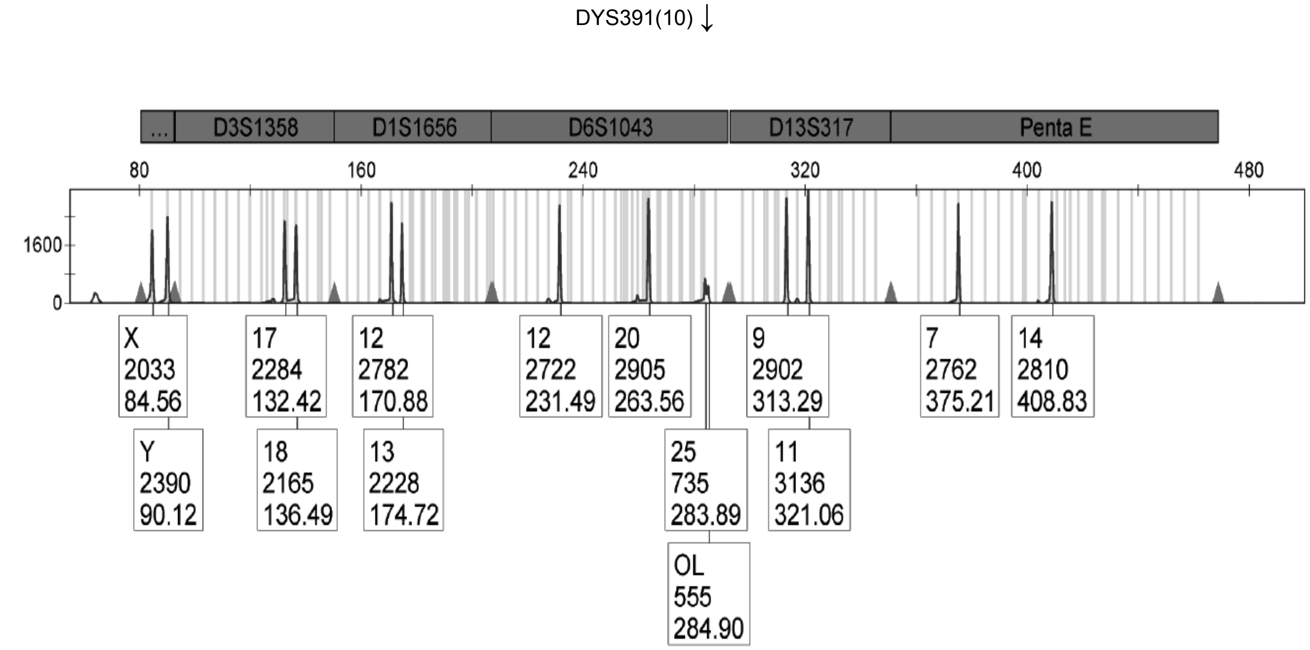

JOE Channel

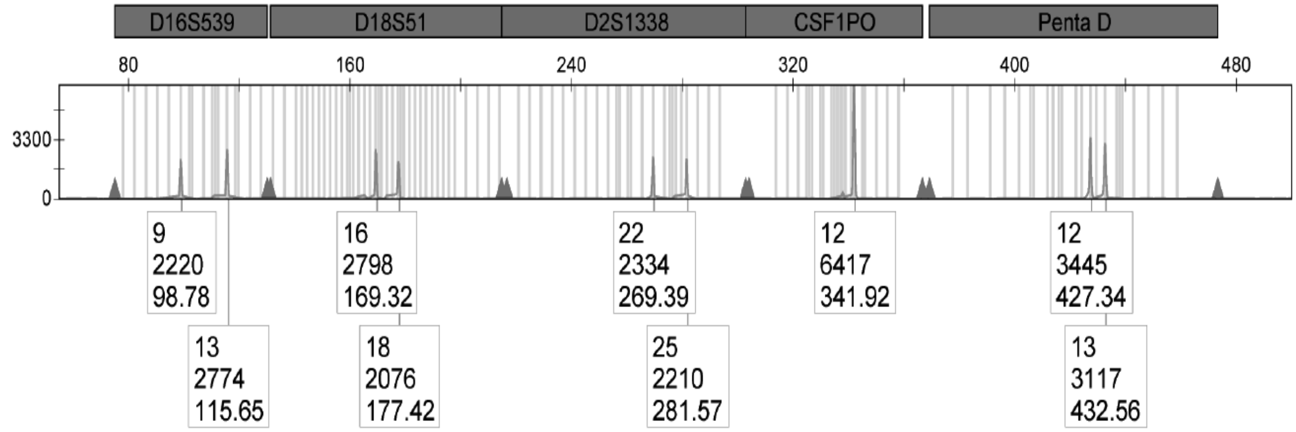

Figure 7B (continued)

TMR Channel

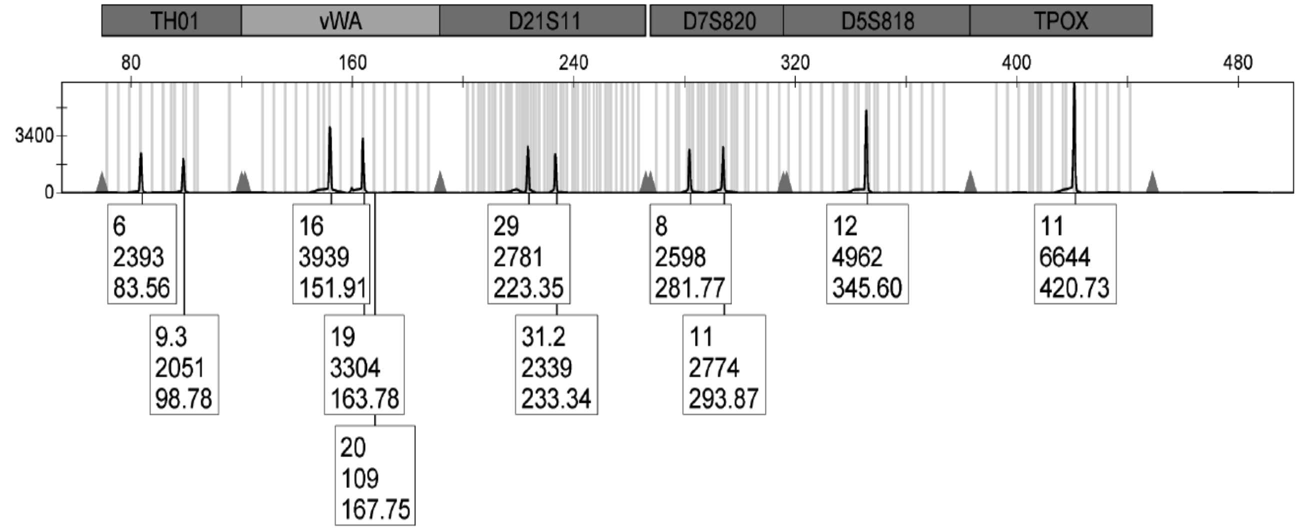

CXR Channel

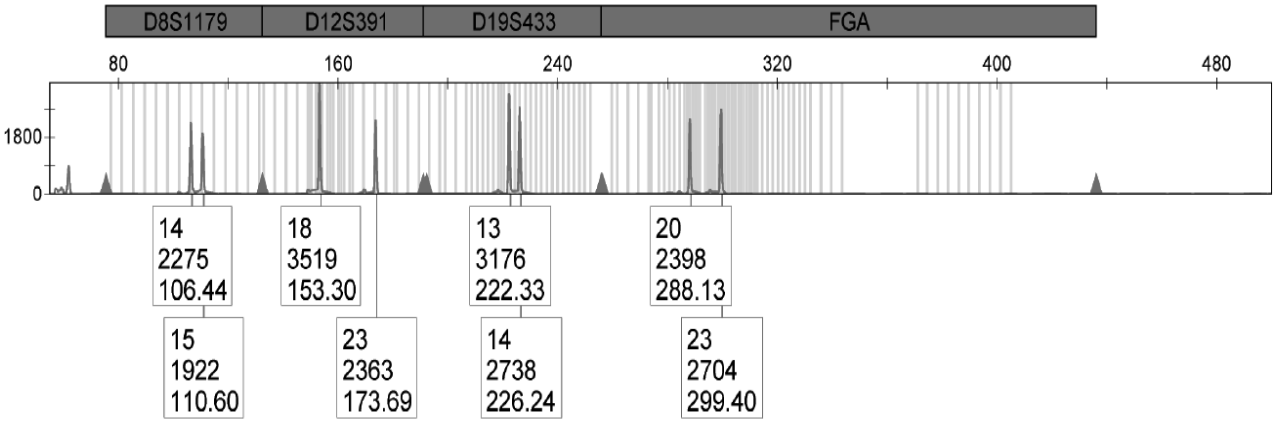

Figure 7

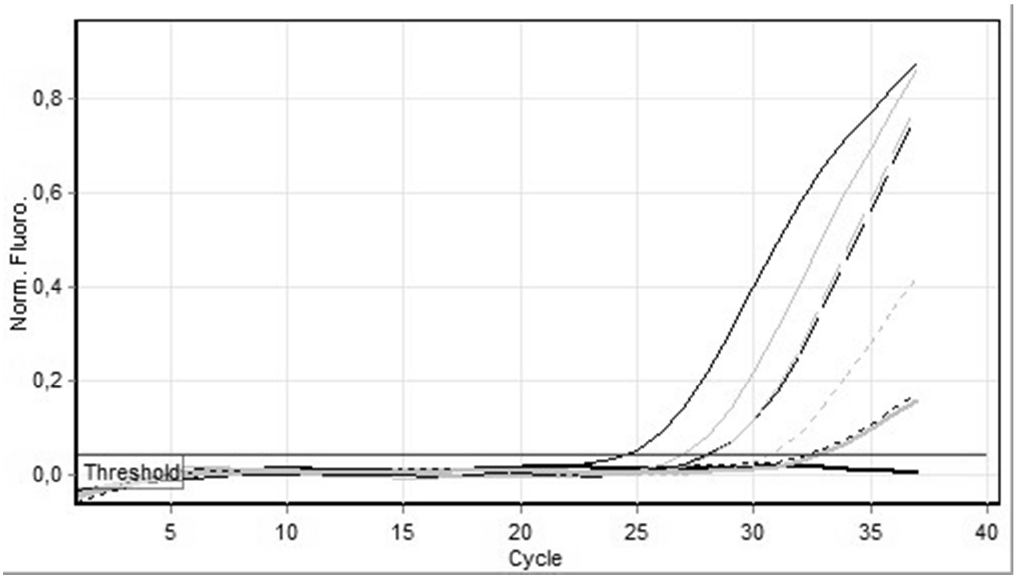

No.	Color	Pattern	Primers	Time (min)	Ct
1		Solid	CSFFW2416HSJ / CSFRV2216HS	0	24,53
2		Dashed	CSFFW2416HSJ / CSFRV2216HS	10	28,07
3		Dotted	CSFFW2416HSJ / CSFRV2216HS	30	32,39
4		Thin	CSFFW2416HSJ / CSFRV2216HS	NTC	
5		Solid	CSFFW200 / CSFRV60J	0	26,97
6		Dashed	CSFFW200 / CSFRV60J	10	28,23
7		Dotted	CSFFW200 / CSFRV60J	30	30,71
8		Thin	CSFFW200 / CSFRV60J	NTC	32,81

Figure 8

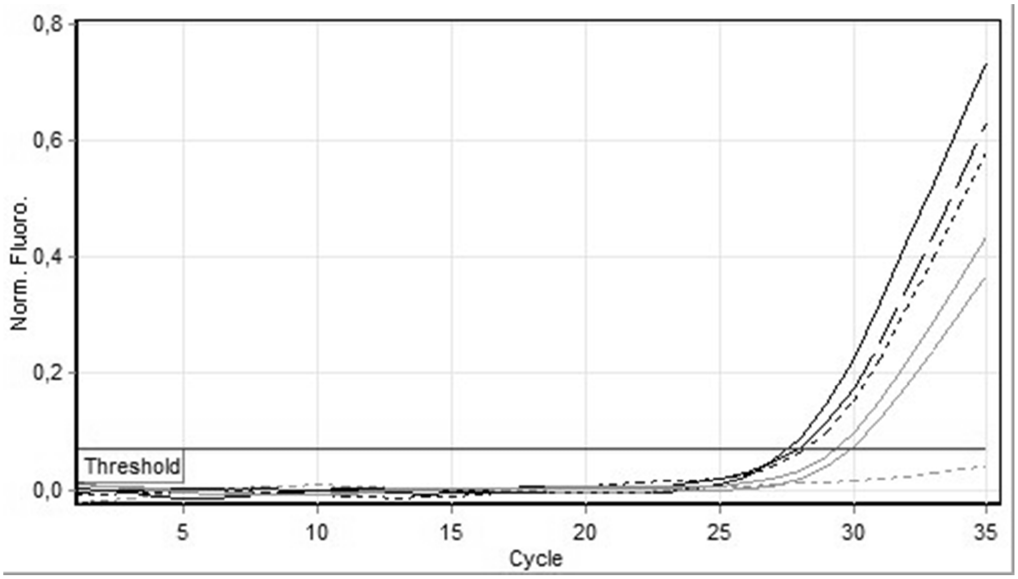

No.	Color	Pattern	Primers	Time (min)	Ct
1		Solid	CSFFW200 / CSFRV60J	0	27,59
2		Dashed	CSFFW200 / CSFRV60J	10	27,96
3		Dotted	CSFFW200 / CSFRV60J	30	28,27
4		Solid	CSFFW200 / CSFRV60J	45	29,37
5		Dashed	CSFFW200 / CSFRV60J	60	29,94
6		Dotted	CSFFW200 / CSFRV60J	NTC	

Figure 9A

Chromatogram #1 PowerPlex® Fusion 6C (0 min)

FAM/Fluorescein Channel

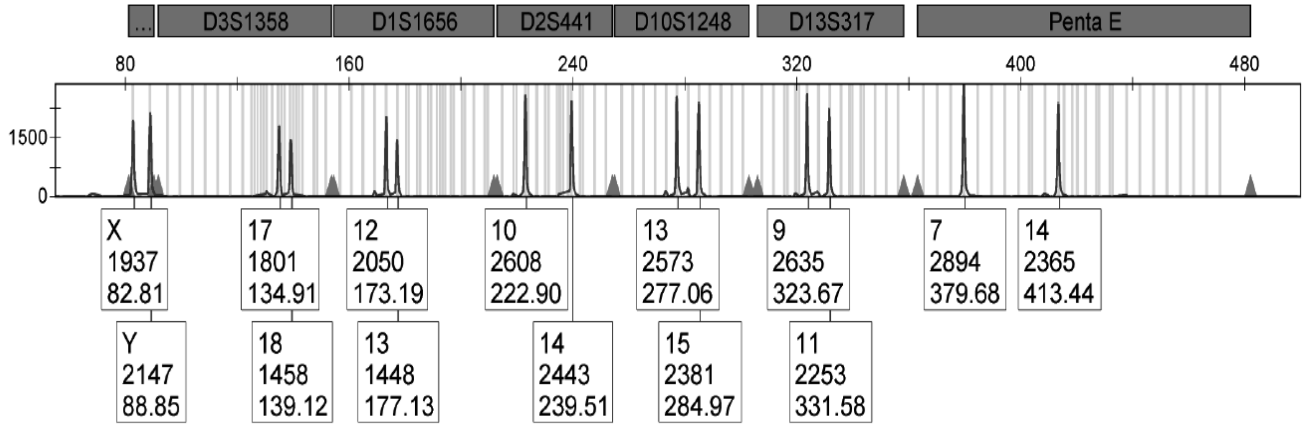

JOE Channel

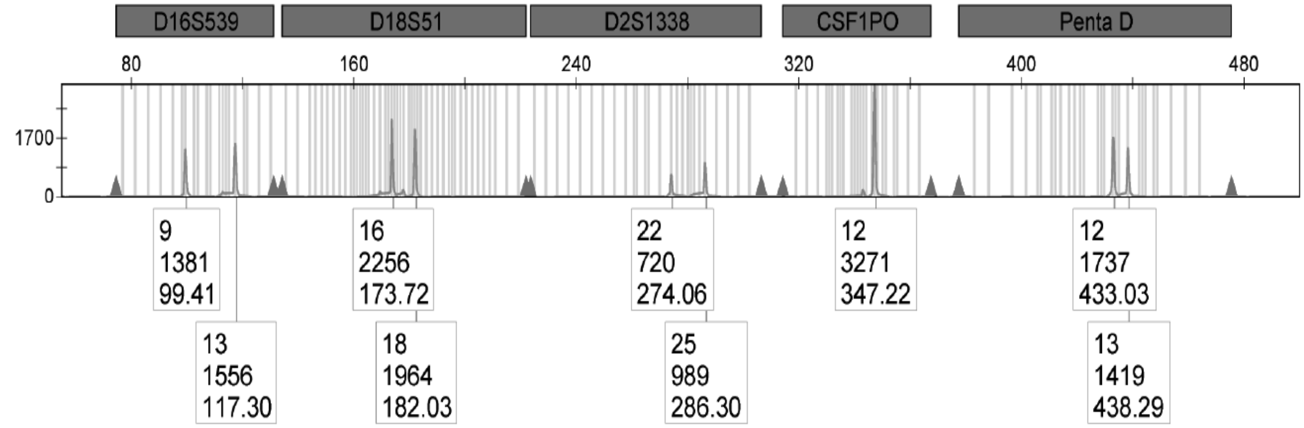

TMR Channel

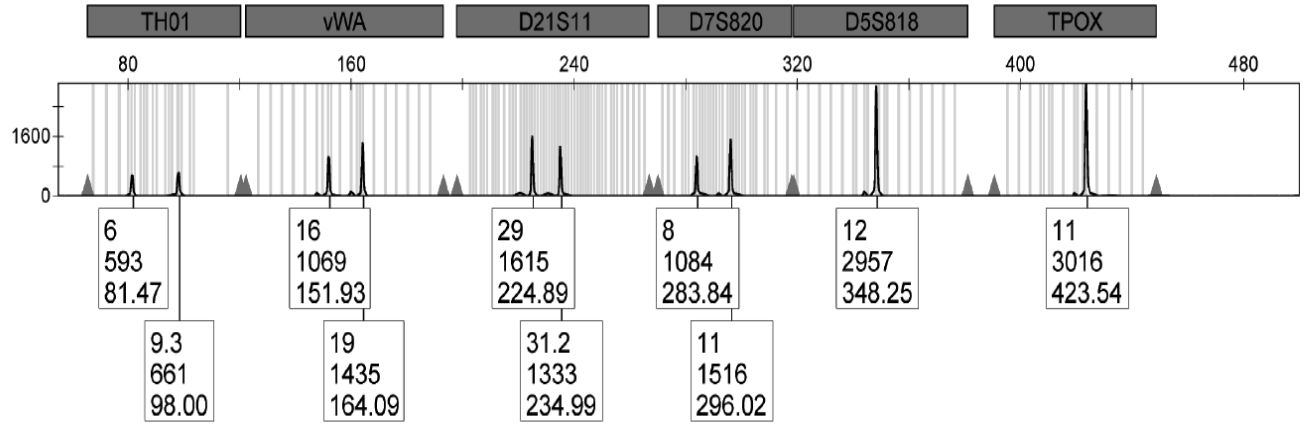

Figure 9B (continued)

CXR Channel

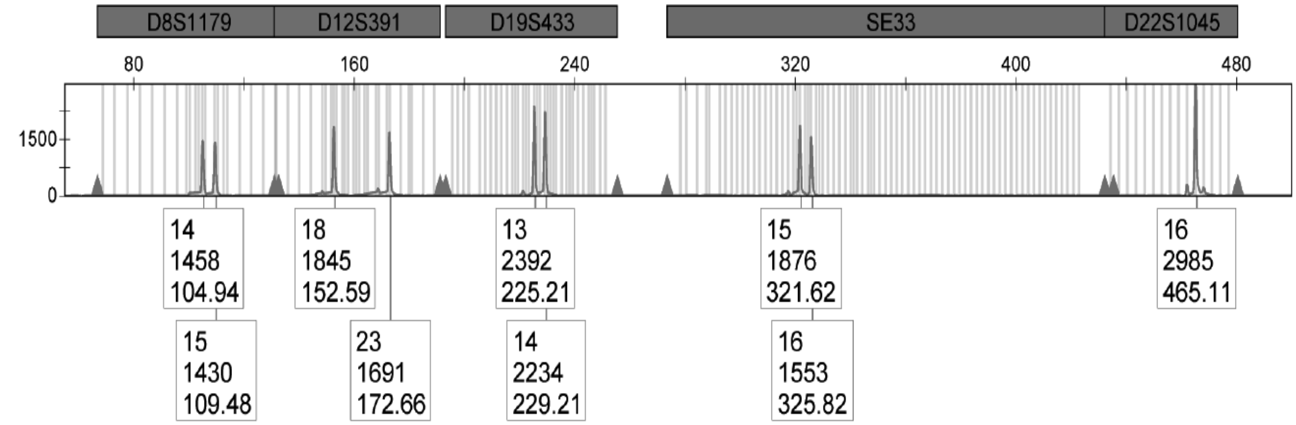

TOM Channel

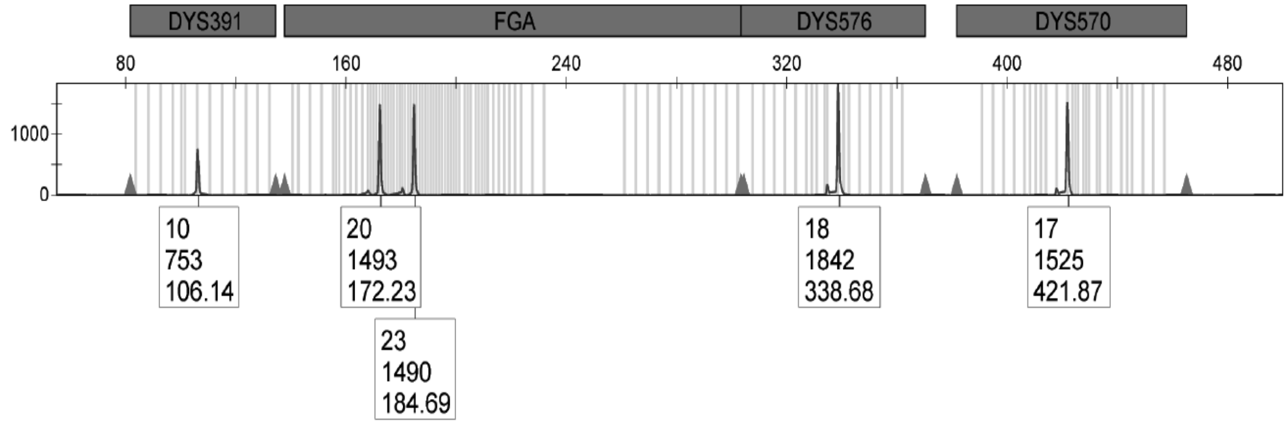

Chromatogram #2 PowerPlex® Fusion 6C (30 min)

FAM/Fluorescein Channel

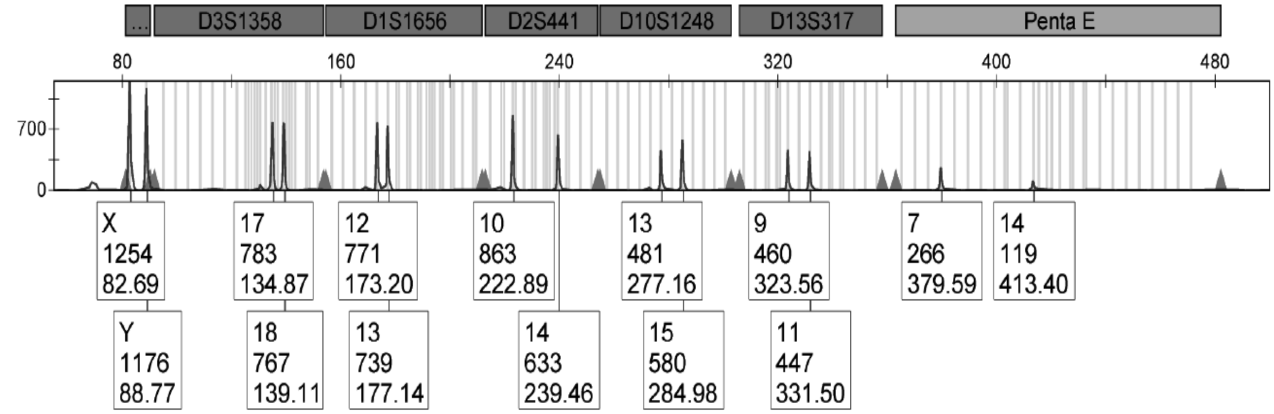

Figure 9B (continued)

JOE Channel

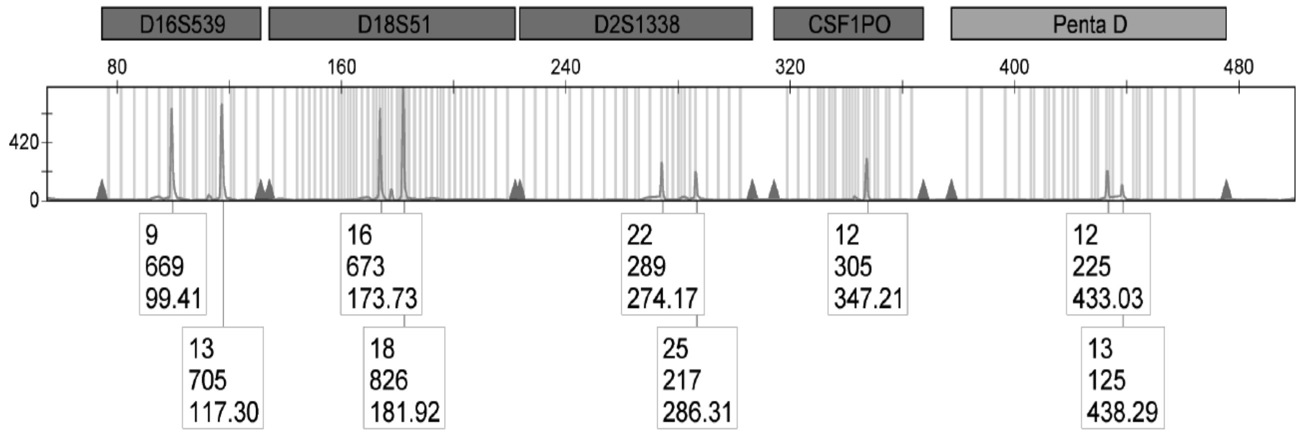

TMR Channel

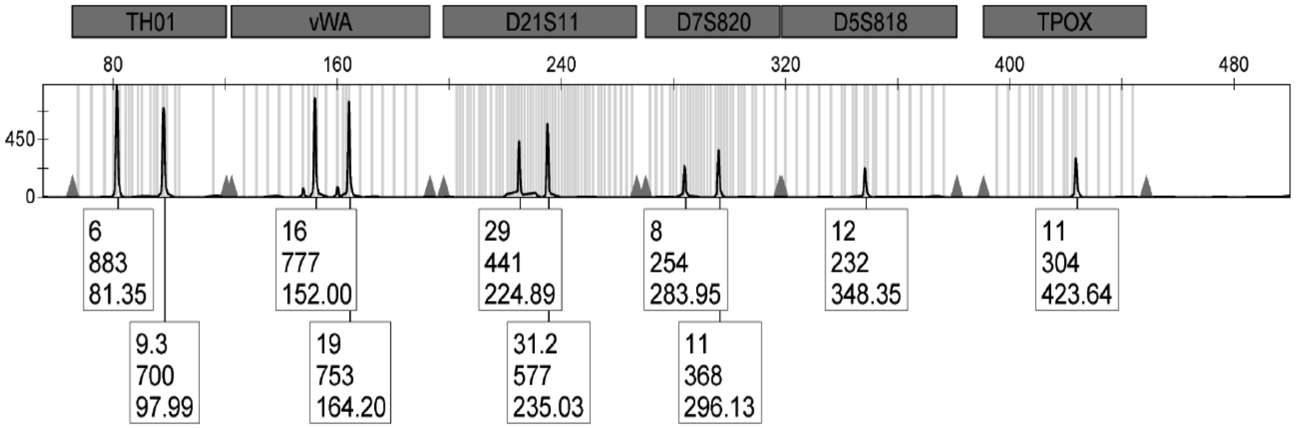

CXR Channel

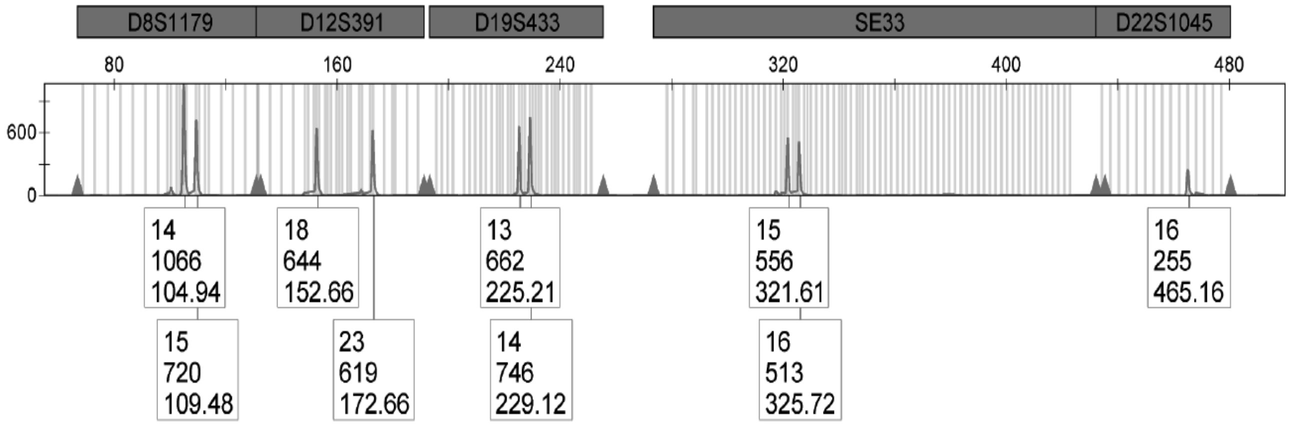

Figure 9B (continued)

TOM Channel

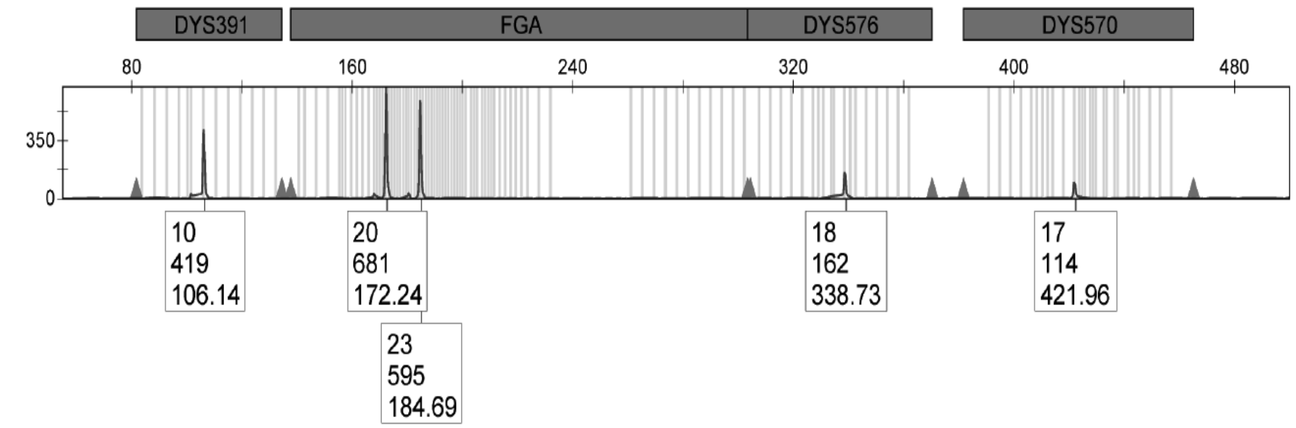

Chromatogram #3 PowerPlex® Fusion 6C (60 min)

FAM/Fluorescein Channel

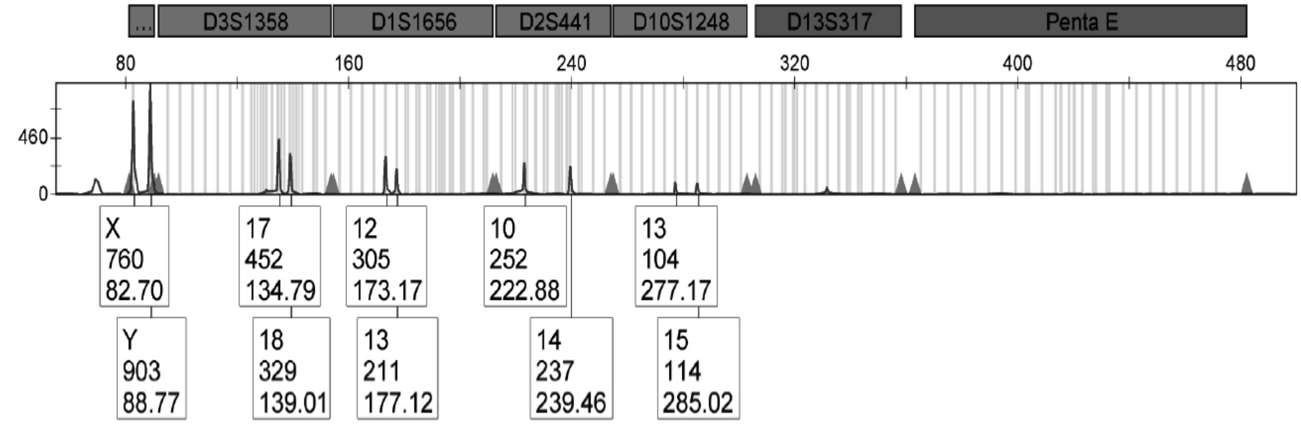

JOE Channel

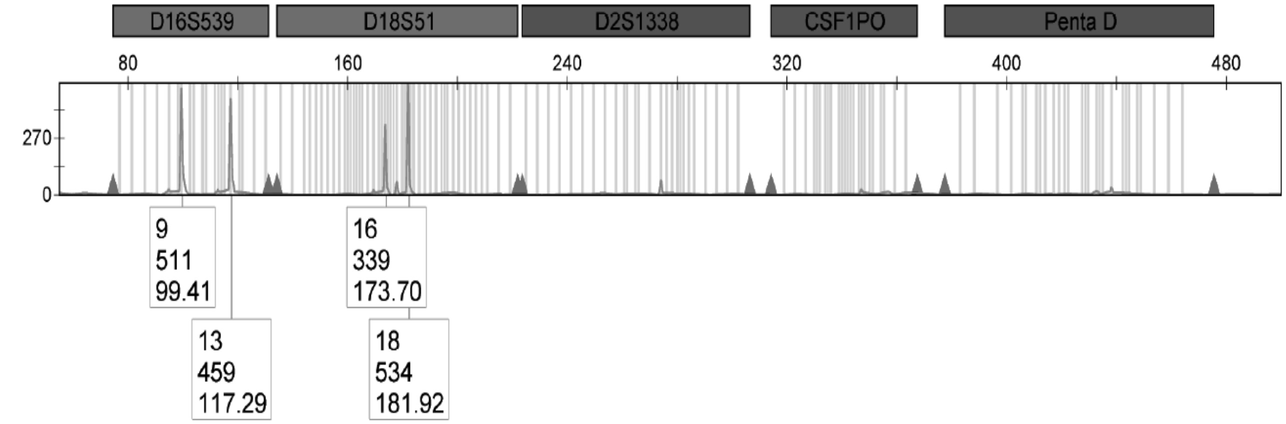

Figure 9B (continued)

TMR Channel

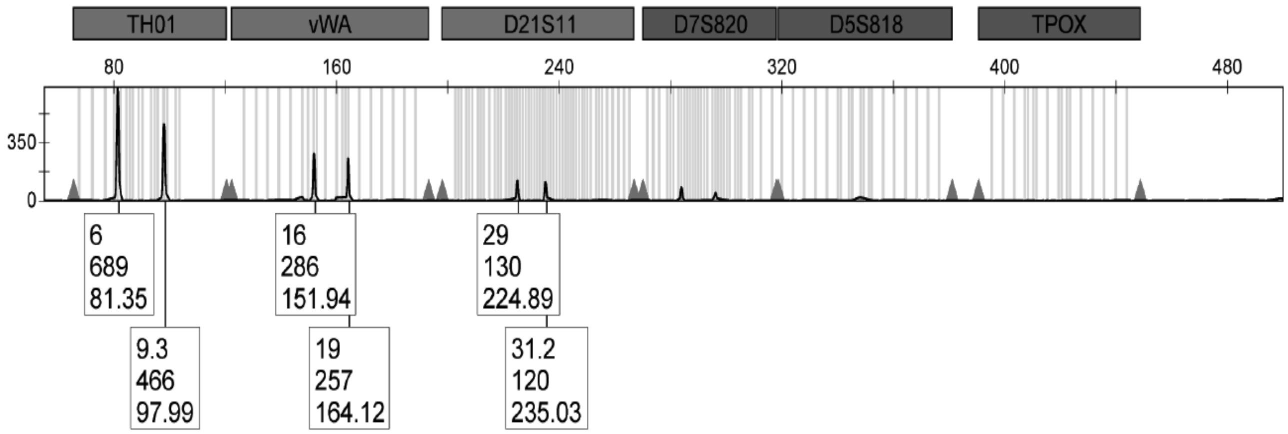

CXR Channel

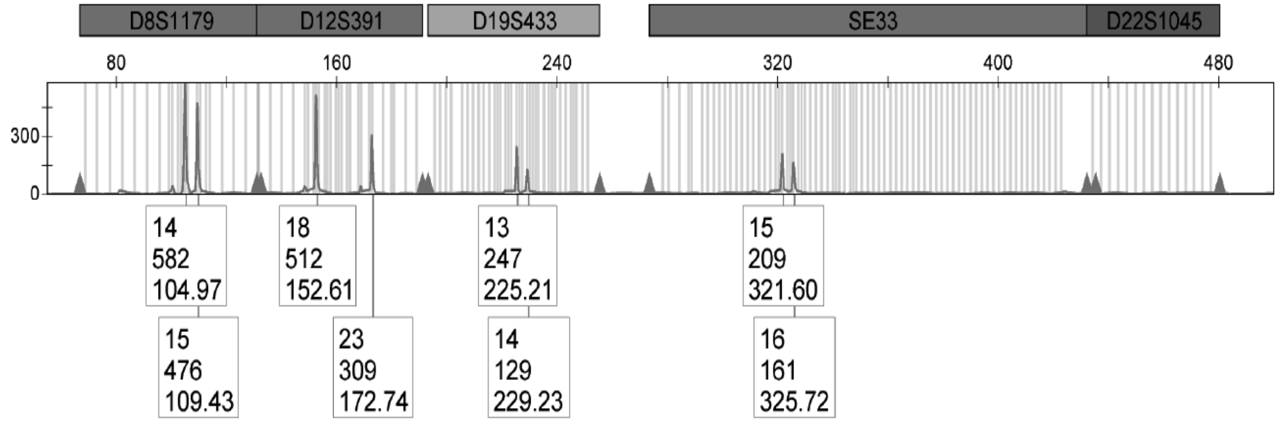

TOM Channel

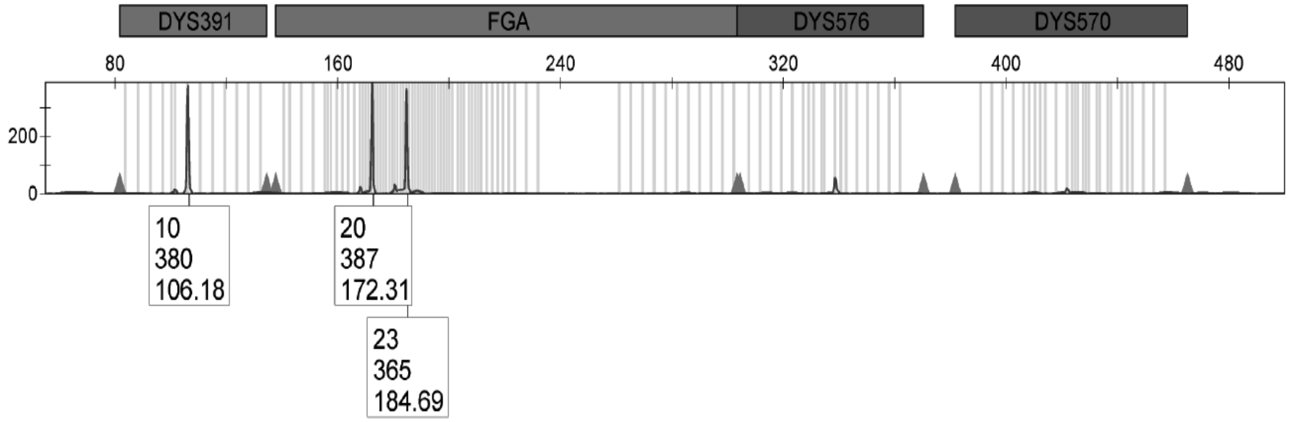

Figure 9B (continued)

Chromatogram #4 PowerPlex® Fusion 6C (NTC)

FAM/Fluorescein Channel

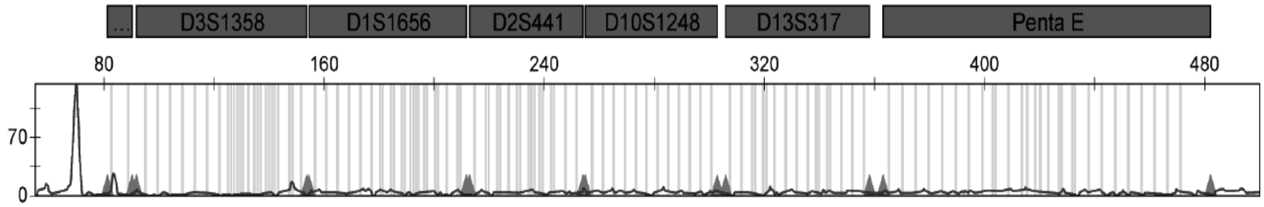

JOE Channel

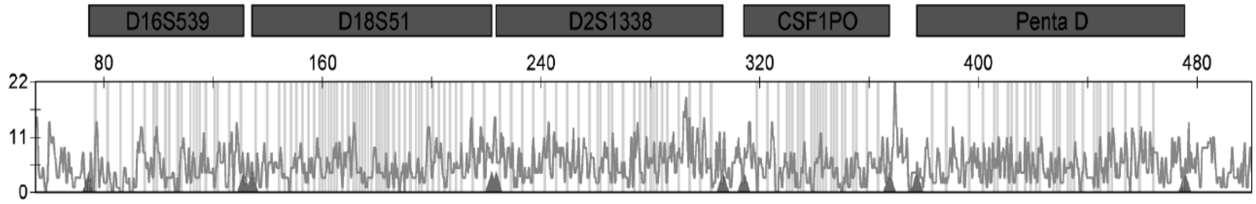

TMR_Channel

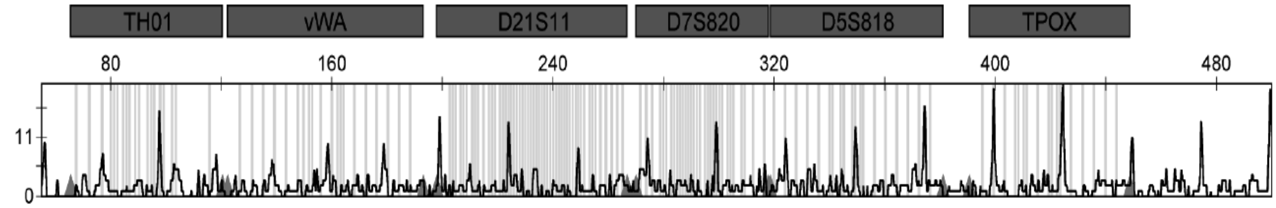

CXR Channel

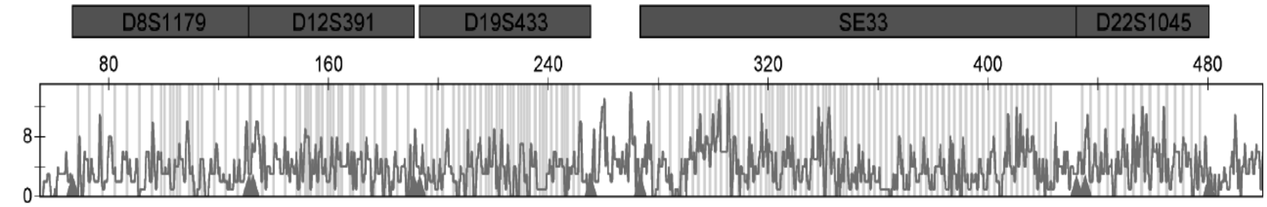

TOM Channel

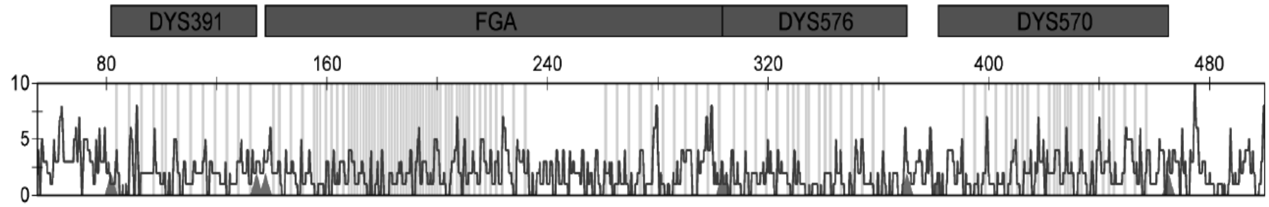

Figure 9B (continued)

Chromatogram #5 PowerPlex® Fusion 6C + CSFFW200 / CSFRV60J (0 min)

FAM/Fluorescein Channel

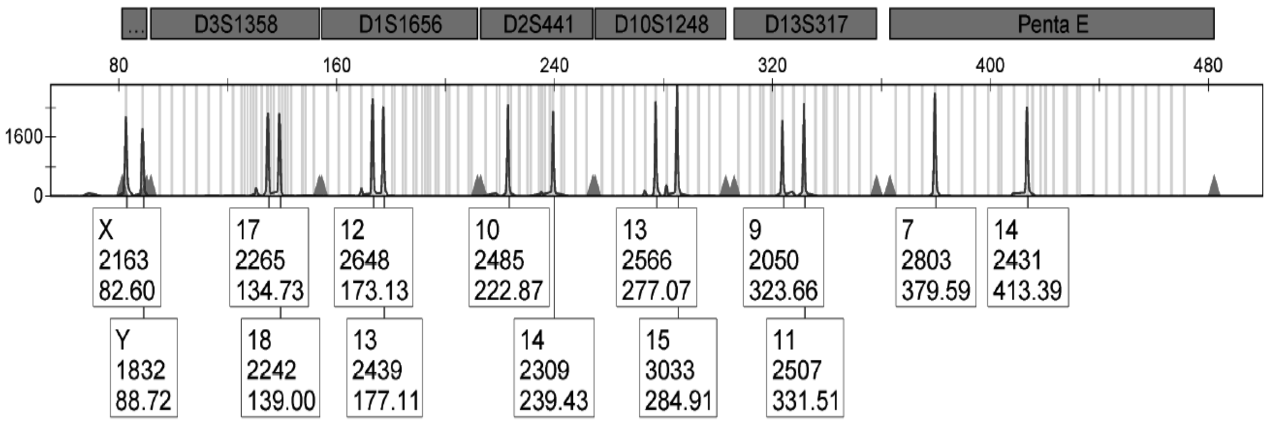

JOE Channel

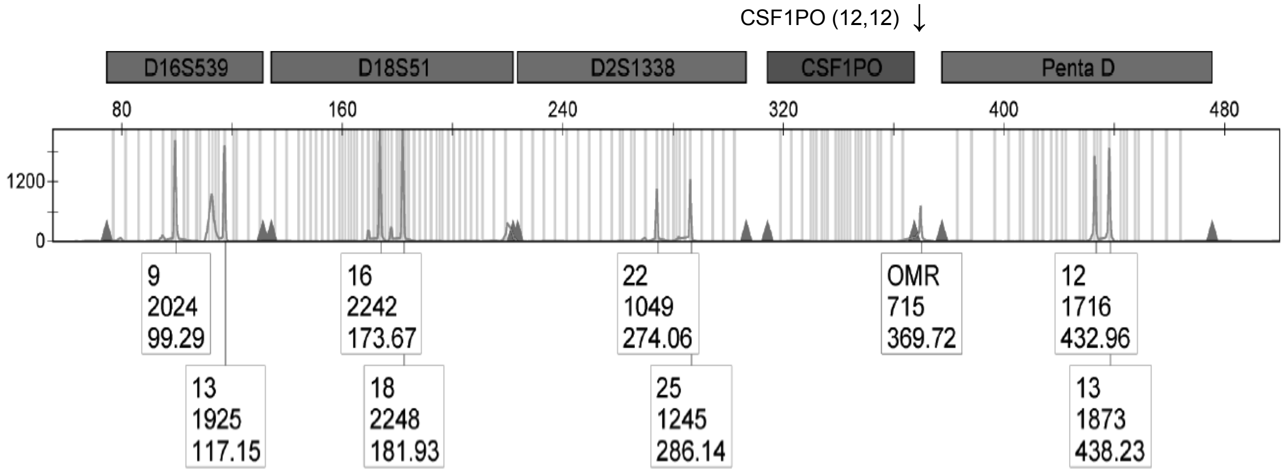

TMR Channel

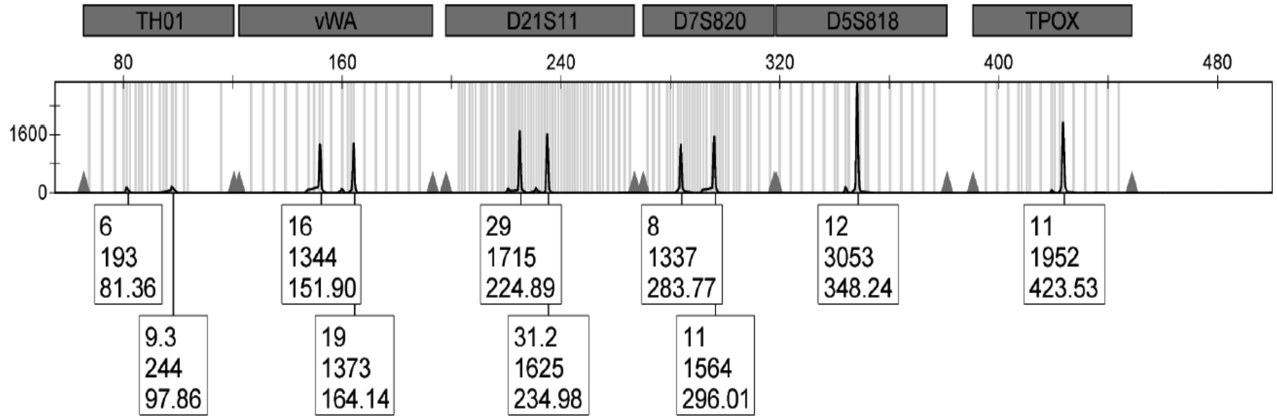

Figure 9B (continued)

CXR Channel

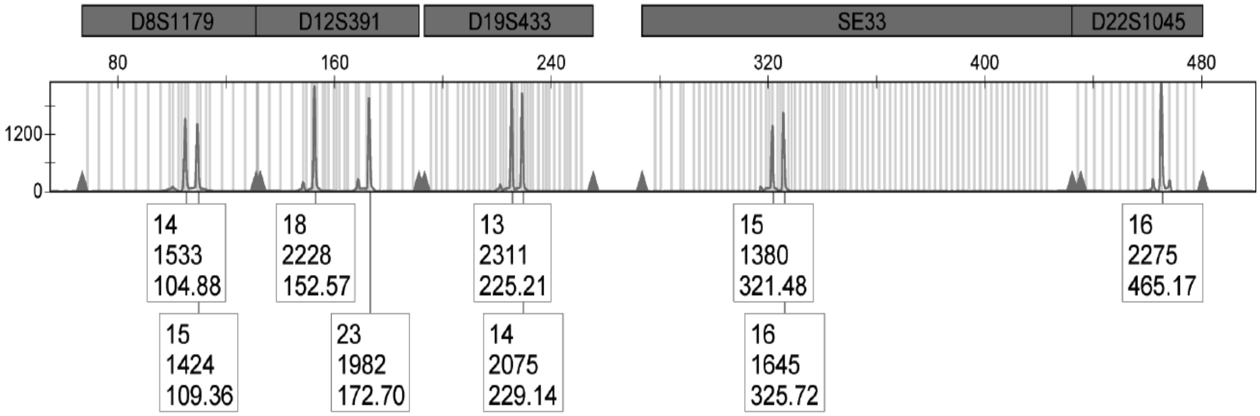

TOM Channel

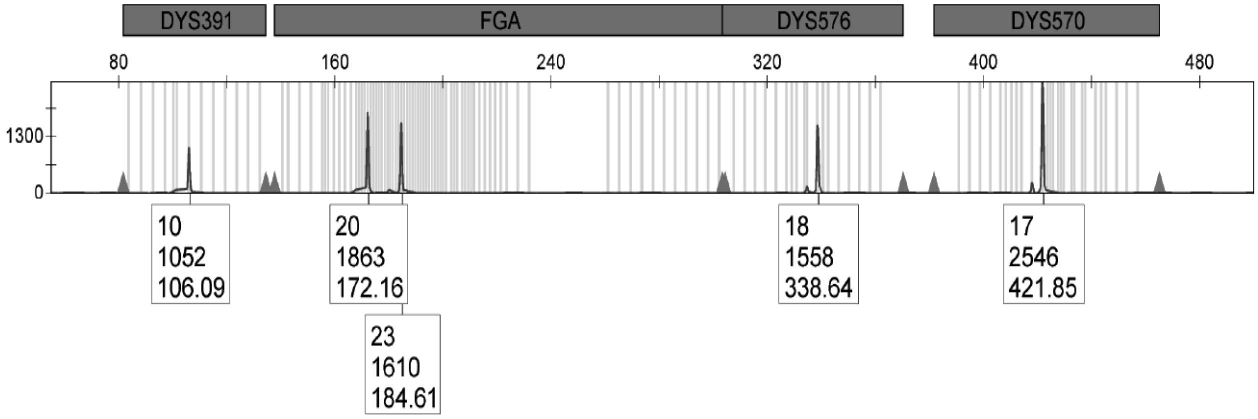

Chromatogram #6 PowerPlex® Fusion 6C + CSFFW200 / CSFRV60J (30 min)

FAM/Fluorescein Channel

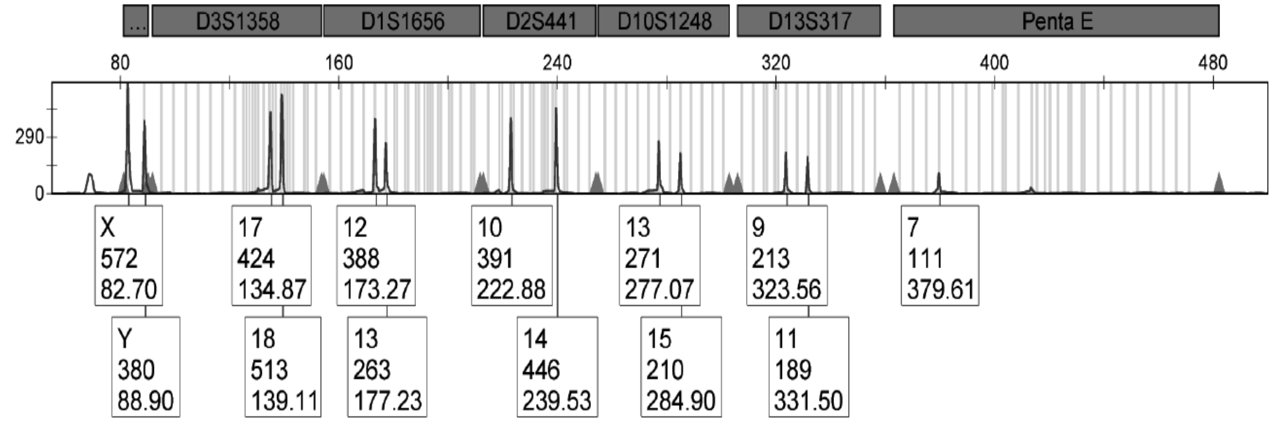

Figure 9B (continued)

JOE Channel

CSF1PO (12,12) ↓

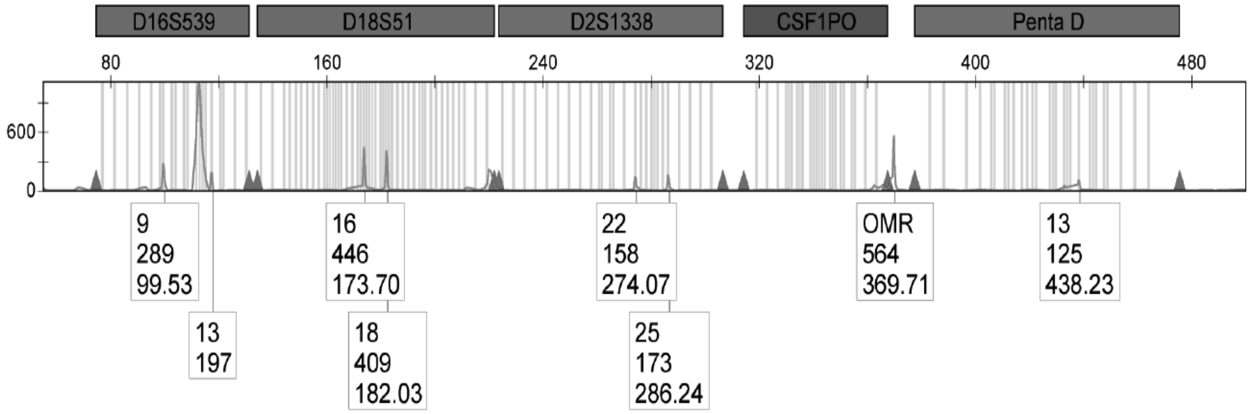

TMR Channel

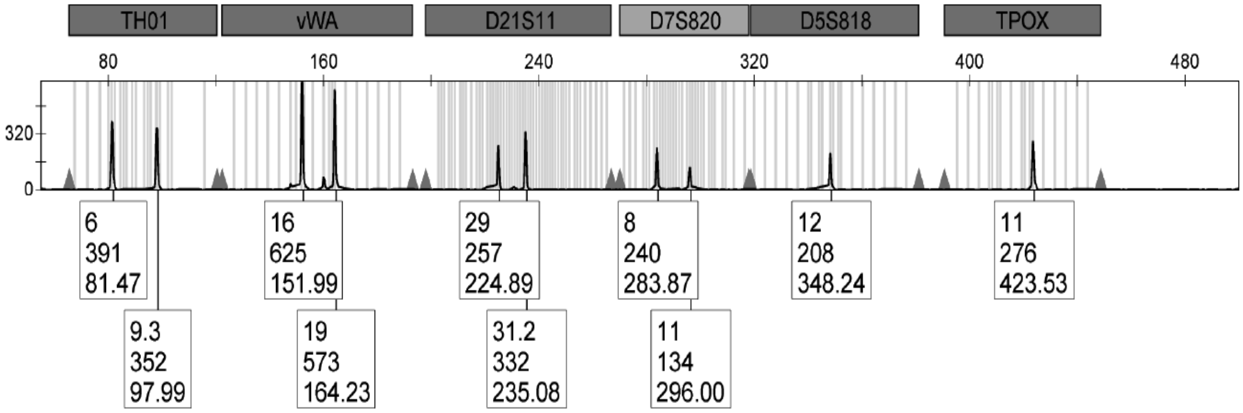

CXR Channel

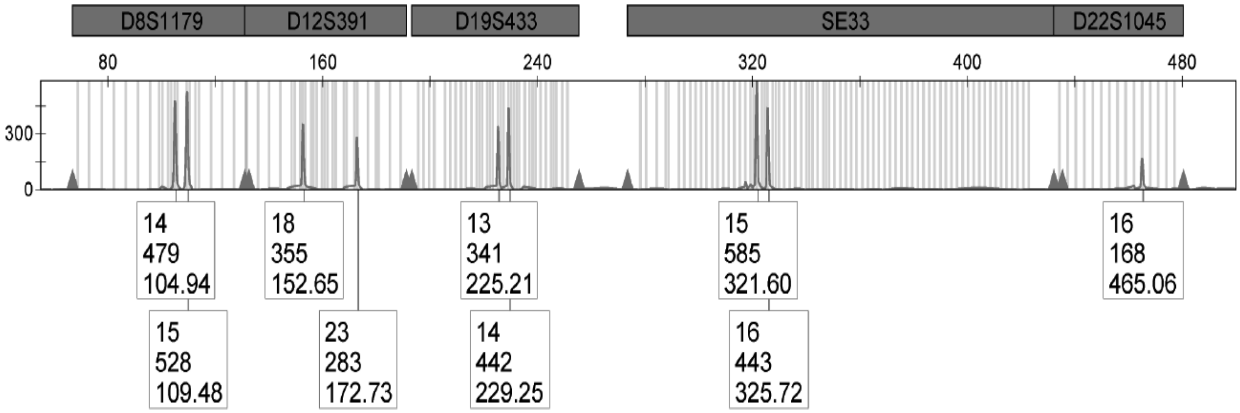

Figure 9B (continued)

TOM Channel

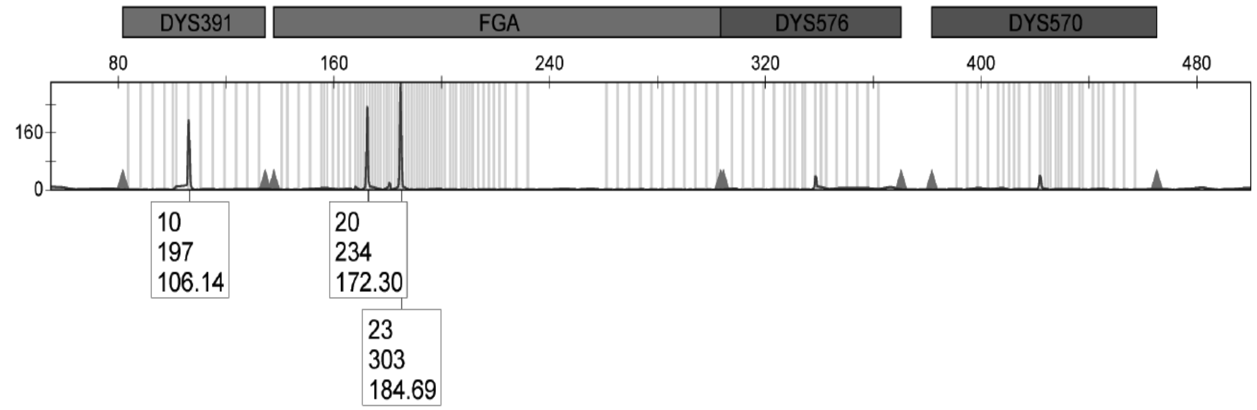

Chromatogram #7 PowerPlex® Fusion 6C + CSFFW200 / CSFRV60J (60 min)

FAM/Fluorescein Channel

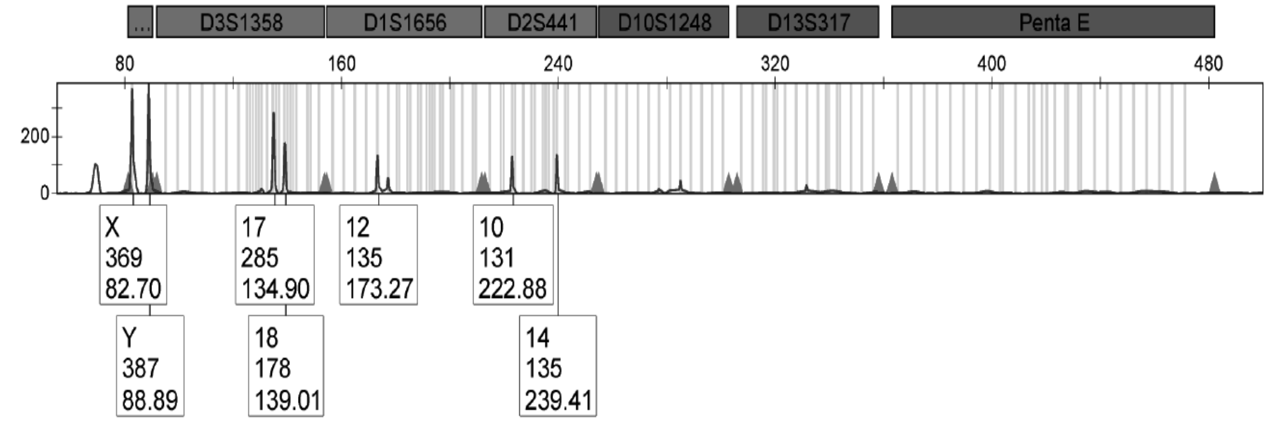

JOE Channel

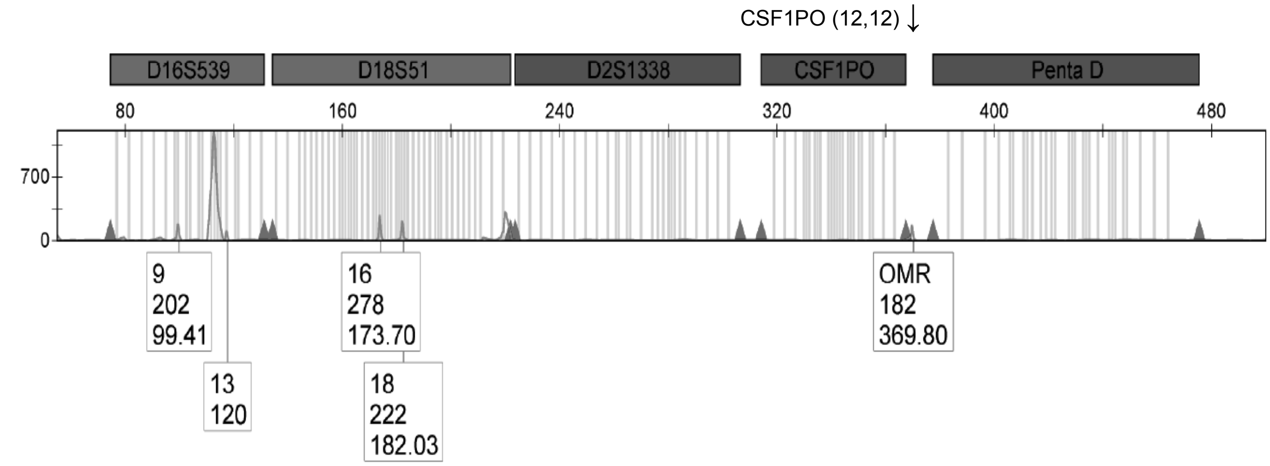

Figure 9B (continued)

TMR Channel

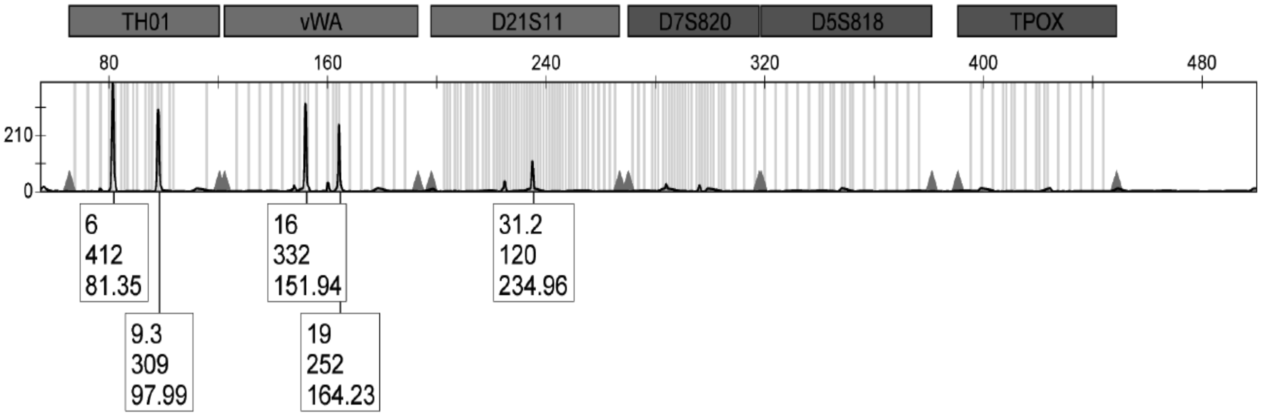

CXR Channel

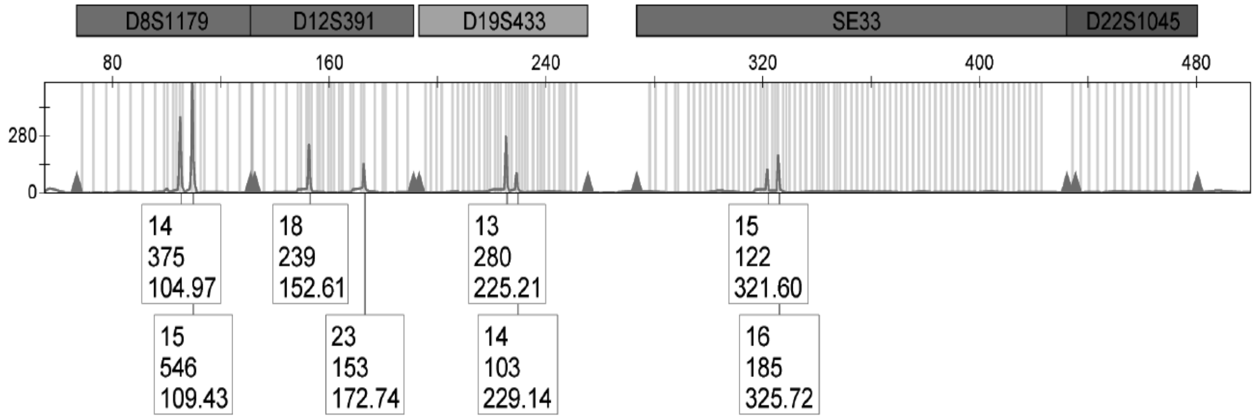

TOM Channel

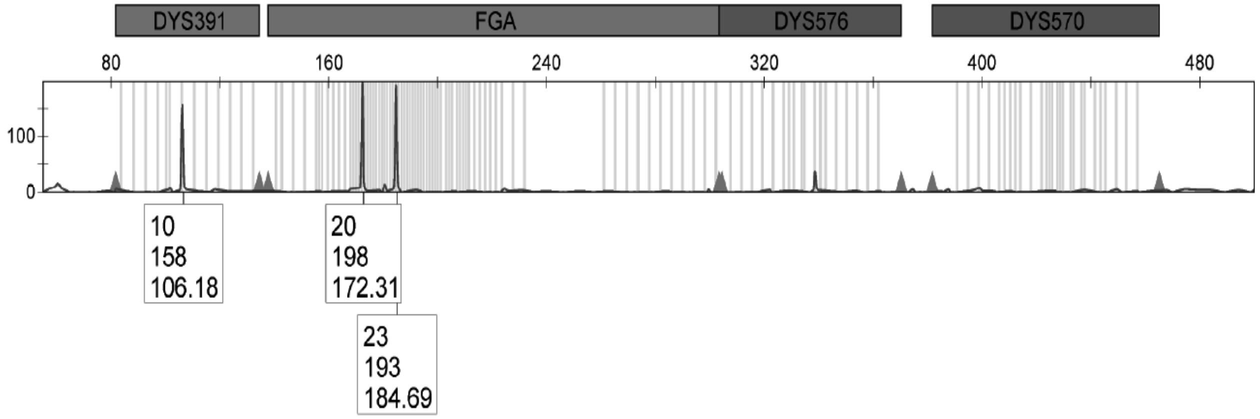

Figure 9B (continued)

Chromatogram #8 PowerPlex® Fusion 6C + CSFFW200 / CSFRV60J (NTC)

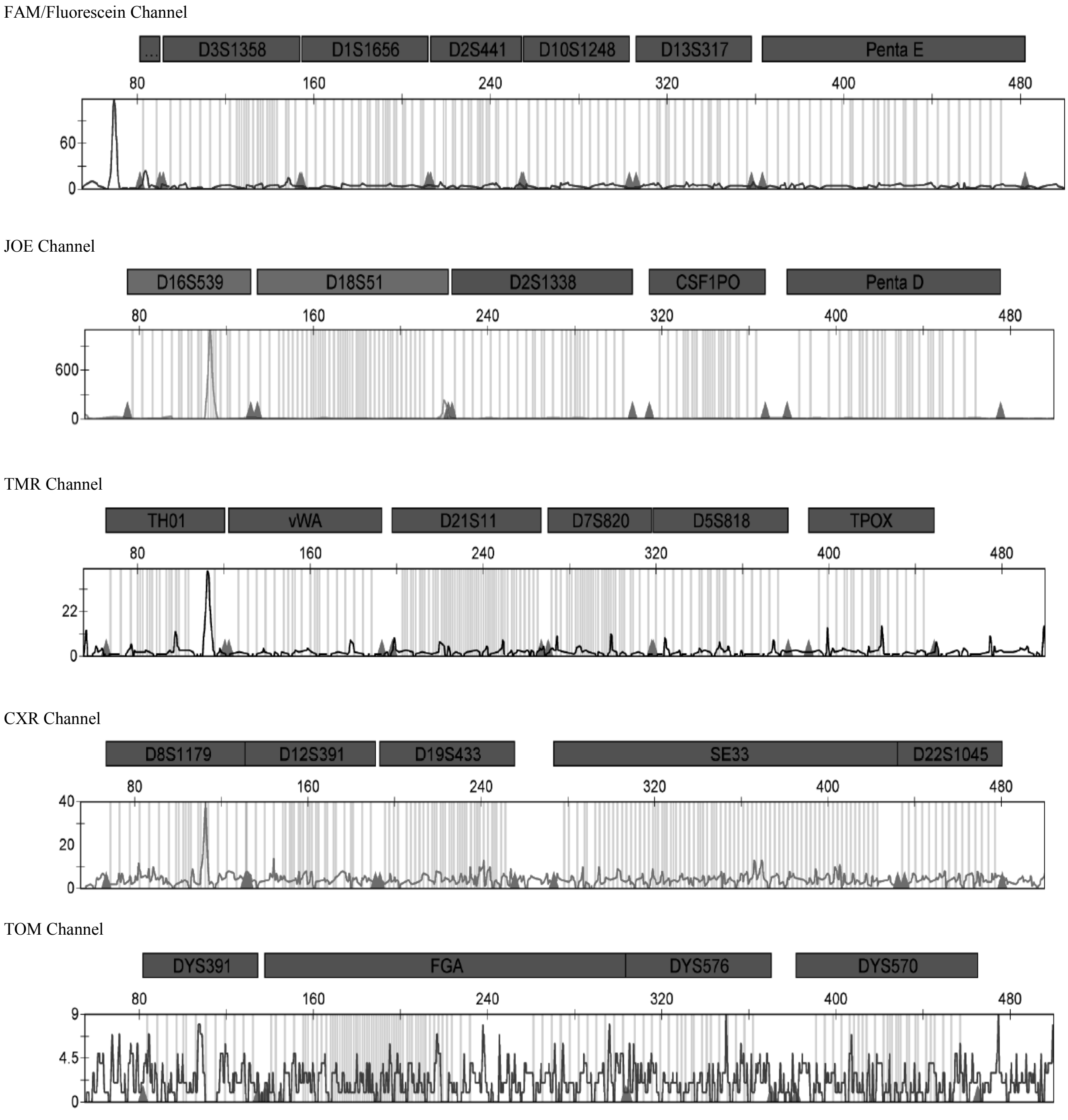

FAM/Fluorescein Channel
D3S1358
D1S1656
D2S441
D10S1248
D13S317
Penta E
JOE Channel
D16S539
D18S51
D2S1338
CSF1PO
Penta D
TMR Channel
TH01
vWA
D21S11
D7S820
D5S818
TPOX
CXR Channel
D8S1179
D12S391
D19S433
SE33
D22S1045
TOM Channel
DYS391
FGA
DYS576
DYS570

Figure 9B
