# Supplementary material for: Using long ssDNA polynucleotides to amplify STRs loci in degraded DNA samples
Source: PLoS One. 2017 Nov 3;12(11):e0187190. doi: 10.1371/journal.pone.0187190 (PMC5669423; doi:10.1371/journal.pone.0187190)
Supplement: S3 File — (PDF) [file pone.0187190.s003.pdf]

METHODS FOR USING LONG SSDNA POLYNUCLEOTIDES AS PRIMERS IN PCR ASSAYS

FIELD OF THE INVENTION

The invention relates to nucleic acid amplification reactions and assays involving the use of long ssDNA polynucleotides as primers for monoplex and multiplex polymerase chain reactions (PCR), and more particularly refers to methods to obtain a suitable length of a PCR product without the need to amplify longer off-target sequences, thereby allowing the amplification of short, fragmented and degraded nucleic acids.

BACKGROUND OF THE INVENTION

Detecting and identifying variations in DNA sequences among individuals and species has provided insights into evolutionary relationships, inherited disorders, acquired disorders and others aspects of molecular genetics and medicine.

In many instances these variations may involve different lengths of DNA, from several nucleotides down to just a single one. In particular, the detection of the variations in the number of short tandem repeats (STRs) is a challenging task aimed to provide new developments in the field of Molecular Biology, especially in the field of Genetic Identity. That is described in several publications: Litt and Luty (1989) *Am J. Hum Genet* 3(4):599-605; Tautz, D (1989) *NAR*17:6463-6471, Weber and May (1989) *Am J Hum Genet* 44:388-396; Edwards, A., *et al.* (1991) *Am. J. Hum. Genet.* 49: 746-756, Hammond, H. A., *et al.* (1994) *Am. J. Hum. Genet.* 55:175-189, Fregeau, C. J.; and Fourney, R. M. (1993) *BioTechniques* 15(1): 100-119; Schumm, J. W. *et al*. (1994) in *The Fourth International Symposium on Human Identification* 1993, pp.177-187, Edwards et al. (1991) *Nucleic Acids Res*. 19:4791; Chen *et al.* (1993) *Genomics* 15(3): 621-5, Harada *et al*. (1994) *Am. J. Hum. Genet*. 55: 175-189, Comings *et al.* (1995), *Genomics* 29(2):390-6, Utah Marker Development Group (1995), *Am. J. Genet*. 57:619-628, Jurka and Pethiyagoda (1995) *J. Mol. Evol.* 40:120-126), US 5,582,979, US 5,364,759, US 6,479,235 B1 and German Patent DE 38 34 636 C2.

The analysis of STRs variations is traditionally performed by PCR amplifying selected genetic markers from a DNA sample and then running the amplified products in a polyacrylamide gel electrophoresis (PAGE) or in a capillary electrophoresis (CE) column to determine the different lengths of the present alleles. The oligonucleotide primers used in the PCR amplification are usually fluorescently labelled with different dyes in order to allow the detection of the amplicons in CE

columns. The reaction is usually run in a multiplex format involving the simultaneous amplification and detection of several DNA targets.

A key aspect of the methods based upon DNA length is that the products from different markers must always have different sizes in order to prevent any overlapping during their detection. When using different fluorescent dyes the same concern applies to products labelled with the same dye. Since there is a limited number of fluorescent dyes that can be readily used and detected, the PCR primers in multiplex methods are designed to obtain several products of different, non-overlapping sizes for each fluorescent dye. However, this requires amplifying additional, longer off-target DNA regions in order to obtain the desired lengths, thereby compromising the amplification of short, fragmented and degraded DNA templates, as commonly found in DNA samples.

Several commercial and non-commercial reagents and kits are currently available for STRs applications in forensics, histocompatibility, kinship studies and DNA databases. A description of the primers and amplicons used in some of these and other systems is described in the NIST Short Tandem Repeat DNA Internet Database created by John M. Butler and Dennis J. Reeder (NIST Short Tandem Repeat DNA Internet Database created by John M. Butler and Dennis J. Reeder http://www.cstl.nist.gov/strbase/index.htm) while some other proprietary sequences are not disclosed. Nowadays most techniques used by primary manufacturers (Promega Corp., Madison, Wisconsin and Thermo Fisher Scientific Inc., Waltham, Massachusetts), rely on amplifying several longer off-target sequences in order to obtain an orderly distribution of the amplicons, usually within a range from 80 to 450 base pairs.

The degradation and fragmentation of DNA samples affect the integrity and sensitivity of biomolecular assays. The patent document US 2016/0168644A1 teaches methods for the quantitative analysis of such nucleic acid fragmentation/degradation and its “amplificability” by determining the ratio between the amount of long and short PCR amplification products for a given DNA sample.

The use of mini-STRs for reducing the extension of the DNA amplification, and thereby improving the PCR yield in degraded DNA samples, has been described by Whitaker JP *et al*. (Whitaker JP, Clayton TM, Urquhart AJ, *et al*. (*BioTechniques* (1995); 18:670–677)) and by Butler JM *et al.* (Butler JM, Shen Y, McCord BR. (*J Forensic Sci* (2003); 48). In this method the mini-STRs primers target regions adjacent to the repeats to reduce the extent of intact DNA necessary to amplify the desired repeated sequence. However, this always renders short DNA amplicons of approximately 100bp, thereby limiting the number of possible mini-STRs that can be used in multiplex assays due to their overlapping in electrophoresis gels or CE electropherograms.

Patent US 6,743,905B2 teaches methods for synthetizing and using mobility-modifying polymers linked to a sequence-specific nucleobase polymer. By incorporating organic chains into a primer the mobility-modifying polymers can provide larger DNA amplicons without the need to actually amplify the DNA equivalent to its length, thereby reducing the extent of the amplification. Since the mobility-modifying polymer is not amplifiable, to detect the increased amplicon size the polymer is usually linked to a detection molecule, e.g. a fluorescent dye, in order to detect the DNA strand where the mobility modifier is incorporated. This limits the length of this polymer to only short sizes to avoid the overlapping of the mobility-modified polymer primer with the labelled PCR products. Mobility modifiers are currently used for some genetic markers, e.g. CSF1PO (NIST Short Tandem Repeat DNA Internet Database created by John M. Butler and Dennis J. Reeder (http://www.cstl.nist.gov/strbase/index.htm), in the commercial kit Identifiler™, Thermo Fisher Scientific Inc.

Traditionally DNA oligonucleotide primers in the range of 18 to 25 nucleotides are used in PCR. In some applications longer primers can also be used for incorporating tags, adapters or other desired sequences to the resulting amplification product. In these cases usually a flanking, non-complementary sequence of approximately 10 to 20 nucleotides is added to the complementary sequence, resulting in primers of up to approximately 45 nucleotides long. For instance, patent document US 2013/0115605A1 describes a method to produce long DNA stretches by PCR using adapters shorter than 70 nucleotides and long extension primers of 70 nucleotides or more for the construction of genes. Trichas G. *et al* have used long reverse primers of 106 nucleotides in plasmid construction by amplifying a target sequence for bicistronic expression in transgenic mice. (Trichas G., Begbie J., Srnivas S., BCM Biology, (2008) 6:40). Also, long ssDNA polynucleotides – commercially named Ultramers™ by the DNA manufacturer IDT (Integrated DNA Technologies, Coralville, Iowa) – have been used in PCR, mostly as copy number standards in quantitative PCR (Viljoen C.D., Thompson G.G., Sreenivasan S., *Gene*, (2013) Mar 1;516(1):143-5), gene construction (Gibson D., *Nucleic Acids Res*, (2009) 37(20):6984–6990) and mutagenesis (Sabel, J., Mutagenesis Application Guide, (2011), Integrated DNA Technologies).

It would therefore be convenient to have a method to obtain a suitable length of a PCR product without the need to amplify longer off-target sequences, then focusing on amplifying only the specific and informative target sequences.

SUMMARY OF THE INVENTION

It is an object of the present invention to provide a use of long, single stranded DNA (ssDNA) polynucleotides as primers, instead of the usual shorter DNA oligonucleotides, yielding larger amplicons without the need to actually amplify extra DNA regions. In reducing the required template length between a given pair of primers, the performance of a PCR assay is greatly increased, particularly in degraded DNA samples, as previously shown by the use of mini-STRs.

It is another object of the present invention to provide a method to perform a polymerase chain reaction (PCR) comprising the following steps:

(a) Providing a nucleic acid sample;

(b) Hybridizing said nucleic acid sample to one or more pair of primers where at least one primer consists of a single stranded DNA polynucleotide having a length of 60 or more nucleotides;

(c) Subjecting said nucleic acid sample to a PCR, wherein the reaction mixture medium contains at least one of said primers; and

(d) Detecting the length of the amplified products.

It is still another object of the invention to provide a method to perform a polymerase chain reaction (PCR) wherein the target sequence can be amplified from any nucleic acid suitable for the PCR technique, for instance genomic DNA, mitochondrial DNA, plasmid DNA, viral DNA or RNA, circulating cell-free DNA, synthetic DNA and messenger RNA. The preferred samples to amplify are degraded or fragmented nucleic acids encoding genetic marker sequences. The method allows to obtain amplicons of a suitable size for their detection in the presence of a degraded or fragmented target sequence in the template. The fragmentation may have been caused by any biological, physical or chemical effect on these target sequences.

It is even another object of the invention to provide a method to perform a polymerase chain reaction (PCR) wherein at least one primer has a length between 60 and 300 nucleotides. It may be obvious for any person skilled in the art that primers longer than 300 nucleotides may also be used in the described method, these primers being accounted for in the method of invention. The long ssDNA primers may or may not be fully complementary to the target sequence, for instance while having a 3’ priming, complementary sequence to the target, they may have heterologous, non-complementary sequences at the 5’-end region. One or some of the primers present in the reaction may be labelled by several means, for instance by attaching fluorescent dyes, luminescent moieties, antigens, haptens or any other signaling tags.

It is still another object of the invention to provide a method to perform a polymerase chain reaction (PCR) wherein the method may involve the hybridization of several pair of primers that amplify several different target sequences of nucleic acids at the same time, where each target sequence is a genetic marker; and where the primers and the target sequences are in the same reaction mixture. Therefore, the method can be used in different PCR formats with a different number of pair of primers as in monoplex, duplex, triplex and multiplex assays.

It is another object of the invention to provide a method to detect a genetic marker in a degraded nucleic acid sample employing the polymerase chain reaction (PCR), comprising the following steps:

(a) Providing a nucleic acid sample that contains one or more genetic markers;

(b) Hybridizing said nucleic acid sample to one or more pair of primers where at least one primer consists of a single stranded DNA polynucleotide having a length of 60 or more nucleotides;

(c) Subjecting said nucleic acid sample to a PCR, wherein the reaction mixture medium contains at least one of said primers; and

(d) Detecting the length of the amplified products.

It is a further object of the invention to provide a PCR kit containing one or more pair of primers, where at least one of the primers has a length of 60 nucleotides or more.

It is also an object of the invention to provide a PCR kit including a DNA polymerase enzyme or enzymes, dNTPs (deoxyribonucleotides triphosphate), buffers, salts, control template, size standards and all other components required to amplify and detect PCR products.

BRIEF DESCRIPTION OF THE DRAWINGS

The subject matter regarded as the invention is particularly pointed out and distinctly claimed in the concluding portion of the specification. The invention, however, together with objects, features and advantages thereof, may be best understood by reference to the following drawings in which:

Figure 1 shows the location of the different primers on the DNA regions to be amplified. The sequence for each described primer is included in the listing of sequences.

Figure 2: Fig. 2A shows the amplification results on 2800M DNA (Promega) for *locus* CSF1PO in an agarose gel electrophoresis: Lane 1: 100bp Ladder (Promega); Lane 2: 345bp band with PowerPlex® 16HS primers CSFFW2416HSJ/CSFRV2216HS; Lane 3: NTC with PowerPlex® 16HS primers CSFFW2416HSJ/CSFRV2216HS; Lane 4: 370bp band with primers CSFFW2416HS/ CSFRV60J; Lane 5: NTC with primers CSFFW2416HS/CSFRV60J; Lane 6: 370bp band with primers CSFFW120/CSFRV60J; Lane 7: NTC with primers CSFFW120/CSFRV60J; Lane 8: 370bp band with primers CSFFW200/CSFRV60J; Lane 9: NTC with primers CSFFW200/CSFRV60J and Lane 10: 100bp Ladder (Promega). Figure 2B shows the diagram of CSF1PO amplification with primers CSFFW2416HSJ/CSFRV2216HS and CSFFW200 (200nt)/CSFRV60J (60nt).

Figure 3A shows the amplification results for *locus* CSF1PO with different DNA templates in a CE electropherogram. Electropherogram #1: 2800M DNA with primers CSFFW120/CSFRV60J, Electropherogram #2: 9947A DNA with primers CSFFW120/CSFRV60J, Electropherogram #3: 9948 DNA with primers CSFFW120/CSFRV60J, Electropherogram #4: K562 DNA with primers CSFFW120/CSFRV60J, Electropherogram #5: NTC control with primers CSFFW200/CSFRV60J, Electropherogram #6: 2800M DNA with primers CSFFW200/CSFRV60J, Electropherogram #7: 9947 DNA with primers CSFFW200/ CSFRV60J, Electropherogram #8: 9948 DNA with primers CSFFW200/CSFRV60J, Electropherogram #9: K562 DNA with primers CSFFW200/CSFRV60J and Electropherogram #10: NTC control with primers CSFFW200/CSFRV60J. B: Detail of CSF1PO triplet profile from Electropherograms #3 and #8.

Figure 4. Fig. 4A: Agarose gel electrophoresis for monoplex, duplex and triplex amplifications of markers CSF1PO, Penta E and DYS391 on 2800M DNA, wherein Lane 1 100 bp Ladder (Promega), Lane 2: CSF1PO monoplex with primers CSFFW120/CSFRV60J, Lane 3: Penta E monoplex with primers PEFW120/PERV60F, Lane 4: DYS391 monoplex with primers DYS391F2/DYSRV120, Lane 5: CSF1PO/Penta E duplex with primers CSFFW120/ CSFRV60J + PEFW120/PERV60F, Lane 6: CSF1PO/DYS391 duplex with primers CSFFW120/CSFRV60J + DYS391F2/DYSRV120, Lane 7: Penta E / DYS391 duplex with primers PEFW120/PERV60F + DYS391F2/DYSRV120, and Lane 8: CSF1PO/Penta E / DYS391 triplex with primers CSFFW120/CSFRV60J + PEFW120/PERV60F + DYS391F2/DYSRV120; Figure 4B: Primer design for amplification of CSF1PO, Penta E and DYS391.

Figure 5 shows a capillary electrophoresis electropherogram of the triplex PCR of CSF1PO (JOE channel), Penta E (FAM channel) and DYS391 (FAM channel) on 2800M DNA with primers CSFFW120/CSFRV60J, PEFW120/PERV60F and DYS391F2/DYSRV120.

Figure 6 shows the amplification results of *locus* DYS391 on 2800M DNA (Promega) in an agarose gel electrophoresis: Lane 1: 100bp Ladder (Promega), Lane 2: 148bp band with primers DYS391F2/DYSRV60, Lane 3: NTC with primers DYS391F2/DYSRV60, Lane 4: 208bp band with primers DYS391F2/DYSRV120, Lane 5: NTC with primers DYS391F2/DYSRV120, Lane 6: 288bp band with primers DYS391F2/DYSRV200, Lane 7: NTC with primers DYS391F2/DYSRV200, Lane 8: 148bp band with primers DYS391F2/DYSRV23+M13, Lane 9: NTC with primers DYS391F2/DYSRV23+M13, Lane 10: 288bp band with primers DYS391F2/DYSRV120+M13, Lane 11: NTC with primers DYS391F2/DYSRV120+M13 and Lane 12: 100bp Ladder (Promega).

Figure 7: Fig. 7A shows the layout of the DYS391 primers added to PowerPlex®21 (Promega). Figure 7B shows the capillary electrophoresis electropherogram for the DYS391 addition to PowerPlex®21, wherein Electropherogram #1: 2800M DNA without extra primers, Electropherogram #2: 2800M DNA with primers DYS391F2/DYSRV120 (FAM channel) and Electropherogram #3: 2800M DNA with primers DYS391F2/DYSRV200 (FAM channel).

Figure 8 shows the qPCR profile for *locus* CSF1PO on 2800M DNA (Promega) degraded by different times at 95ºC. Sample 1 (black, solid line): 0 minutes with primers CSFFW2416HSJ/CSFRV2216HS, Sample 2 (black, dashed line): 10 minutes with primers CSFFW2416HSJ/CSFRV2216HS, Sample 3 (black, dotted line): 30 minutes with primers CSFFW2416HSJ/CSFRV2216HS, Sample 4 (black, thin line): NTC with primers CSFFW2416HSJ/CSFRV2216HS, Sample 5 (gray, solid line): 0 minutes with primers CSFFW200/CSFRV60J, Sample 6 (gray, dashed line): 10 minutes with primers CSFFW200/CSFRV60J, Sample 7 (gray dotted line): 30 minutes with primers CSFFW200/CSFRV60J and Sample 8 (gray, thin line): NTC with primers CSFFW200/ CSFRV60J.

Figure 9: Fig. 9A shows the qPCR profiles of 2800M DNA (Promega) heated at 95ºC for 0, 10, 30, 45 and 60 minutes using the primer pair CSFFW200/CSFRV60J. Sample 1 (black, solid line): 0, Sample 2 (black, dashed line): 10 minutes, Sample 3 (black, dotted line): 30 minutes, Sample 4 (gray, solid line): 45, Sample 5 (gray, dashed line): 60 minutes, Sample 6 (gray, dotted line): NTC (no template control). Figure 9B: DNA samples heated for 0, 30 and 60 minutes were used –along an NTC (no template control) – to demonstrate the advantage of using *superprimers* in a current CODIS (Combined DNA Index System, FBI) commercial kit. To that aim 0.8 µM (final concentration) of each primer of the CSFFW200/CSFRV60J pair was added to the PowerPlex® Fusion 6C reaction mix, which already contains 54 primers that amplify 27 *loci*. The CE electropherogram shows that the peak signals decrease or disappear as the DNA is more degraded, especially for the longer amplicons, while

the peak corresponding to the CSFFW200/CSFRV60J pair is still visible in the JOE channel for the most degraded DNA.

Figure 10: Fig. 10A shows the amplification results in agarose gel electrophoresis of the very long primers CSFFW260 (260nt) and CSFFW300 (300nt) for *locus* CSF1PO on 2800M DNA (Promega): Lane 1: 100bp Ladder (Promega); Lane 2: 370bp band with primers CSFFW200/CSFRV60J; Lane 3: NTC with primers CSFFW200/CSFRV60J; Lane 4: 370bp band with primers CSFFW260/CSFRV60J; Lane 5: NTC with primers CSFFW260/CSFRV60J; Lane 6: 410bp band with primers CSFFW300/CSFRV60J; Lane 7 NTC with primers CSFFW300/CSFRV60J; Lane 8: 100bp Ladder (Promega). Figure 10B shows the layout for the CSF1PO amplification with primers CSFFW200 (200nt)/CSFRV60J (60nt), CSFFW260 (260nt)/CSFRV60J (60nt) and CSFFW300 (300nt)/CSFRV60J (60nt)

DETAILED DESCRIPTION OF THE INVENTION

A genetic marker is understood as a gene or DNA sequence with a known location on a genome that can be used to identify a region associated to a given trait or phenotype, to inherited diseases and to the identification of individuals and species. The term *locus* is sometimes used to express the same concept.

A target sequence refers to a segment of nucleic acid of interest that is selectively amplified using a sequence-specific primer.

A primer is an oligonucleotide or polynucleotide capable of hybridizing to a complimentary segment of nucleic acid to allow the initiation of the replication by a DNA polymerase enzyme.

For the purpose of the present description, the term *superprimer* refers to a single stranded DNA (ssDNA) polynucleotide primer of 60 nucleotides or longer, more preferably between 60 and 300 nucleotides.

The use of long, single stranded DNA (ssDNA) polynucleotides as primers (*superprimers),* instead of the usual shorter DNA oligonucleotides, yields larger amplicons without the need to actually amplify extra DNA regions. In reducing the required template length between a pair of primers, the performance of the PCR assays is greatly increased in degraded DNA samples.

Single stranded DNA polynucleotides ranging from sixty to hundreds of nucleotides, far longer than the smaller oligonucleotides containing few nucleotides - as normally used as primers in PCR - can optimally be employed as surrogate primers without any particular concerns on their annealing temperatures or the need of special PCR conditions. While we preferred a hot start PCR option, non-hot start PCR may also be utilized. The use of long ssDNA primers allows to obtain large DNA amplicons without the need to actually amplify a large portion of DNA template.

The goal of the present invention is to increase the sensitivity of PCR assays by reducing the required length of available intact DNA template needed for its amplification, while at the same time obtaining larger DNA amplicons suitable for size discrimination in analysis by CE and other related methods. For that aim, the addition of long ssDNA polynucleotides as primers or *superprimers* allows the polymerization of specific, small DNA segments while at the same time yielding product sizes suitable for their convenient detection.

The inventive methods are based upon the decreasing efficiency of the extension by polymerase enzymes in the presence of degraded DNA as the PCR pair of primers are farther apart. The method of the invention is useful for detecting genotypes in degraded or fragmented DNA samples as is typical in forensic casework and aged samples.

The method demonstrated that long ssDNA primers can be readily used for PCR assays in monoplex or multiplex formats. While we preferred a hot start PCR, a non-hot start enzyme may also be used. Actually no special PCR conditions or primer design were needed towards that goal other than the use of highly purified, long ssDNA polynucleotides (*superprimers*) as priming reagents. Moreover, we have also showed that these primers can be applied in the amplification of degraded DNA templates with the same beneficial effect of the small mini-STRs, while at the same time producing larger DNA amplicons of flexible lengths that are more suitable for size discrimination assays.

Primers totally complementary to the template sequence or complementary only at their 3’ priming region were both successfully amplified. The use of non-homologous sequences may be important for providing flexibility in designing multiplex reactions with several primers. Furthermore, primers bearing long 5’, non-homologous sequences might be considered for performing mutagenesis and also for annealing, molecular binding and labeling strategies.

The invention method is suitable for PCR assays and for detecting variations that involve sequence and length polymorphisms. It may be applied to the current methods in use that rely on different

amplification lengths to distinguish variations in nucleic acid sequences. It may also be useful in the design of multiplex kits for the identification of individuals and species and for medical diagnostics.

The invention method increases the sensitivity of PCR assays by reducing the required length of available intact DNA template needed for the amplification, while at the same time obtaining larger DNA amplicons of flexible length suitable for size discrimination in analysis by CE and other related methods. For that aim, the use of long ssDNA polynucleotides as primers (*superprimers)* allows the enzymatic polymerization of specific, small DNA target regions while at the same time yielding product sizes suitable for their convenient detection.

The present invention increases the sensitivity of PCR assays by reducing the required length of the intact DNA template, while at the same time obtaining larger DNA amplicons suitable for size discrimination.

The method of the invention is useful to obtain a suitable length of a PCR product without the need to amplify off-target sequences towards that aim, thereby increasing the sensitivity of the assay for short, fragmented or degraded DNA samples by reducing the number of nucleotides being amplified. Additionally, more versatility is provided in the design of PCR assays by allowing the use of primers with large non-homologous sequences.

The design of PCR primers was based on the information provided by the NIST Short Tandem Repeat DNA Internet Database created by John M. Butler and Dennis J. Reeder (NIST Short Tandem Repeat DNA Internet Database created by John M. Butler and Dennis J. Reeder http://www.cstl.nist.gov/strbase/index.htm) and the published genome sequence from the GenBank (GenBank http://www.ncbi.nlm.nih.gov/genbank/).

All DNA primers were synthesized by Integrated DNA Technologies Inc. (IDT). Primers labelled with the fluorescent dyes 6-FAM (fluorescein) and JOE (6-carboxy-4',5'-dichloro-2',7'-dimethoxyfluorescein, NHS ester) were purified by HPLC. Primers of 120 and 200 nucleotides (Ultramers™) were PAGE purified. Primers of 260 and 300 nucleotides (Megamers™) were clonally purified. All other primers were desalted.

Figure 1 shows the location of the different primers on the amplified DNA regions used in the examples.

The primer sequences are:

CSFRV2216HS: SEQ ID N° 1

CSFFW2416HS: SEQ ID N° 2

CSFFW2416HSJ: SEQ ID N° 3. (It is identical to SEQ ID Nº2, but has a fluorescent JOE dye at the 5' end).

CSFFW120: SEQ ID N° 4

CSFFW200: SEQ ID N° 5

CSFRV60J: SEQ ID N° 6. (It may have a fluorescent JOE dye at the 5' end).

PEFW120: SEQ ID N° 7

PERV60F: SEQ ID N° 8. (It may have a fluorescent 6-FAM dye at the 5' end).

DYS391F2: SEQ ID N°9 (It may have a fluorescent 6-FAM dye at the 5' end).

DYSRV60: SEQ ID N° 10

DYS391RV120: SEQ ID N° 11.

DYS391RV200: SEQ ID N° 12.

DYSRV120+M13: SEQ ID N° 13.

DYSRV23+M13: SEQ ID N° 14.

CSFFW260: SEQ ID N° 15.

CSFFW300: SEQ ID N° 16.

Long ssDNA primers produced neat PCR products in a similar fashion to shorter oligonucleotide primers, without any artifacts or spurious bands. Figure 2A shows the PCR amplification of a 370bp region of the genotyping *locus* CSF1PO (5q33.1; human c-fms proto-oncogene for CSF-1 receptor gene, 6th intron GenBank Accession X14720) using ssDNA polynucleotides of 120 nucleotides (CSFFW120) or 200 nucleotides (CSFFW200) as forward primers and a 60-nucleotide primer (CSFRV60J) as reverse primer. A third forward primer of 24 nucleotides (CSFFW2416HS) with the same sequence as in the commercial kit PowerPlex®16HS (Promega) was also used.

Since the three forward primers share the same 5' sequence, they all yield the same 370bp product. A control amplification of 345bp with PowerPlex®16HS (Promega) primers CSFFW2416HSJ/CSFRV2216HS was also performed. Standard DNA 2800M (Promega) was used as template in all reactions.

Due to the greater length of the long ssDNA primers, only 62 nucleotides of non-repeat sequences span between the 3' ends of the primer pair CSFFW200/CSFRV60J. This contrasts with the 251-nucleotide distance in PowerPlex®16HS, even though the latter amplicon is 25 bases shorter (Fig. 2B).

The quality of the DNA profiles generated by long ssDNA primers was verified for different genotypes in reference cell lines. The CSF1PO region of DNA templates 2800M (genotype 12, 12), 9947A (genotype 10, 12), 9948 (genotype 10, 11, 12) and K562 (genotype 9, 10) was amplified with forward primers CSFFW120 (120nt) or CSFFW200 (200nt) and reverse primer CSFRV60J (60nt, JOE-labelled) and then analyzed by capillary electrophoresis (Fig. 3A).

All profiles agreed with the ones described in the literature for these cell types (James, R. New Control DNA for PowerPlex® Systems. Promega Corporation Web site. http://worldwide.promega.com/resources/profiles-in-dna/2011/new-control-dna-for-powerplex-systems/ Updated 2011; National Institute of Standards & Technology Certificate of Analysis Standard Reference Material® 2391b PCR-Based DNA Profiling Standard and America Type Culture Collection: ATCC STR Data for Human Cell Lines www.atcc.org/STR%20Database.aspx). Moreover, they showed the characteristic heterozygous imbalance present in cell lines K562 (dominant 10) and 9948 (dominant 10). There is also a small peak in cell line 9948 (Fig. 3B) that may represent the allele 12 triplet of CSF1PO which has a relative intensity less than 10 % of the dominant allele 10 (America Type Culture Collection: ATCC STR Data for Human Cell Lines www.atcc.org/STR%20Database.aspx and Forensic DNA Typing, Biology, Technology and Genetics of STR Markers, 2nd edition (2005), John, M. Butler, Elsevier Academic Press).

Other commonly used STR markers, *e.g.* DYS391 and Penta E, can be successfully amplified and combined in multiplex formats using long ssDNA primers (*superprimers*). Fig. 4A shows the amplification products for *loci* CSF1PO, Penta E and DYS391 on 2800M DNA using the following sets of primers pairs: CSFFW120/CSFRV60J, PEFW120/PERV60F and DYS391F2/DYSRV120 (Fig. 4B). These primer pairs were in turn successfully combined to produce all possible duplex combinations and a triplex. Figure 5 shows the CE profile of the triplex assay with the expected peak profiles for DYS391 and Penta E (FAM channel) and CSF1PO (JOE channel).

Long ssDNA primers (*superprimers*) with partial, non-complementary sequences may also be successfully utilized. Fig. 6 shows the amplification of the DYS391 STR region with forward primer DYS391F2 and reverse primers of 60 nucleotides (DYSRV60), 120 nucleotides (DYSRV120) or 200 nucleotides (DYSRV200). These three reverse primers share the same 3’-sequence and are fully complementary to the DYS391 region. In order to verify that non-complementary sequences may also be successfully utilized, we included reverse primers DYSRV23+M13 (60nt) and DYSRV120+M13 (200nt), both of them sharing the same homologous 3’ priming sequence with the other reverse primers, but having a non-complementary M13 phage sequence tag at their 5’-end region.

Primers fully complementary to the template as well as primers complementary only in the 3’ priming region were both successfully amplified in the STR DYS391 region yielding products of the expected size. The use of primers with non-complementary sequences in the 5’ region - which does not require annealing to the template for priming - might be important to provide flexibility in designing multiplex reactions with several primers by allowing the use of any sequence of choice. Primers with long 5’, non-homologous sequences might be useful for mutagenesis, gene building and other applications.

As shown, *superprimers* not fully complementary to the target sequence in their 5’ ends can be readily used, as long as their 3’-end sequences show homology to the priming region.

Long ssDNA primers (*superprimers*) are compatible with more complex PCR multiplex assays. In order to know if the long ssDNA fragments (*superprimers*) might somehow interfere in assays involving several primers, we added primer pairs DYS391F2/DYSRV120 and DYS391F2/DYSRV200 (Fig. 7A) into a commercial kit already containing 42 primers (PowerPlex® 21, Promega, with 42 primers for amplifying 21 *loci*). Figure 7B shows that the 2800M DNA profile for all 21 markers is not altered by the addition of the long primers, while a peak corresponding to DYS391 is identified in the FAM channel at the expected size for both pairs. *Superprimers* added to an existing kit provide the expected results without interfering in the multiplex detection of several *loci*. The method of the invention may be utilized in complex multiplex PCR assays.

Long ssDNA primers (*superprimers*) may be useful for amplifying degraded DNA. To demonstrate the advantage of using long primers on fragmented or degraded DNA samples, genomic DNA was subjected to a controlled degradation by heating at 95ºC at increasing periods of time. The resulting degraded DNA was qPCR amplified using either standard PowerPlex®16HS primers or the longer ssDNA pair of primers (*superprimers*) CSFFW200 (200nt)/CSFRV60J (60nt) (Fig. 2B). Figure 8 shows that for more degraded DNA the Ct (cycle threshold) in the reaction with long ssDNA primers becomes lower than in the one with the standard PowerPlex®16HS primers, illustrating that they are more suitable for amplifying degraded DNA.

Long ssDNA primers are helpful to detect genetic markers in complex multiplex assays involving dozens of primers when the DNA sample is degraded. For instance, *superprimer*s can be used in the current kits containing the CODIS (Combined DNA Index System) markers of the FBI (Federal Bureau of Investigation) database in order to amplify *loci* that otherwise might remain undetected, especially those ones that have large amplicon sizes. Figure 9A shows the qPCR profiles of 2800M DNA heated at 95ºC for increasing periods of time and then amplified using primers CSFFW200 (200nt) and JOE-labelled CSFRV60J (60nt). In spite of that the DNA gets more degraded by longer

heating incubations, the primer pair CSFFW200/CSFRV60J yields a PCR product in all time points. Degraded DNA was used –along an NTC (no template control) – to demonstrate the advantage of using *superprimers* in a current CODIS (Combined DNA Index System, FBI) commercial kit. To that aim the CSFFW200/CSFRV60J pair was added to the PowerPlex® Fusion 6C reaction mix, which already contains 54 primers that amplify 27 *loci*. The peak signals decrease as the DNA is more degraded, especially for the longer amplicons (Fig. 9B). On the contrary, degraded samples amplified with long ssDNA primers CSFFW200/CSFRV60J still show useful genotyping peaks for the CSF1PO marker in the JOE channel. This demonstrates the advantage of using *superprimers* when performing complex CODIS multiplex assays in the presence of degraded DNA samples.

As the DNA becomes more degraded the longer primers are more efficient in amplifying the resulting fragmented DNA templates due to the shorter amplification span between the 3' end of the forward and reverse primers. This is the same effect as seen in the mini-STRs (Advanced Topics in Forensic DNA Typing: Methodology, 1st edition (2011), John, M. Butler, Elsevier Academic Press), but with the additional benefit of yielding PCR products larger and of more flexible lengths that are suitable for multiplex assays.

While we have utilized long primers of up to 300 nucleotides, it is clear that even longer primers may be successfully used in the described method. This may be inferred by the negligibly difference in product yield between the primers of 120 and 200 nucleotides, as shown in the amplification of *locus* DYS391 (Fig. 7B). The peak area for DYSRV200 (200nt) in the CE electropherogram is only 20% smaller than the one for DYSRV120 (120nt). The locus CSF1PO was also readily amplified utilizing primers of 120, 200, 260 and 300 nucleotides (Fig. 10A). Then, it may be safely extrapolated that primers longer than 300 nucleotides may also be used for PCR without a significant detriment in the final yield.

The present invention is best illustrated by the following examples, which by no means can be interpreted as a limitation to its scope. On the contrary, it must be clearly understood that other versions, modifications and applications could be suggested to anyone skilled in the art after reading the present description, without departing from the spirit of the present invention and the reach of the included claims.

EXAMPLE 1

End-point PCR Using Long ssDNA Primers

A PCR amplification of a 370bp region of the genotyping *locus* CSF1PO was performed using ssDNA polynucleotides of 120 nucleotides (CSFFW120) or 200 nucleotides (CSFFW200) as forward primers

and a 60-nucleotide primer (CSFRV60J) as reverse primer. A third forward primer of 24 nucleotides (CSFFW2416HS) with the same sequence as in the commercial kit PowerPlex®16HS (Promega) was also used. A control amplification of 345bp with PowerPlex®16HS primers CSFFW2416HSJ / CSFRV2216HS was also included (Fig. 1). Standard DNA 2800M (Promega) was used as template in all reactions

End-point PCR amplifications were run in a MultiGene™ Gradient thermal cycler (Labnet) in a 20μL reaction volume containing 1X Colorless GoTaq® reaction buffer including 1.5mM $MgCl_2$ (Promega), 200uM each deoxyribonucleotide triphosphate, (Promega), 1 U GoTaq® Hot Start DNA Polymerase (Promega), 500nM of each primer (IDT) and 500pg of genomic DNA standard 2800M (Promega). Cycling conditions encompassed an initial denaturation step of 2 min at 94ºC, followed by 35 cycles of 10 seconds at 94ºC, 60 seconds at 59ºC and 45 seconds at 72ºC and a final incubation of 10 minutes at 60ºC. PCR products were electrophoresed through a 2% agarose gel in 1X TBE Buffer at 100 Volts constant voltage and visualized with ethidium bromide. 3μL of sample or DNA size standard (100bp Ladder, Promega) were loaded per well.

The gel electrophoresis shows that long ssDNA primers produced neat PCR products in similar fashion to shorter oligonucleotide primers, without any artifacts or spurious bands (Fig. 2A).

EXAMPLE 2

Capillary Electrophoresis (CE) Profiles of Different DNA Genotypes Using Long ssDNA Primers

The CSF1PO region of DNA templates 2800M (genotype 12, 12), 9947A (genotype 10, 12), 9948 (genotype 10, 11) and K562 (genotype 9, 10) was amplified with forward primers CSFFW120 (120nt) or CSFFW200 (200nt) and reverse primer CSFRV60J (60nt, JOE-labelled) and then analyzed by capillary electrophoresis (Fig. 3A).

PCR amplifications were run in a MultiGene™ Gradient thermal cycler (Labnet) in a 20μL reaction volume containing 1X Colorless GoTaq® reaction buffer including 1.5mM $MgCl_2$ (Promega), 200uM each deoxyribonucleotide triphosphate, (Promega), 1 U GoTaq® Hot Start DNA Polymerase (Promega), 500nM of each primer (IDT) and 500pg of genomic DNA template. Cycling conditions encompassed an initial denaturation step of 2 min at 94ºC, followed by 35 cycles of 10 seconds at 94ºC, 60 seconds at 59ºC and 45 seconds at 72ºC and a final incubation of 10 minutes at 60ºC. Sample dilutions of the PCR products were run in an ABI 3500 sequencer (Applied Biosystems) with a 50cm-capillary column and POP7 resin. The injection time was 5 seconds. The sample volume was 0.5μL or 1μL, formamide 9.0μl, and CC5 Internal lane Standard (ILS) (Promega) 0.5μL. The software was GeneMapper® ID-X 1.2.

All profiles agreed with the ones described in the literature for these cell types. Moreover, they showed the characteristic heterozygous imbalance present in cell lines K562 (dominant 10 in 9, 10) and 9948 (dominant 10 in 10, 11). There is also a small peak in cell line 9948 (Fig. 3B) that may represent the allele 12 triplet of CSF1PO which has a relative intensity less than 10 % of the dominant allele 10.

EXAMPLE 3

Amplification of STR *loci* in a Multiplex Format.

Figure 4A shows the amplification products for *loci* CSF1PO, Penta E and DYS391 on 2800M DNA using the following sets of primers pairs: CSFFW120 / CSFRV60J, PEFW120 / PERV60F and DYS391F2 / DYSRV120 (Fig. 4B). These primer pairs were in turn successfully combined to produce all possible duplex combinations and a triplex.

End-point PCR amplifications were run in a MultiGene™ Gradient thermal cycler (Labnet) in a 20µL reaction volume containing 1X Colorless GoTaq® reaction buffer including 1.5mM $MgCl_2$ (Promega), 200uM each deoxyribonucleotide triphosphate, (Promega), 1 U GoTaq® Hot Start DNA Polymerase (Promega), 500nM of each primer (IDT) and 500pg of genomic DNA standard 2800M (Promega). Cycling conditions encompassed an initial denaturation step of 2 min at 94ºC, followed by 35 cycles of 10 seconds at 94ºC, 60 seconds at 59ºC and 45 seconds at 72ºC and a final incubation of 10 minutes at 60ºC. PCR products were electrophoresed through a 2% agarose gel in 1X TBE Buffer at 100 Volts constant voltage and visualized with ethidium bromide. 3µL of sample or DNA size standard (100bp Ladder, Promega) were loaded per well.

Figure 5 shows the CE profile of the triplex assay with the expected peak profiles for DYS391 and Penta E (FAM channel) and CSF1PO (JOE channel). Sample dilutions of the PCR products were run in an ABI 3500 sequencer (Applied Biosystems) with a 50cm-capillary column and POP7 resin. The injection time was 5 seconds. The sample volume was 0.5µL or 1µL, formamide 9.0µl, and CC5 Internal lane Standard (ILS) (Promega) 0.5µL. The software was GeneMapper® ID-X 1.2.

Commonly used STR *loci* DYS391, Penta E and CSF1PO were successfully amplified using long ssDNA primers and combined in several multiplex formats.

EXAMPLE 4

PCR Amplification Using Long ssDNA Primers with Partial, Non-complementary Sequences.

Figure 6 shows the amplification of the DYS391 STR region with forward primer DYS391F2 and reverse primers of 60 nucleotides (DYSRV60), 120 nucleotides (DYSRV120) or 200 nucleotides (DYSRV200). These three reverse primers share the same 3’-sequence and are fully complementary to

the DYS391 region. In order to verify that non-complementary sequences may also be successfully utilized, we also included reverse primers DYSRV23+M13 (60nt) and DYSRV120+M13 (200nt) sharing the same, homologous 3’ priming sequence with the other reverse primers, but having a non-complementary M13 tag of 37nt and 80nt, respectively, at their 5’-end region.

End-point PCR amplifications were run in a MultiGene™ Gradient thermal cycler (Labnet) in a 20µL reaction volume containing 1X Colorless GoTaq® reaction buffer including 1.5mM $MgCl_2$ (Promega), 200uM each deoxyribonucleotide triphosphate, (Promega), 1 U GoTaq® Hot Start DNA Polymerase (Promega), 500nM of each primer (IDT) and 500pg of genomic DNA standard 2800M (Promega). Cycling conditions encompassed an initial denaturation step of 2 min at 94ºC, followed by 35 cycles of 10 seconds at 94ºC, 60 seconds at 59ºC and 45 seconds at 72ºC and a final incubation of 10 minutes at 60ºC. PCR products were electrophoresed through a 2% agarose gel in 1X TBE Buffer at 100 Volts constant voltage and visualized with ethidium bromide. 3µL of sample or DNA size standard (100bp Ladder, Promega) were loaded per well.

Long ssDNA primers that are complementary to the target only in the 3’ priming region were successfully amplified in the STR DYS391 region yielding products of the expected size.

EXAMPLE 5

Addition of Long ssDNA Primers in Complex Multiplex PCR Assays

In order to know if the long ssDNA polynucleotides might somehow interfere in assays involving several primers, we added primer pairs DYS391F2/ DYSRV120 (0.8µM final concentration each) and DYS391F2 / DYSRV200 (0.8µM final concentration each) (Fig. 7A) into a commercial kit containing already 42 primers (PowerPlex® 21, Promega). PowerPlex® 21 (Promega) PCR reactions were done according to the manufacturer’s instructions in an ABI 9700 thermal cycler (Applied Biosystems Inc.), except that the reaction final volume was 12.5µL. Sample dilutions of the PCR products were run in an ABI 3500 sequencer (Applied Biosystems) with a 50cm-capillary column and POP7 resin. The injection time was 5 seconds. The sample volume was 0.5µL or 1µL, formamide 9.0µl, and CC5 Internal lane Standard (ILS) (Promega) 0.5µL. The software was GeneMapper® ID-X 1.2.

Figure 7B shows that the 2800M DNA profile for the 21 markers is not altered at all by the addition of the long primers, while a peak corresponding to DYS391 is identified in the FAM channel at the expected size for both pairs of primers. The secondary peak in the amplification of DYSRV200 (200nt) may be due to an incomplete n-1 PAGE post-synthesis purification of the primer. Long ssDNA primers are then fully compatible with more complex multiplex PCR assays.

EXAMPLE 6

PCR Amplification of Degraded DNA with Long ssDNA Primers.

To demonstrate the advantage of using long primers on fragmented or degraded DNA samples, genomic DNA was subjected to a controlled degradation. 4ng of 2800M DNA (Promega) was incubated for 0, 10, and 30 minutes at 95ºC in 40µL of nuclease-free water. Samples were immediately chilled out after each time-point. A qPCR amplification of the region of CSF1PO *locus* using primer pair CSFFW2416HSJ/CSFRV2216HS was performed on 500pg of DNA for each time-point in order to assess the degree of degradation by comparing the different Ct values. The resulting degraded DNA was qPCR amplified using either the CSF1PO primers CSFFW2416HSJ/CSFRV2216HS from PowerPlex®16HS or the longer ssDNA pair of primers CSFFW200 (200nt) and JOE-labelled CSFRV60J (60nt) (Fig. 2B).

Quantitative PCR was performed in a Rotor-Gene RG-6000 cycler (Qiagen) in a 20µL reaction volume containing 1X qPCR GoTaq® qPCR Master Mix (Promega), 500nM of each primer (IDT) and 500pg of heat-treated 2800M genomic DNA (Promega). Cycling conditions included an initial denaturation step of 2 min at 94ºC, followed by 38 cycles of 10 seconds at 94ºC, 60 seconds at 59ºC and 45 seconds at 72ºC and a final incubation of 10 minutes at 60ºC. Fluorescent readings were detected at 470nm excitation / 510nm emission wavelengths.

Figure 8 shows the qPCR profiles for both pair of primers with DNA treated for different periods of time. The shorter PowerPlex®16HS primers gave an initial lower Ct (cycle threshold) value with intact DNA (time 0). However, the DNA template heat-treated for only 10 minutes already shows a similar Ct for both pair of primers. For more degraded DNA, the Ct in the reaction with the long ssDNA primers becomes lower than with the shorter commercial primers.

The qPCR profiles show that long ssDNA primers are advantageous for amplifying degraded DNA.

EXAMPLE 7

Amplification of Degraded DNA Using Long ssDNA Primers in Complex Multiplex PCR Assays Containing CODIS Markers

To show the advantage of using long ssDNA primers on fragmented or degraded DNA samples in complex multiplex PCR assays, genomic DNA was subjected to a controlled degradation for different period of times and then amplified with the commercial kit PowerPlex® Fusion 6C (Promega), which already contains 54 primers, in the presence or absence of the long primer pair CSFFW200/CSFRV60J. 2800M DNA (4ng) was incubated for 0, 10, 30, 45 and 60 minutes at 95ºC in 40µL of nuclease-free water. Samples were immediately chilled out after each time-point. A qPCR amplification on the region of the CSF1PO *locus* was performed using the primer pair CSFFW200/CSFRV60J with 500pg of treated DNA for each time-point in order to assess the degree of degradation by comparing the different Ct values (Fig. 9A). Quantitative PCR was performed in a

Rotor-Gene RG-6000 cycler (Qiagen) in a 20µL reaction volume containing 1X qPCR GoTaq® qPCR Master Mix (Promega), 500nM of each primer (IDT) and 500pg of heat-treated 2800M genomic DNA (Promega). Cycling conditions included an initial denaturation step of 2 min at 94ºC, followed by 35 cycles of 10 seconds at 94ºC, 60 seconds at 59ºC and 45 seconds at 72ºC and a final incubation of 10 minutes at 60ºC. Fluorescent readings were detected at 470nm excitation / 510nm emission wavelengths.

Time points of 0, 30 and 60 minutes – and an NTC (no template control) – were amplified using the commercial kit PowerPlex® Fusion 6C in the presence or absence of the long primer pair CSFFW200/CSFRV60J (0.8µM final concentration each). PowerPlex® Fusion 6C PCR reactions were done according to the manufacturer's instructions in an ABI 9700 thermal cycler (Applied Biosystems Inc.), except that the reaction final volume was 12.5µL. Sample dilutions of the PCR products were run in an ABI 3500 sequencer (Applied Biosystems Inc.) with a 50cm-capillary column and POP7 resin. The injection time was 5 seconds. The sample volume was 0.5µL or 1µL, formamide 9.0µl, and WEN Internal lane Standard (ILS) (Promega) 0.5µL. The software was GeneMapper® ID-X 1.2.

Figure 9B shows that the CSF1PO marker is detected at the 60 minute time-point only when using the long primer pair CSFFW200/CSFRV60J (JOE channel). This example demonstrates the advantage of using *superprimers* when performing complex multiplex assays containing CODIS markers in the presence of degraded DNA samples.

EXAMPLE 8

End-point PCR Using Very Long Primers of 260 and 300 nucleotides

A PCR amplification of the *locus* CSF1PO was performed using very long ssDNA polynucleotides of 260 nucleotides (CSFFW260) or 300 nucleotides (CSFFW300) as forward primers and a primer of 60 nucleotides (CSFRV60J) as reverse primer. A control amplification (370bp) using primers CSFFW200/CSFRV60J was included. (Fig. 10B).

End-point PCR amplifications were run in a MultiGene™ Gradient thermal cycler (Labnet) in a 20µL reaction volume containing 1X Colorless GoTaq® reaction buffer including 1.5mM $MgCl_2$ (Promega), 0.625mM of additionally supplemented $MgCl_2$ (Promega), 200uM each deoxyribonucleotide triphosphate, (Promega), 2.5 U of GoTaq® Hot Start DNA Polymerase (Promega), 250nM of each primer (IDT) and 1ng of genomic DNA standard 2800M (Promega). Cycling conditions encompassed an initial denaturation step of 2 min at 94ºC, followed by 35 cycles of 10 seconds at 94ºC, 60 seconds at 59ºC and 45 seconds at 72ºC and a final incubation of 10 minutes at 60ºC. PCR products were electrophoresed through a 2% agarose gel in 1X TBE Buffer at 100 Volts constant voltage and

visualized with ethidium bromide. 3µL of sample or DNA size standard (100bp Ladder, Promega) were loaded per well.

The gel electrophoresis shows that very long ssDNA primers of 260 and 300 nucleotides produce the expected PCR products of 370bp and 410bp respectively, without showing any significant artifacts or spurious bands (Fig.10A).

In the above description, for purposes of explanation, numerous details are set forth in order to provide a thorough understanding of the present disclosure. However, it will be apparent to one skilled in the art that these specific details are not required in order to practice the present disclosure. Although certain dimensions and materials are described for implementing the disclosed example embodiments, other suitable dimensions and/or materials may be used within the scope of this disclosure. All such modifications and variations, including all suitable current and future changes in technology, are believed to be within the sphere and scope of the present disclosure. All references mentioned are hereby incorporated by reference in their entirety

CLAIMS

1. A method to perform a polymerase chain reaction (PCR) comprising the following steps:

a. Providing a nucleic acid sample;

b. Hybridizing said nucleic acid sample to one or more pair of primers where at least one primer consists of a single stranded DNA polynucleotide having a length of 60 or more nucleotides;

c. Subjecting said nucleic acid sample to a PCR, wherein the reaction mixture medium contains at least one of said primers; and

d. Detecting the amplified products.

2. The method according to claim 1 wherein the nucleic acid is genomic DNA.

3. The method according to claim 2 wherein the sequence of genomic DNA is a genetic marker selected from the group consisting of genes, identification markers and inherited disease markers.

4. The method according to claim 2 wherein the nucleic acid is degraded or fragmented.

5. The method according to claim 1 wherein at least one primer has a length between 60 and 300 nucleotides.

6. The method according to claim 1 wherein the sequence of at least one of the primers is fully complementary to the target sequence.

7. The method according to claim 1 wherein at least one of the primers has non-homologous sequences at the 5’ end, having its 3´-priming region complementary to the target sequence.

8. The method according to claim 1 wherein the target sequence is a STR genetic marker selected from the group consisting of CSF1PO, DYS391 and Penta E and a combination thereof.

9. The method according to claim 1 wherein said method comprises the hybridization of more than one pair of primers in order to simultaneously amplify more than one target sequence, where each target sequence is a different genetic marker, being all the primers and target sequences in the same reaction mixture.

10. The method according to claim 1 wherein the PCR assay is selected from a group consisting of monoplex, multiplex, end-point, real-time, hot start and non-hot start.

11. The method according to claim 1 wherein at least one primer is labelled by one of the group consisting of a fluorescent dye, luminescent moiety, enzyme, hapten and antigen.

12. The method according to claim 1 wherein the nucleic acid is selected from the group consisting of mitochondrial DNA, plasmid DNA, viral DNA or RNA, circulating cell-free DNA, synthetic DNA and messenger RNA.

13. A method to detect in a degraded nucleic acid sample one or more genetic markers by performing a polymerase chain reaction (PCR) comprising the following steps:

a. Providing a nucleic acid sample;

b. Hybridizing said degraded nucleic acid sample to one or more pair of primers where at least one primer consists of a single stranded DNA polynucleotide having a length of 60 or more nucleotides;

c. Subjecting said nucleic acid sample to a PCR, wherein the reaction mixture medium contains at least one of said primers; and

d. Detecting the amplified products.

14. The method according to claim 13 wherein the target sequence is a STR genetic marker selected from the group consisting of CSF1PO, DYS391, Penta E and a combination thereof.

15. The method according to claim 13 wherein at least one primer has a length between 60 and 300 nucleotides.

16. The method according to claim 13 wherein the sequence of at least one of the primers is fully complementary to the target sequence.

17. The method according to claim 13 wherein at least one of the primers has non-homologous sequences at the 5’ end, having its 3´-priming region complementary to the target sequence.

18. The method according to claim 13 wherein at least one primer is labelled by one of the group consisting of fluorescent dye, luminescent moiety, enzyme, hapten and antigen.

19. The method according to claim 13 wherein the PCR assay is selected from a group consisting of monoplex, multiplex, end-point, real-time, hot start and non-hot start.

20. A kit comprising of at least one pair of primers, wherein at least one primer has a length of 60 nucleotides or more.

21. A kit according to claim 20 wherein said kit comprises more than one pair of primers in order to simultaneously amplify more than one target sequence, where each target sequence is a different genetic marker, being all the primers and target sequences in the same reaction mixture.

22. A kit according to claim 20 comprising at least one DNA polymerase enzyme, dNTPs (deoxyribonucleotide triphosphate), buffers, salts, control template and size standards.

23. A kit according to claim 21 wherein the genetic markers belong to the Combined DNA Index System (CODIS) of the Federal Bureau of Investigation (FBI) database.

ABSTRACT

Method to perform a PCR assay that comprises the following steps: a. Obtaining a nucleic acid sample; b. Hybridizing that nucleic acid sample to one or more pair of primers where at least one primer consists of a single stranded DNA polynucleotide having a length of 60 or more nucleotides; c Subjecting said nucleic acid sample to a PCR, wherein the reaction mixture medium contains at least one of said primers; and d. Detecting the length of the amplified products. The amplified nucleic acid may contain any sequence or multiple sequences of STRs (short tandem repeats), genes or any coding region having a defined location on a genome. The preferred nucleic acid samples to be amplified are degraded or fragmented and contain one or more genetic markers.

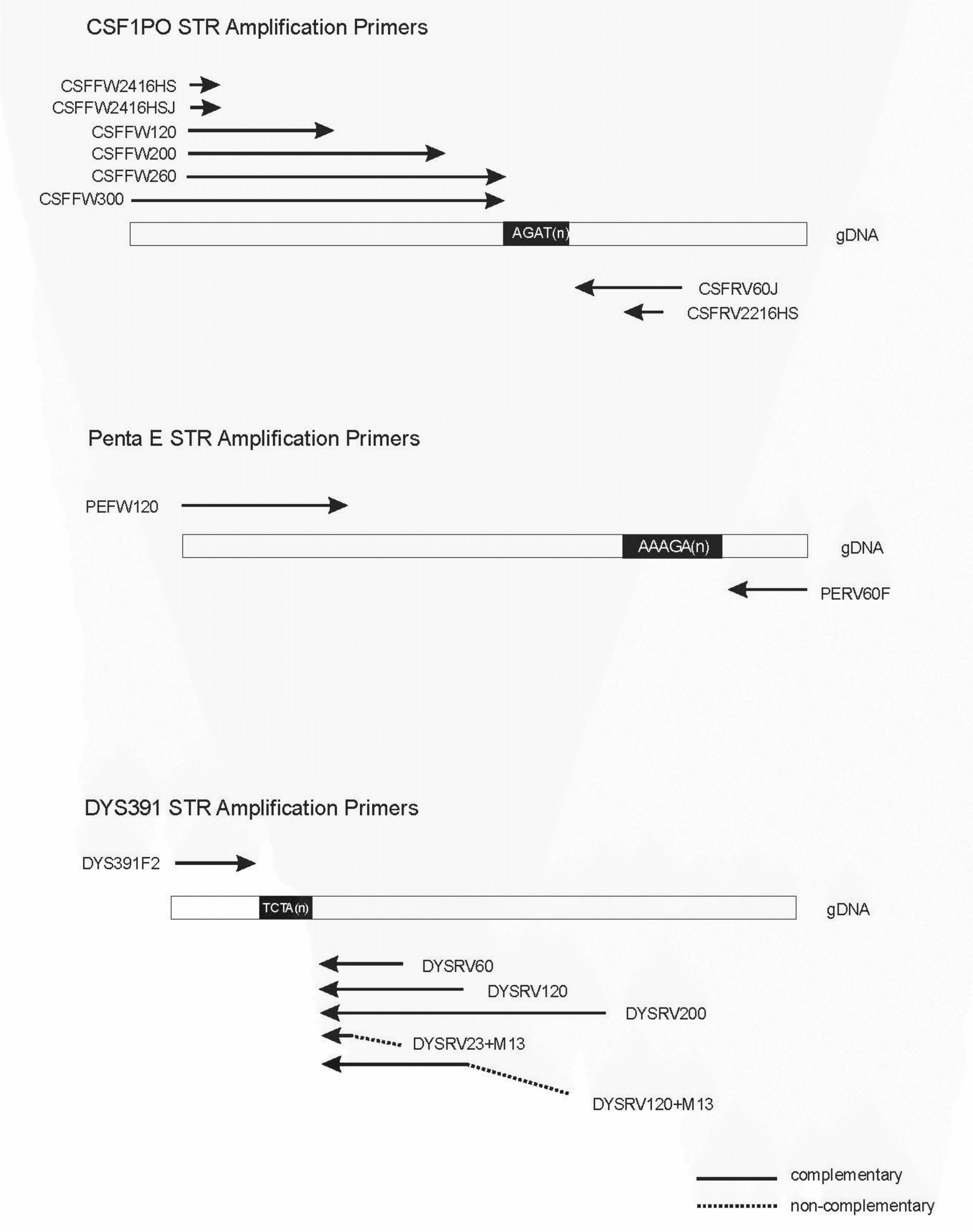
CSF1PO STR Amplification Primers
CSFFW2416HS
CSFFW2416HSJ
CSFFW120
CSFFW200
CSFFW260
CSFFW300
AGAT(n)
gDNA
CSFRV60J
CSFRV2216HS
Penta E STR Amplification Primers
PEFW120
AAAGA(n)
gDNA
PERV60F
DYS391 STR Amplification Primers
DYS391F2
TCTA(n)
gDNA
DYSRV60
DYSRV120
DYSRV200
DYSRV23+M13
DYSRV120+M13
complementary
non-complementary

Figure 1

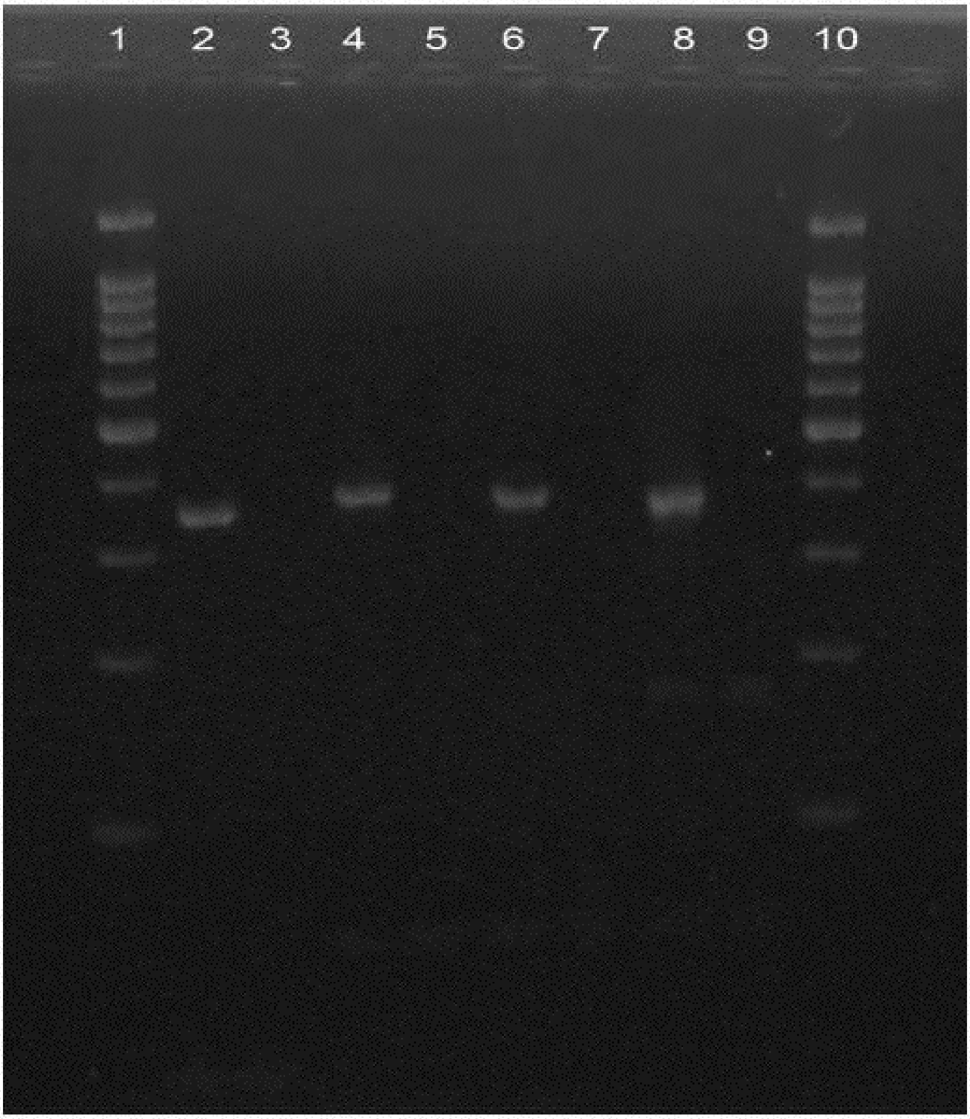

Figure 2A

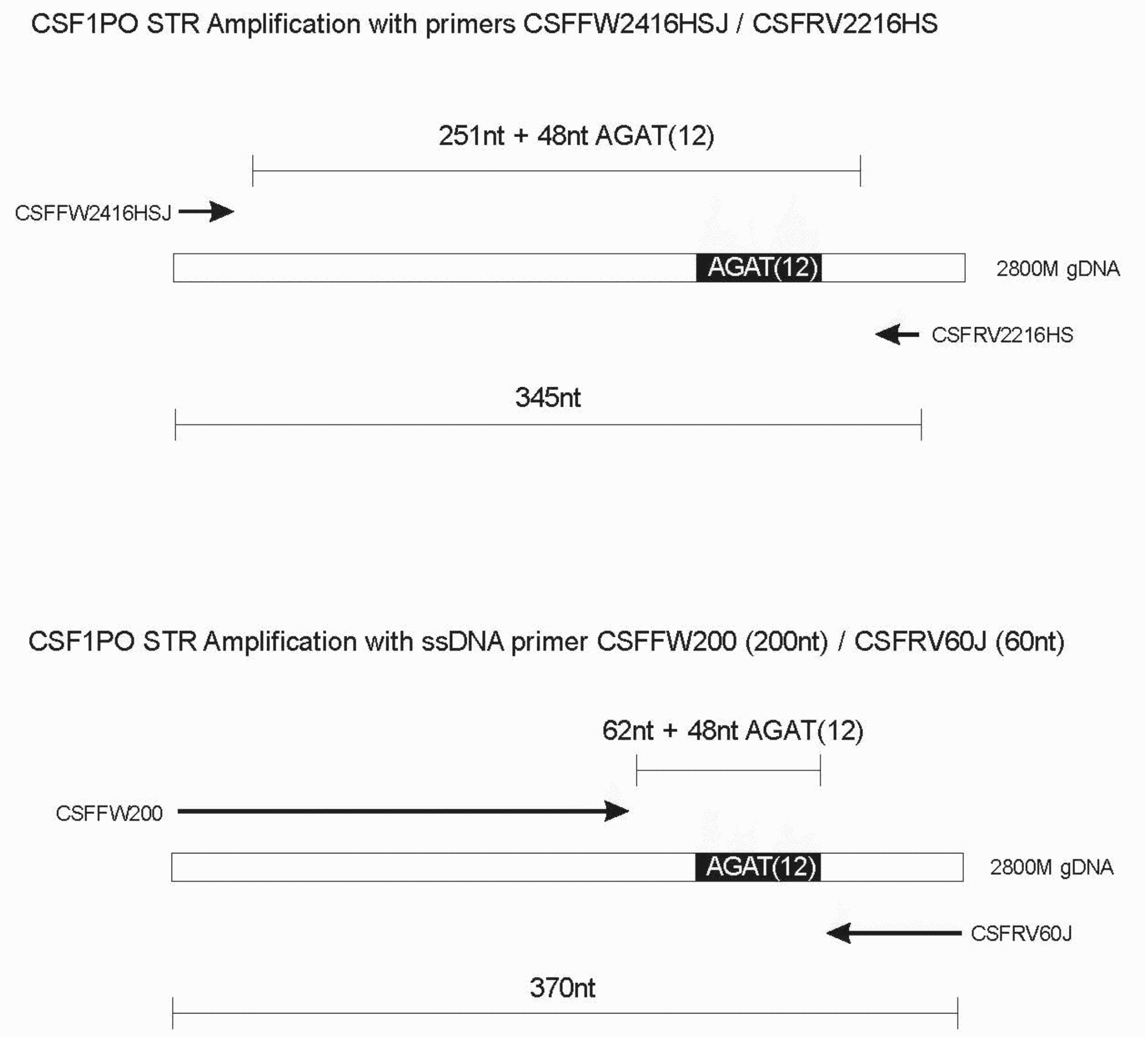

Figure 2B

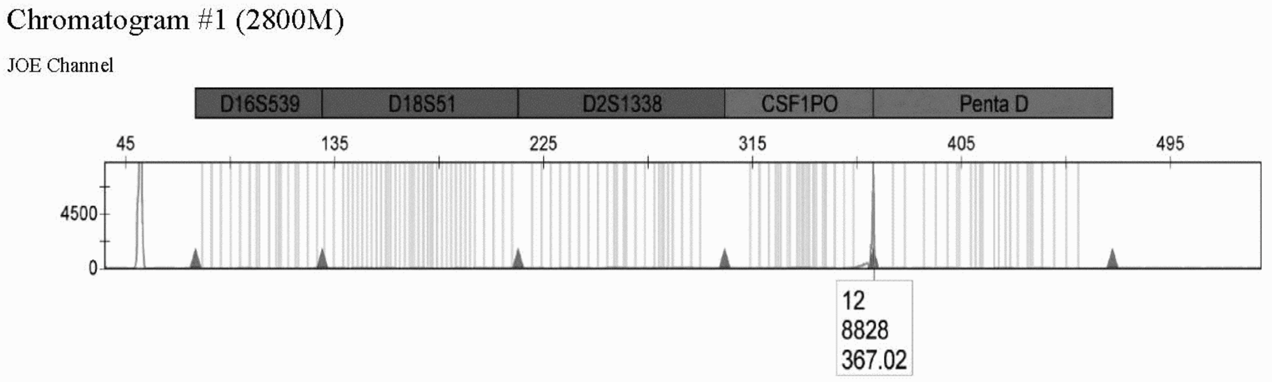

Figure 3A (continued)

Chromatogram #2 (9947A)

JOE Channel

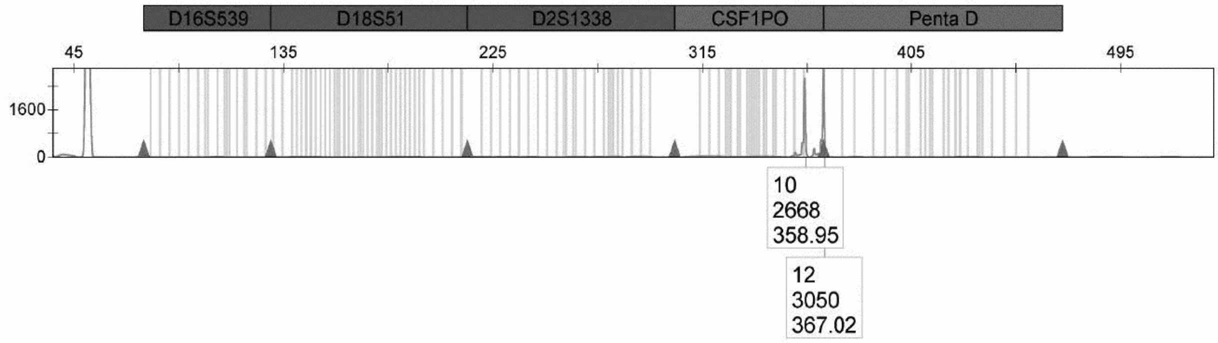

Chromatogram #3 (9948)

JOE Channel

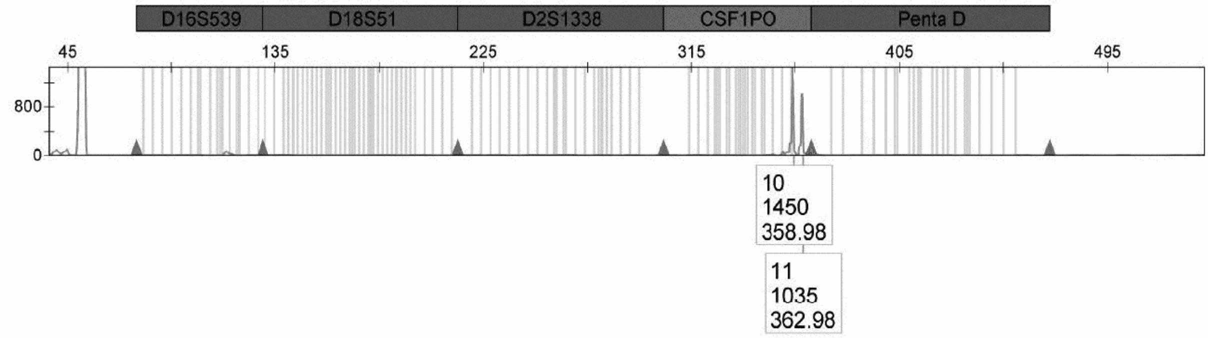

Figure 3A (continued)

Chromatogram #4 (K562)

JOE Channel

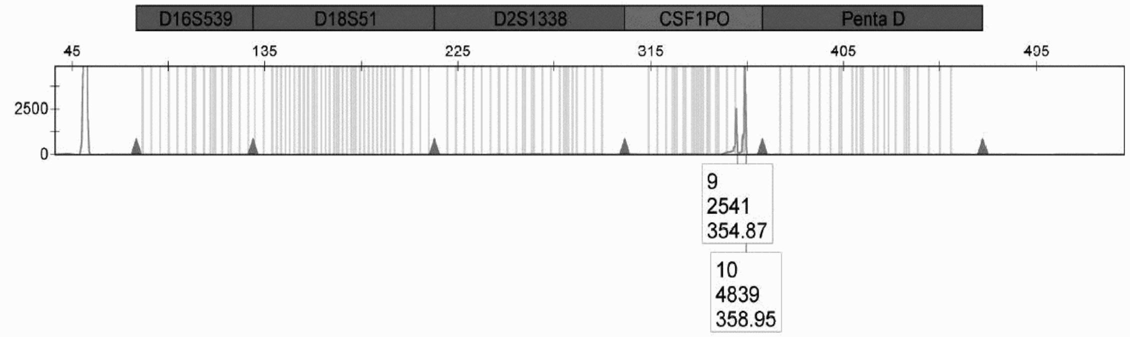

Chromatogram #5 (NTC)

JOE Channel

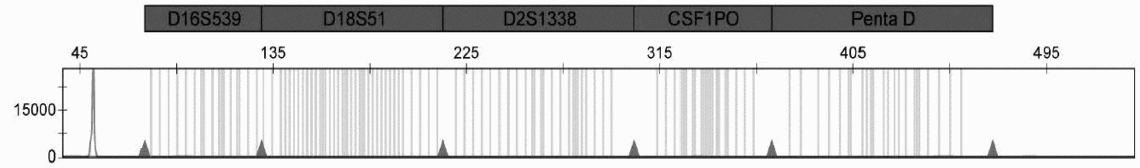

Chromatogram #6 (2800M)

JOE Channel

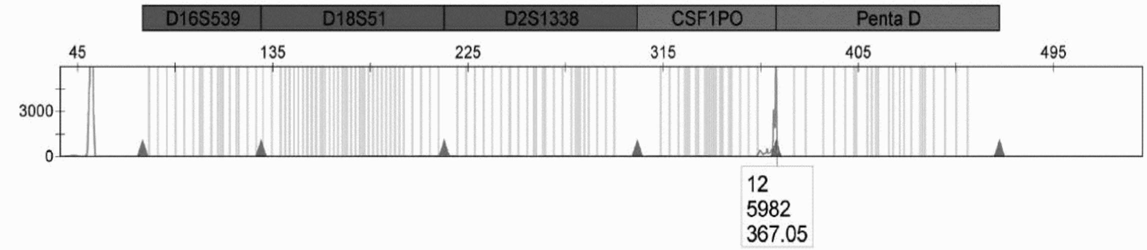

Figure 3A (continued)

Chromatogram #7 (9947A)

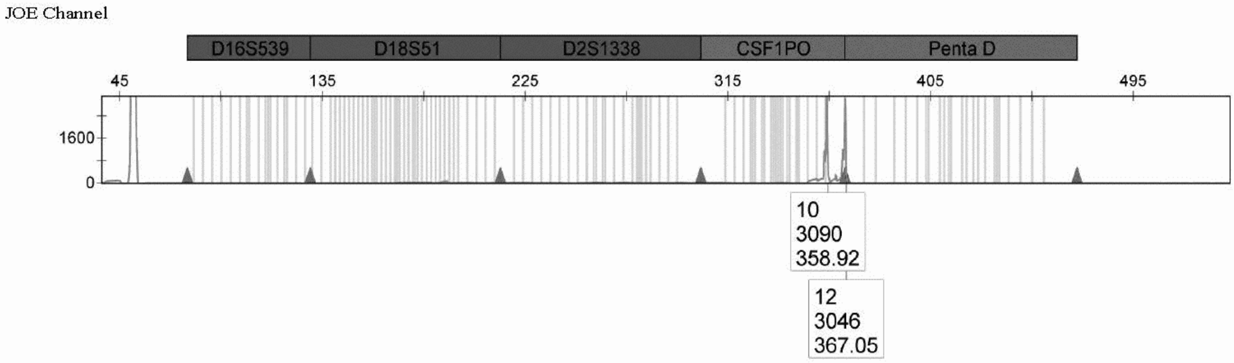

Chromatogram #8 (9948)

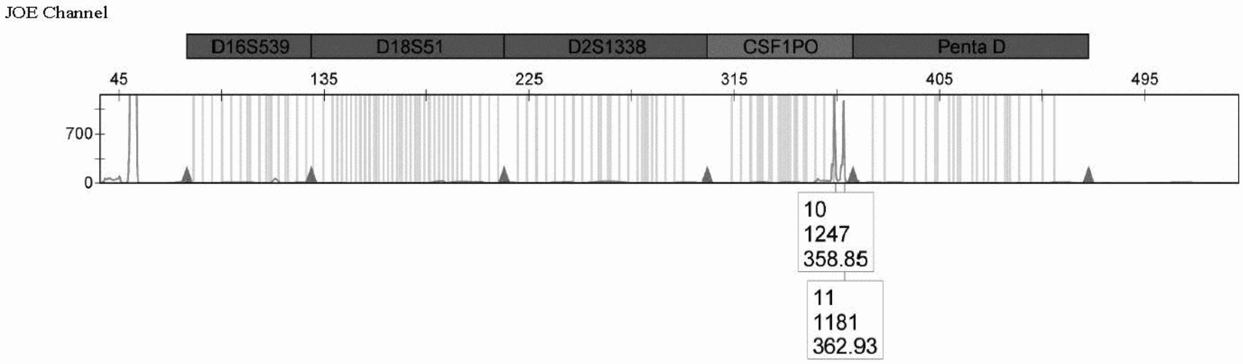

Figure 3A (continued)

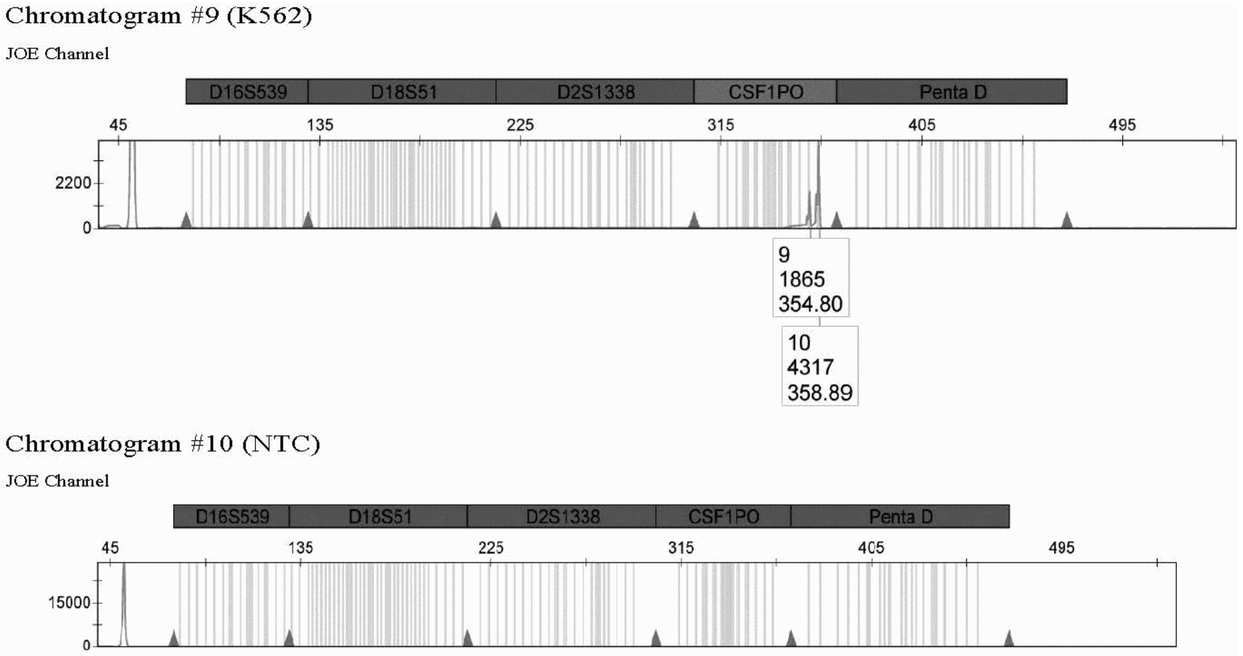

Figure 3A

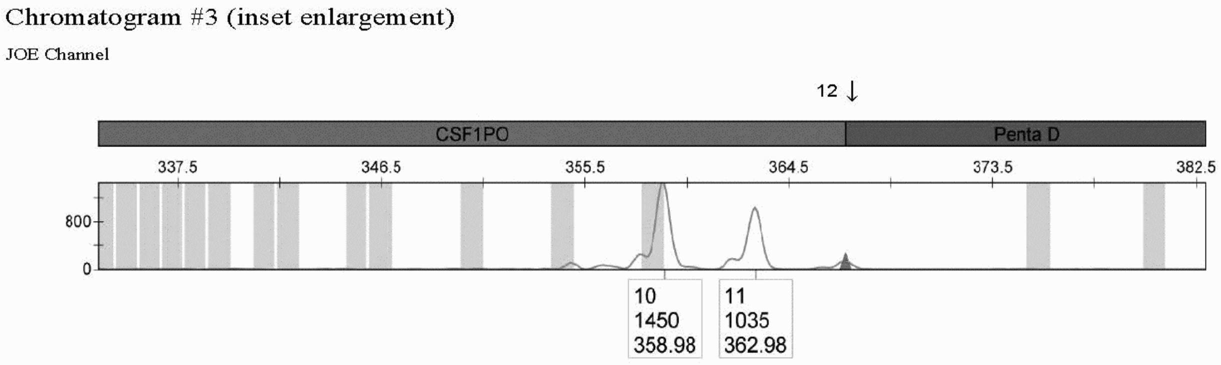

Figure 3B (continued)

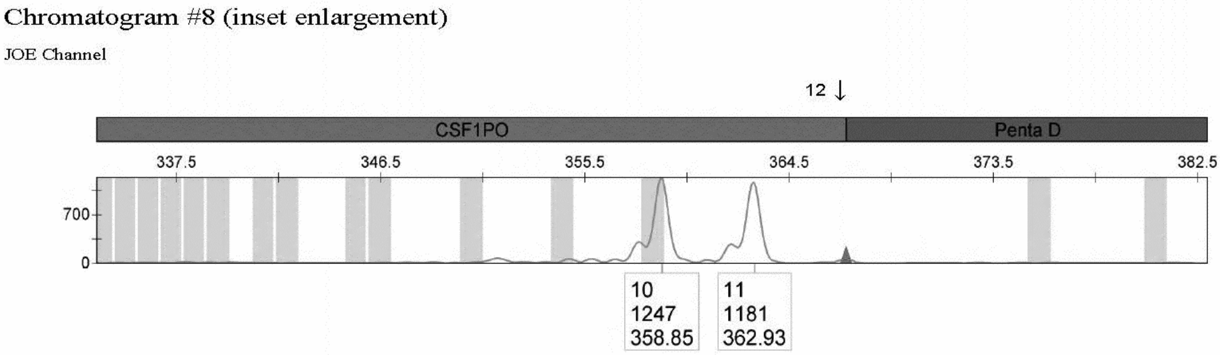

Figure 3B

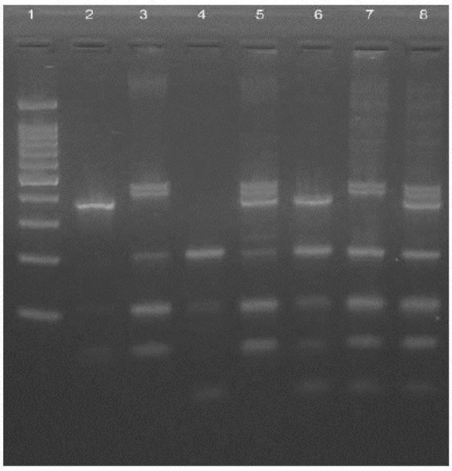

Figure 4A

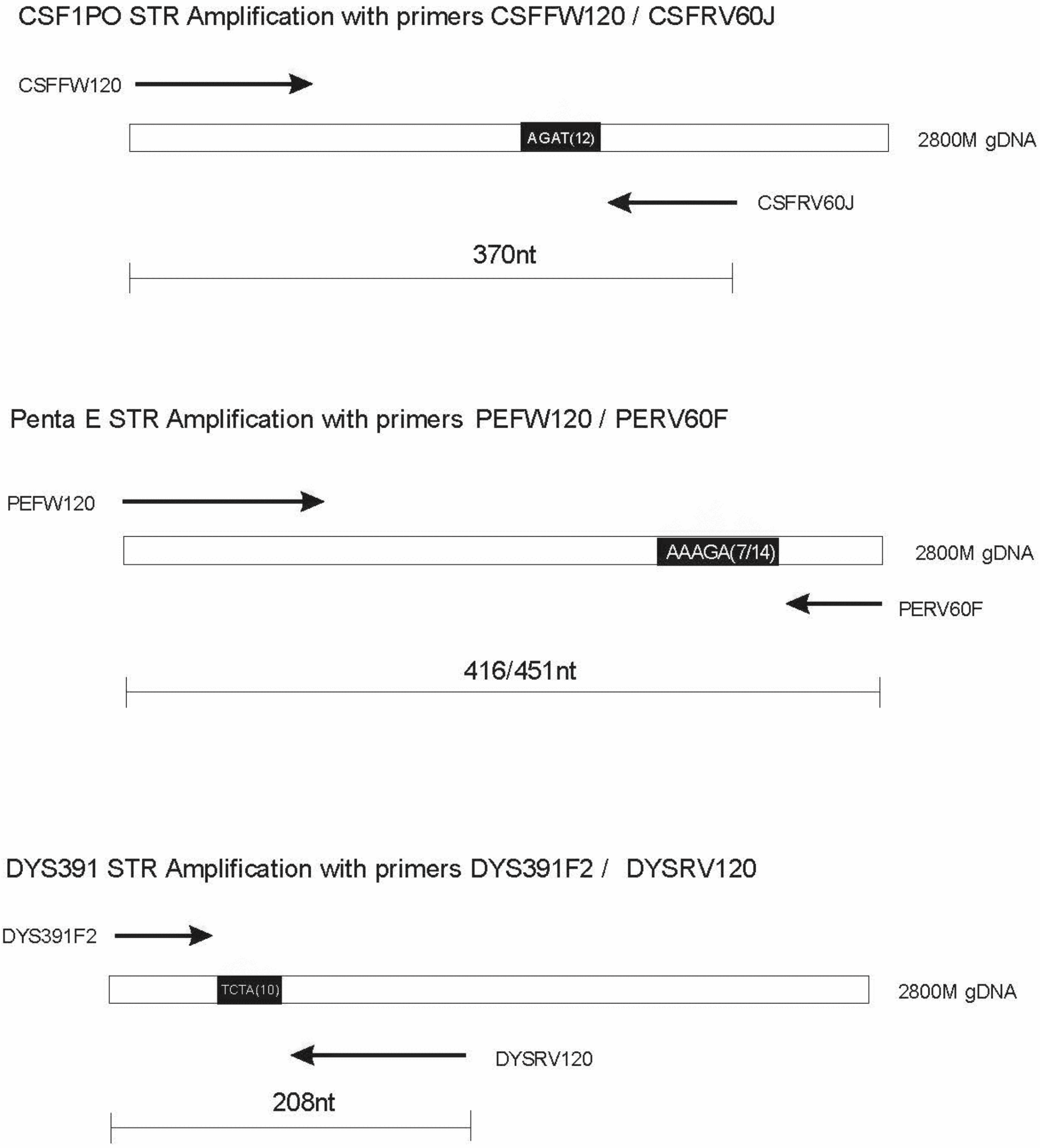
CSF1PO STR Amplification with primers CSFFW120 / CSFRV60J
CSFFW120
AGAT(12)
2800M gDNA
CSFRV60J
370nt
Penta E STR Amplification with primers PEFW120 / PERV60F
PEFW120
AAAGA(7/14)
2800M gDNA
PERV60F
416/451nt
DYS391 STR Amplification with primers DYS391F2 / DYSRV120
DYS391F2
TCTA(10)
2800M gDNA
DYSRV120
208nt

Figure 4B

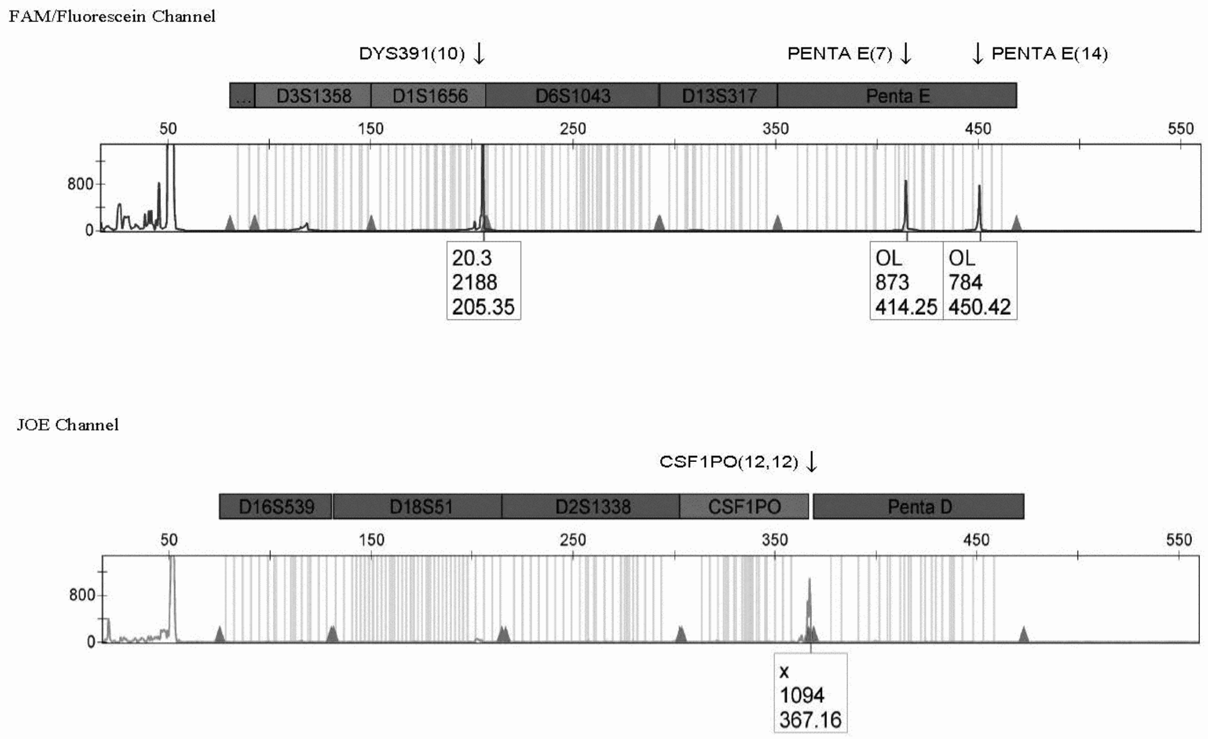
FAM/Fluorescein Channel
DYS391(10)
PENTA E(7)
PENTA E(14)
D3S1358
D1S1656
D6S1043
D13S317
Penta E
50
150
250
350
450
550
800
0
20.3
2188
205.35
OL
873
414.25
OL
784
450.42
JOE Channel
CSF1PO(12,12)
D16S539
D18S51
D2S1338
CSF1PO
Penta D
50
150
250
350
450
550
800
0
x
1094
367.16

Figure 5

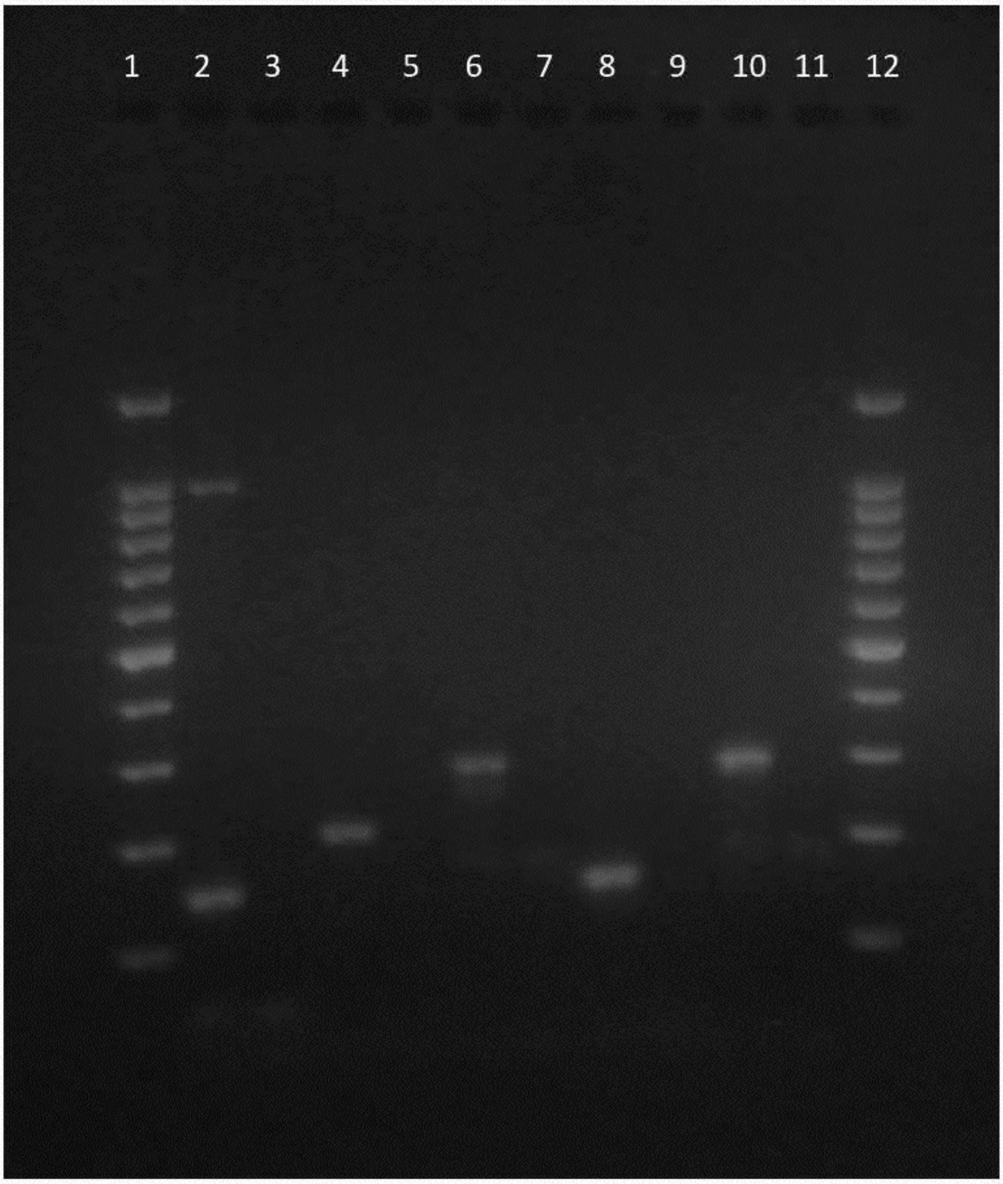

Figure 6

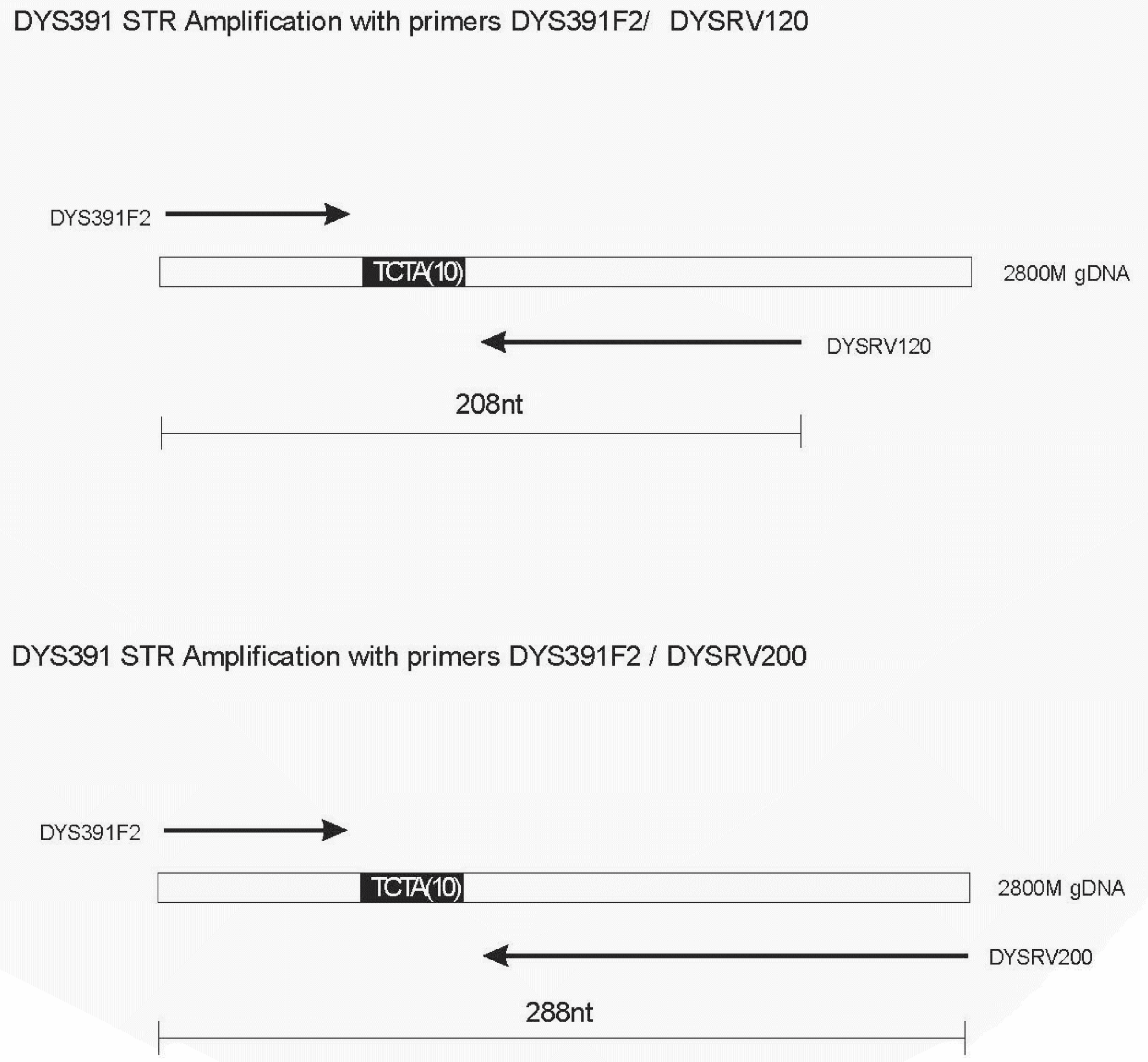
DYS391 STR Amplification with primers DYS391F2/ DYSRV120
DYS391F2
TCTA(10)
2800M gDNA
DYSRV120
208nt
DYS391 STR Amplification with primers DYS391F2 / DYSRV200
DYS391F2
TCTA(10)
2800M gDNA
DYSRV200
288nt

Figure 7A

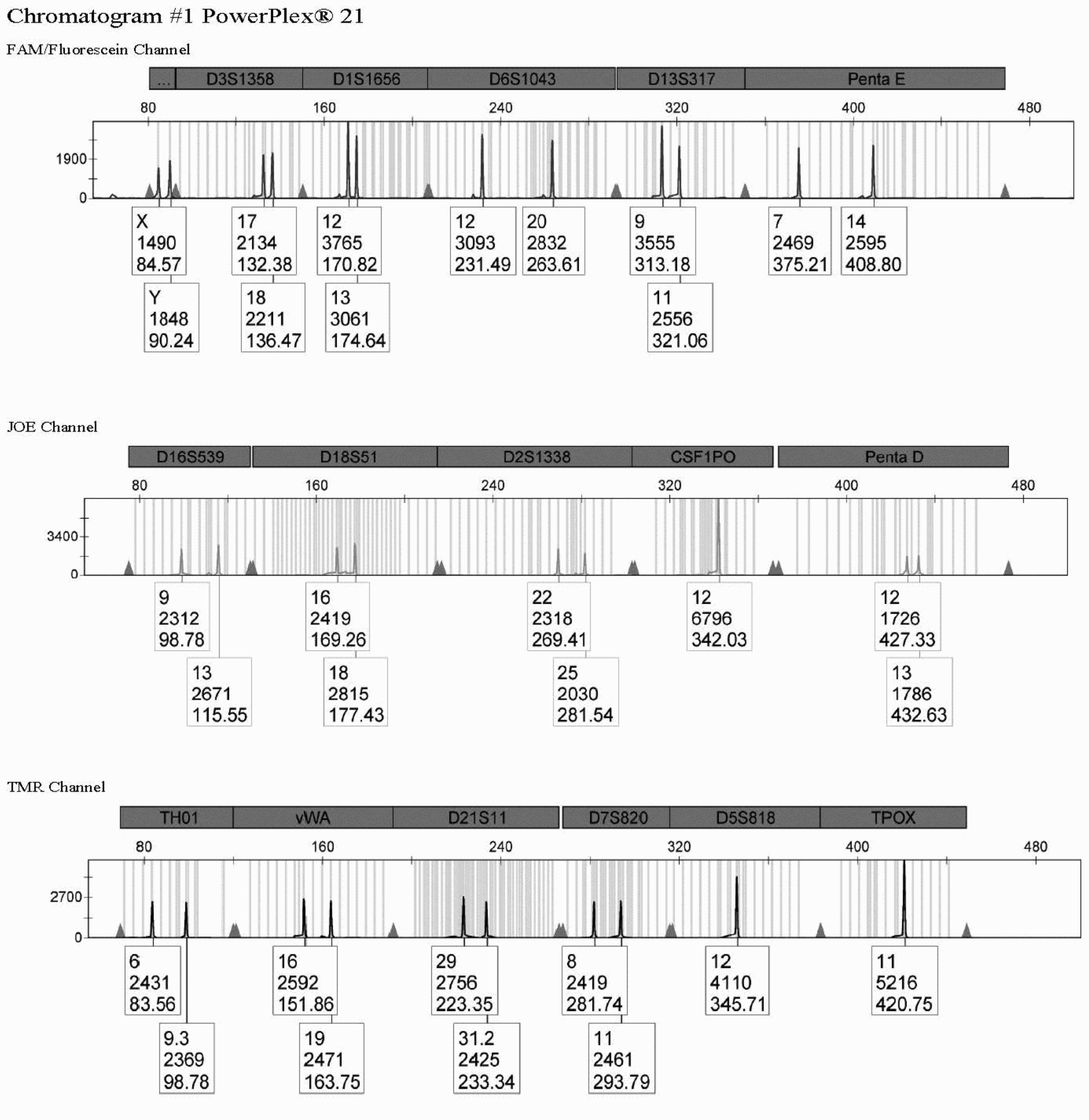

Figure 7B (continued)

CXR Channel

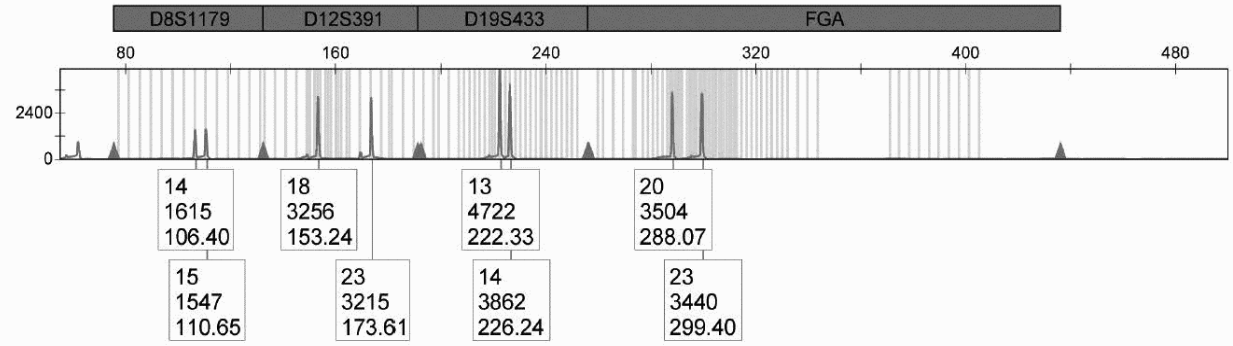

Chromatogram #2 PowerPlex® 21 + DYS391F2 / DYSRV120

FAM/Fluorescein Channel

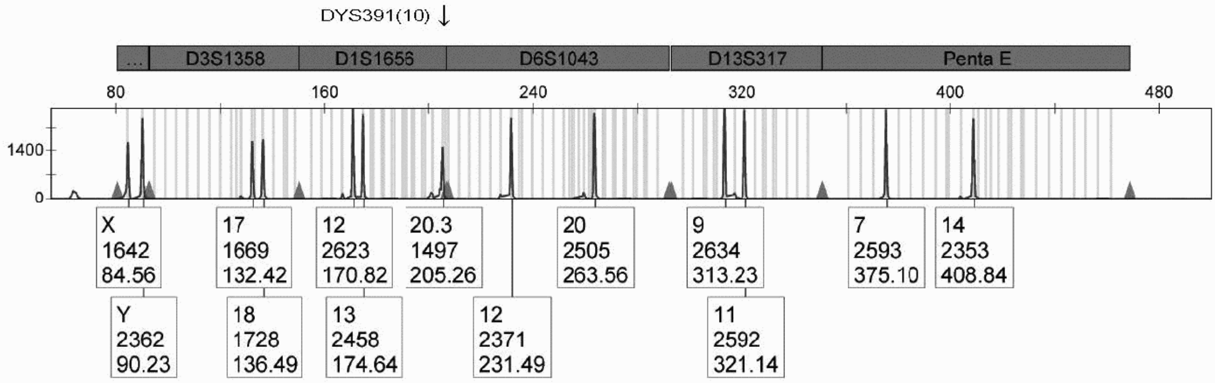

Figure 7B (continued)

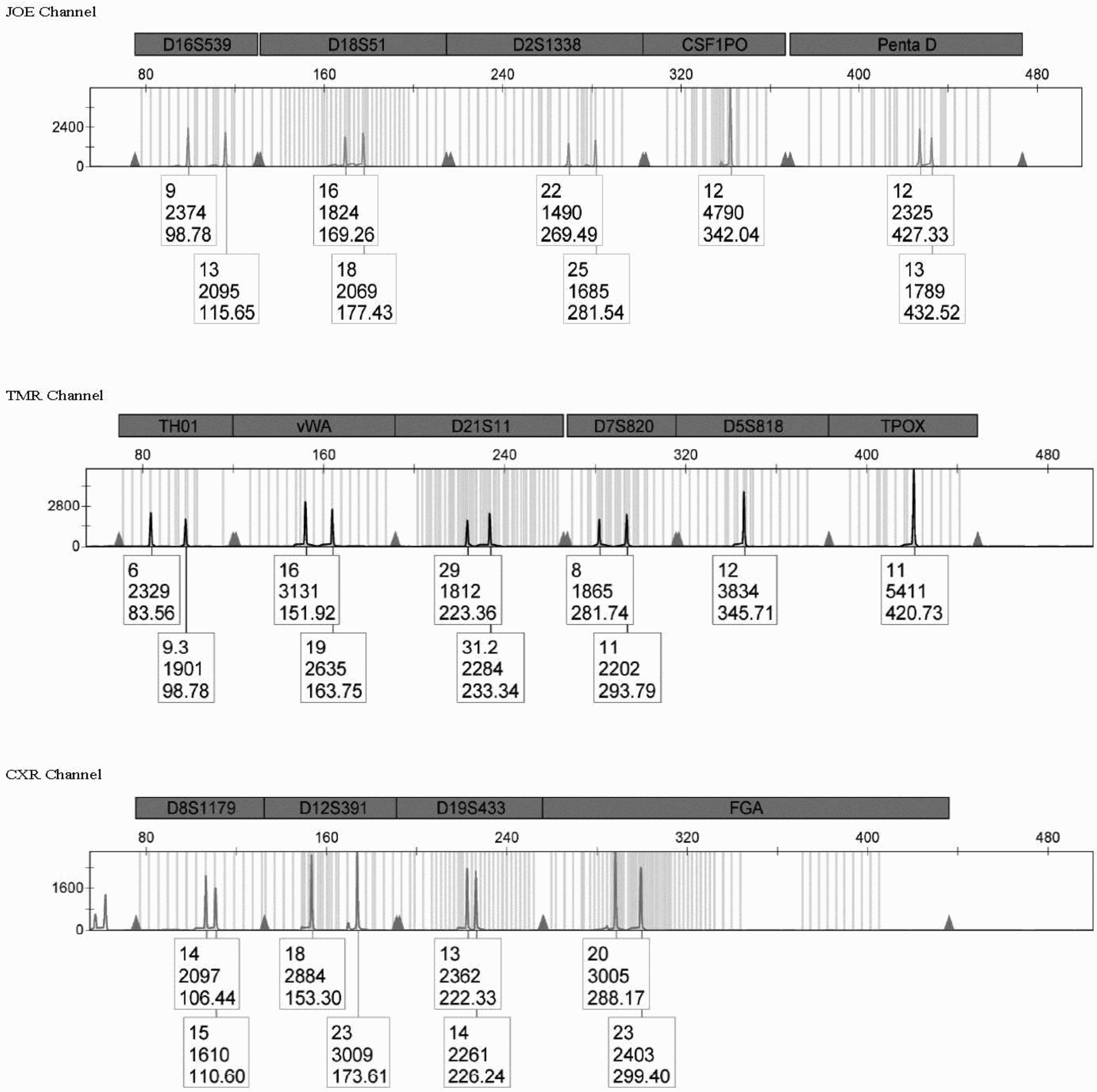

Figure 7B (continued)

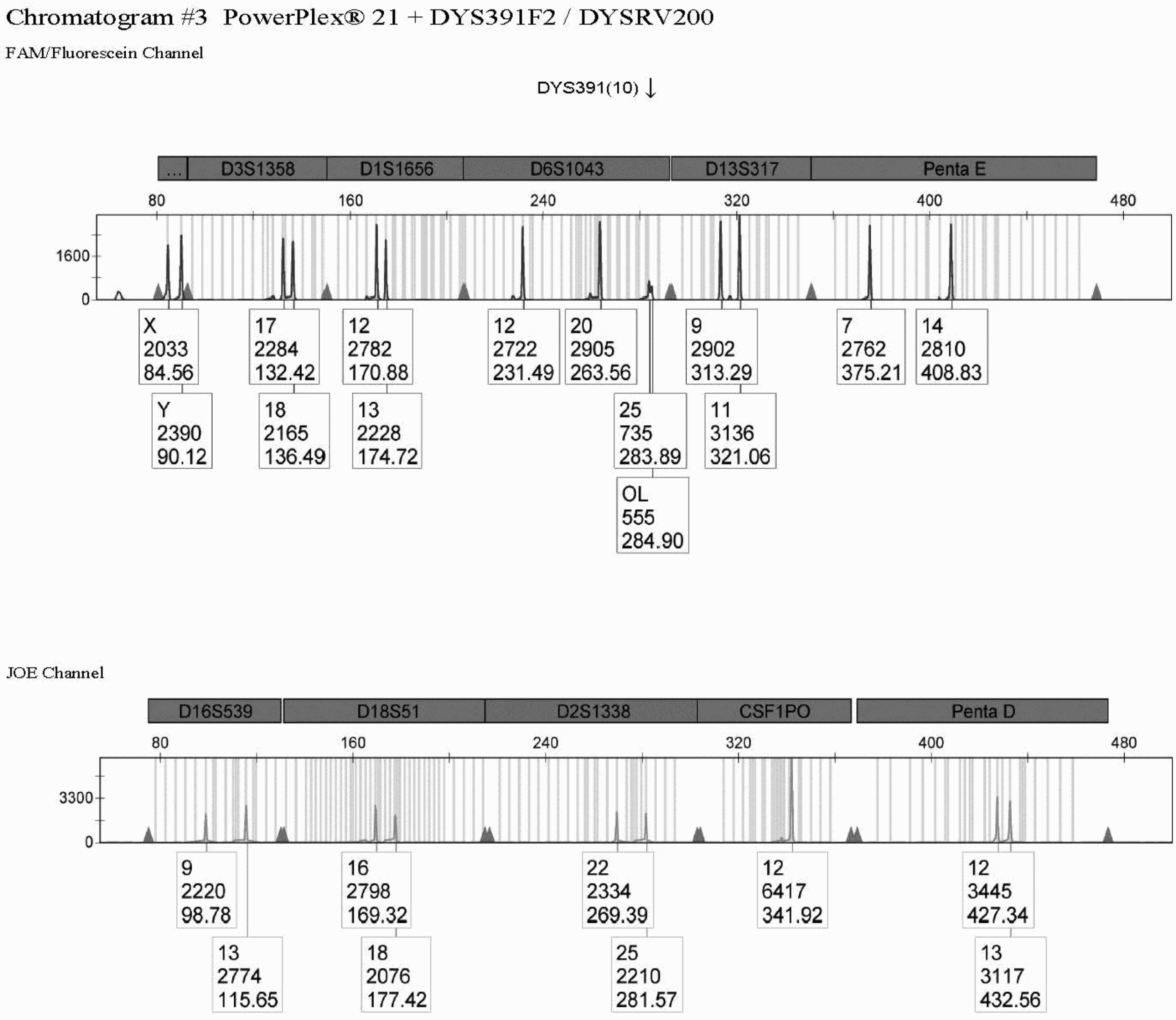

Figure 7B (continued)

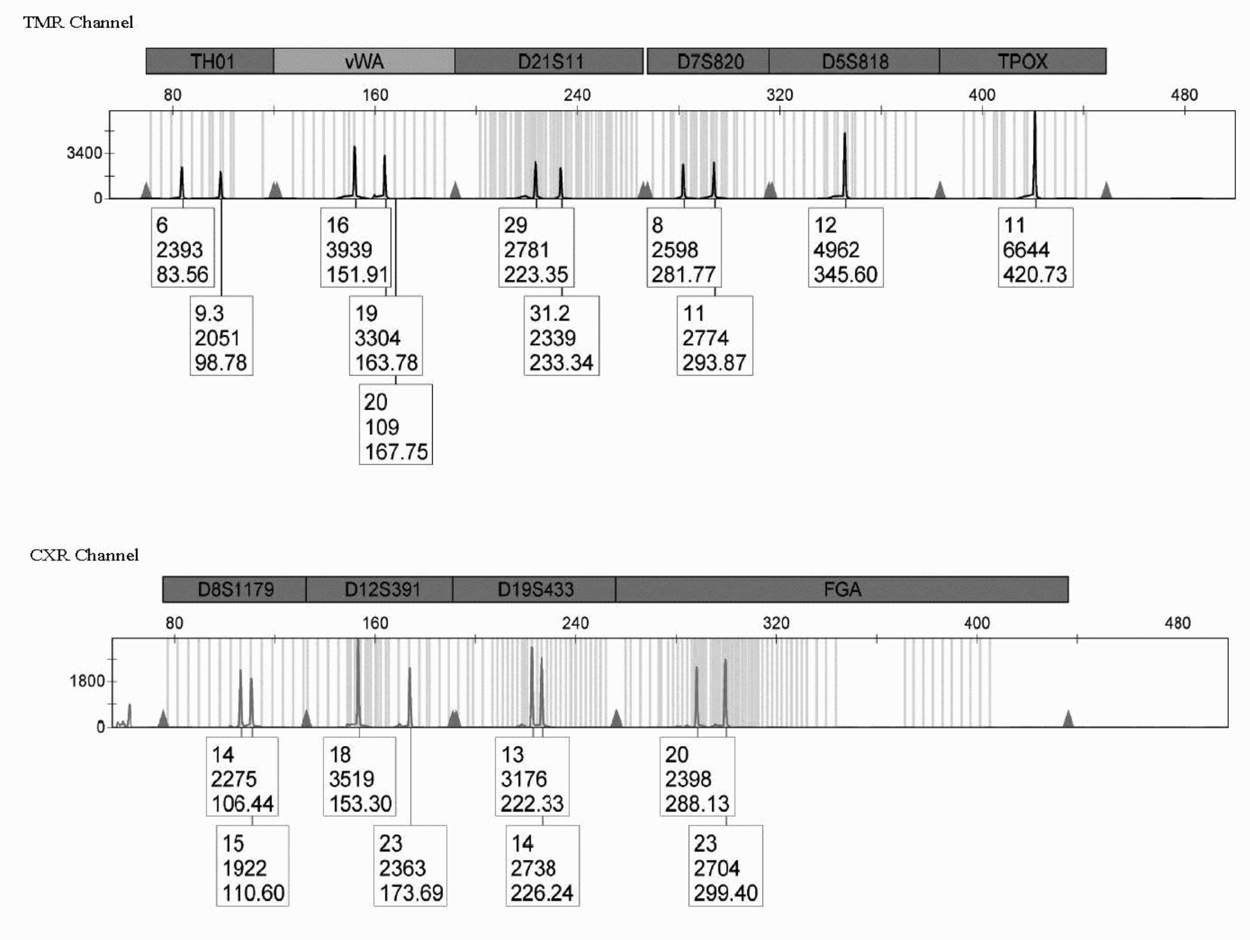
TMR Channel
TH01
vWA
D21S11
D7S820
D5S818
TPOX
80
160
240
320
400
480
3400
0
6
2393
83.56
9.3
2051
98.78
16
3939
151.91
19
3304
163.78
20
109
167.75
29
2781
223.35
31.2
2339
233.34
8
2598
281.77
11
2774
293.87
12
4962
345.60
11
6644
420.73
CXR Channel
D8S1179
D12S391
D19S433
FGA
80
160
240
320
400
480
1800
0
14
2275
106.44
15
1922
110.60
18
3519
153.30
23
2363
173.69
13
3176
222.33
14
2738
226.24
20
2398
288.13
23
2704
299.40

Figure 7B

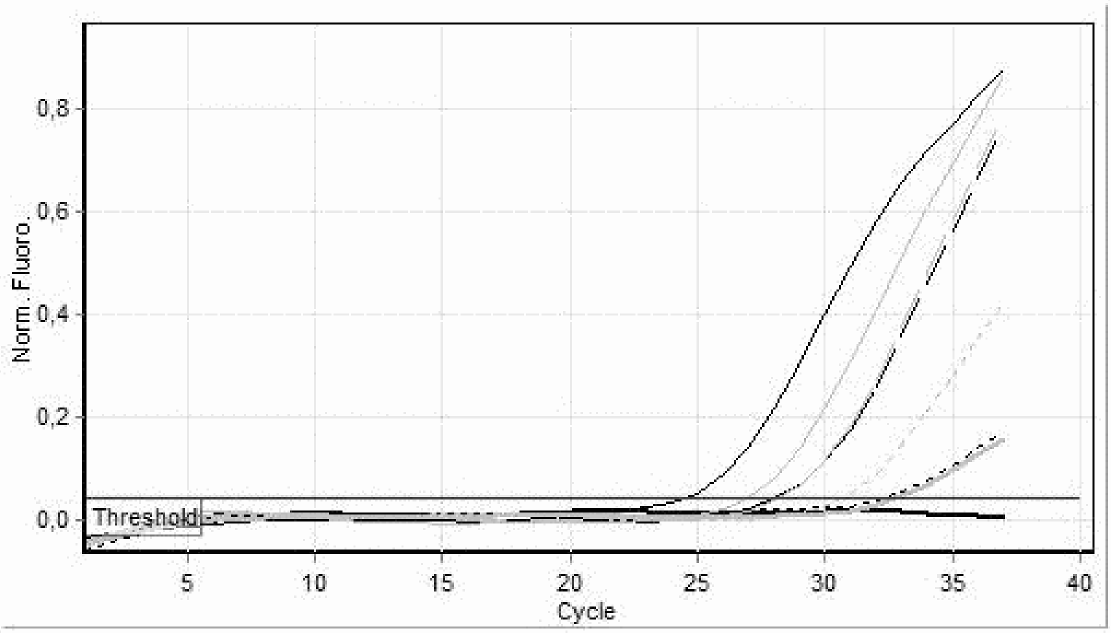

No.	Color	Pattern	Primers	Time (min)	Ct
1		Solid	CSFFW2416HSJ / CSFRV2216HS	0	24,53
2		Dashed	CSFFW2416HSJ / CSFRV2216HS	10	28,07
3		Dotted	CSFFW2416HSJ / CSFRV2216HS	30	32,39
4		Thin	CSFFW2416HSJ / CSFRV2216HS	NTC	
5		Solid	CSFFW200 / CSFRV60J	0	26,97
6		Dashed	CSFFW200 / CSFRV60J	10	28,23
7		Dotted	CSFFW200 / CSFRV60J	30	30,71
8		Thin	CSFFW200 / CSFRV60J	NTC	32,81

Figure 8

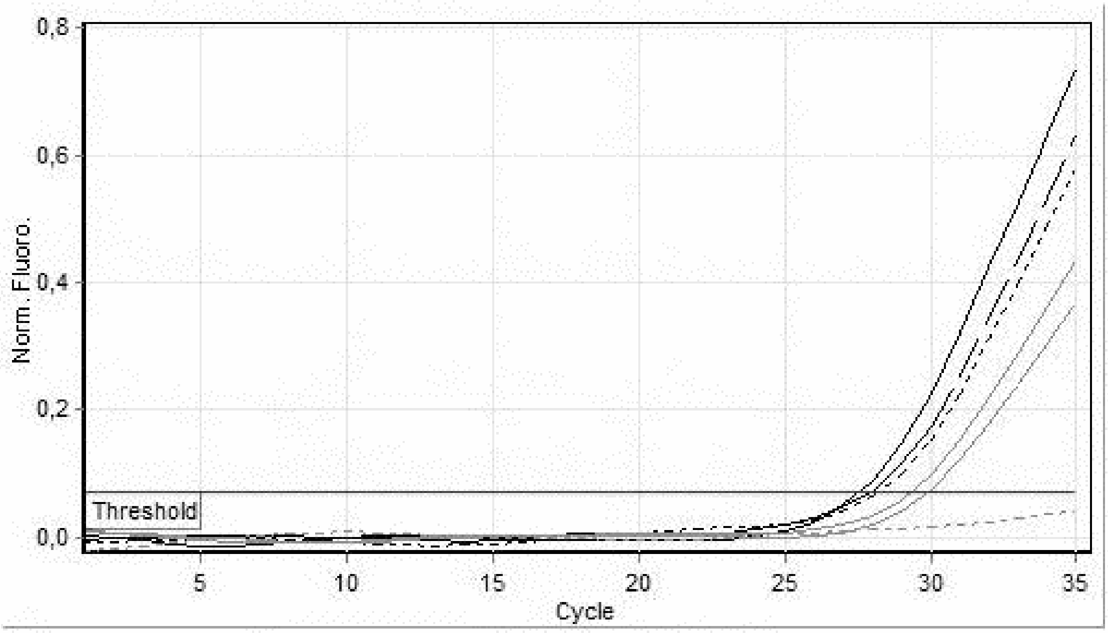

No.	Color	Pattern	Primers	Time (min)	Ct
1		Solid	CSFFW200 / CSFRV60J	0	27,59
2		Dashed	CSFFW200 / CSFRV60J	10	27,96
3		Dotted	CSFFW200 / CSFRV60J	30	28,27
4		Solid	CSFFW200 / CSFRV60J	45	29,37
5		Dashed	CSFFW200 / CSFRV60J	60	29,94
6		Dotted	CSFFW200 / CSFRV60J	NTC	

Figure 9A

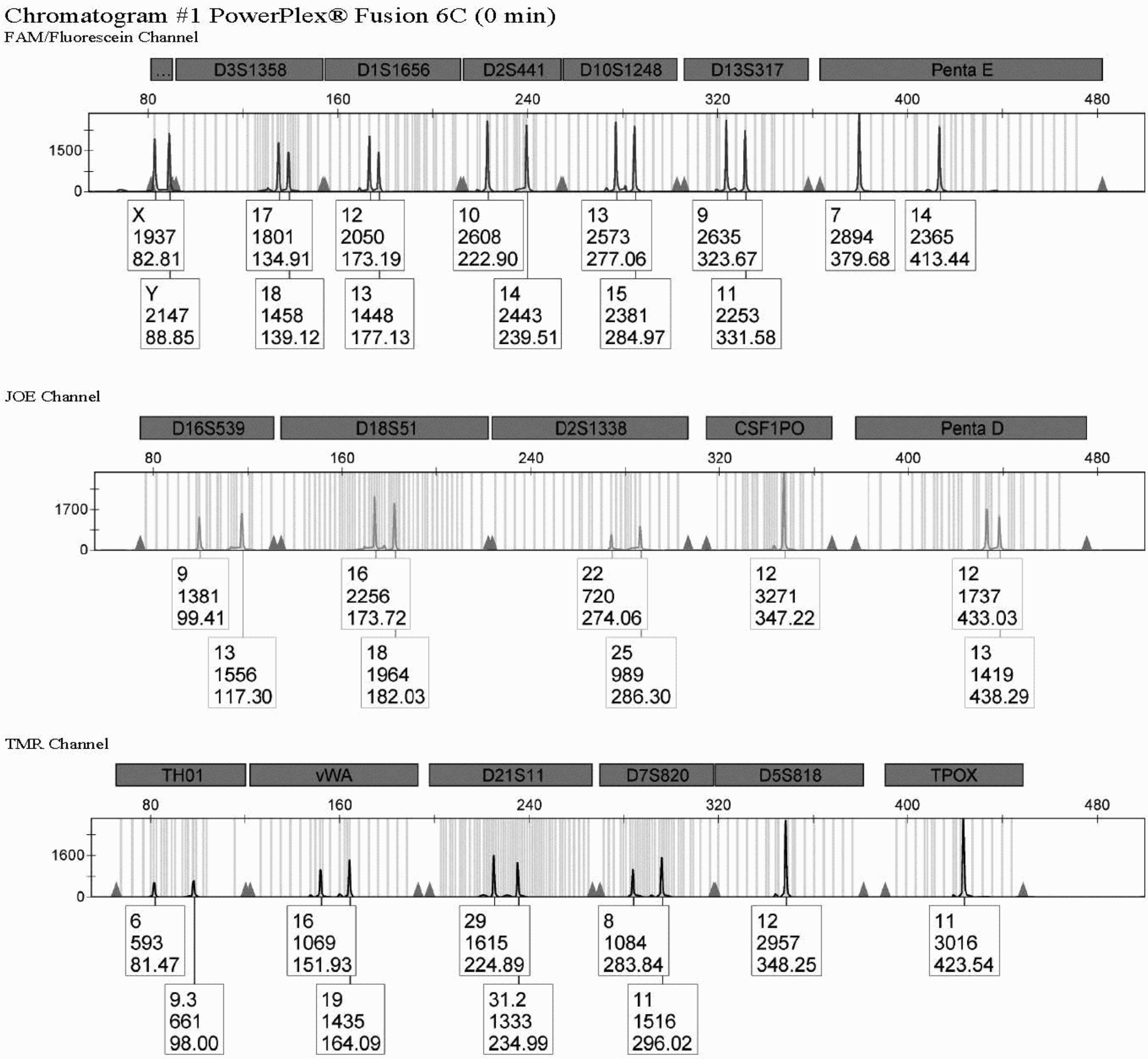

Figure 9B (continued)

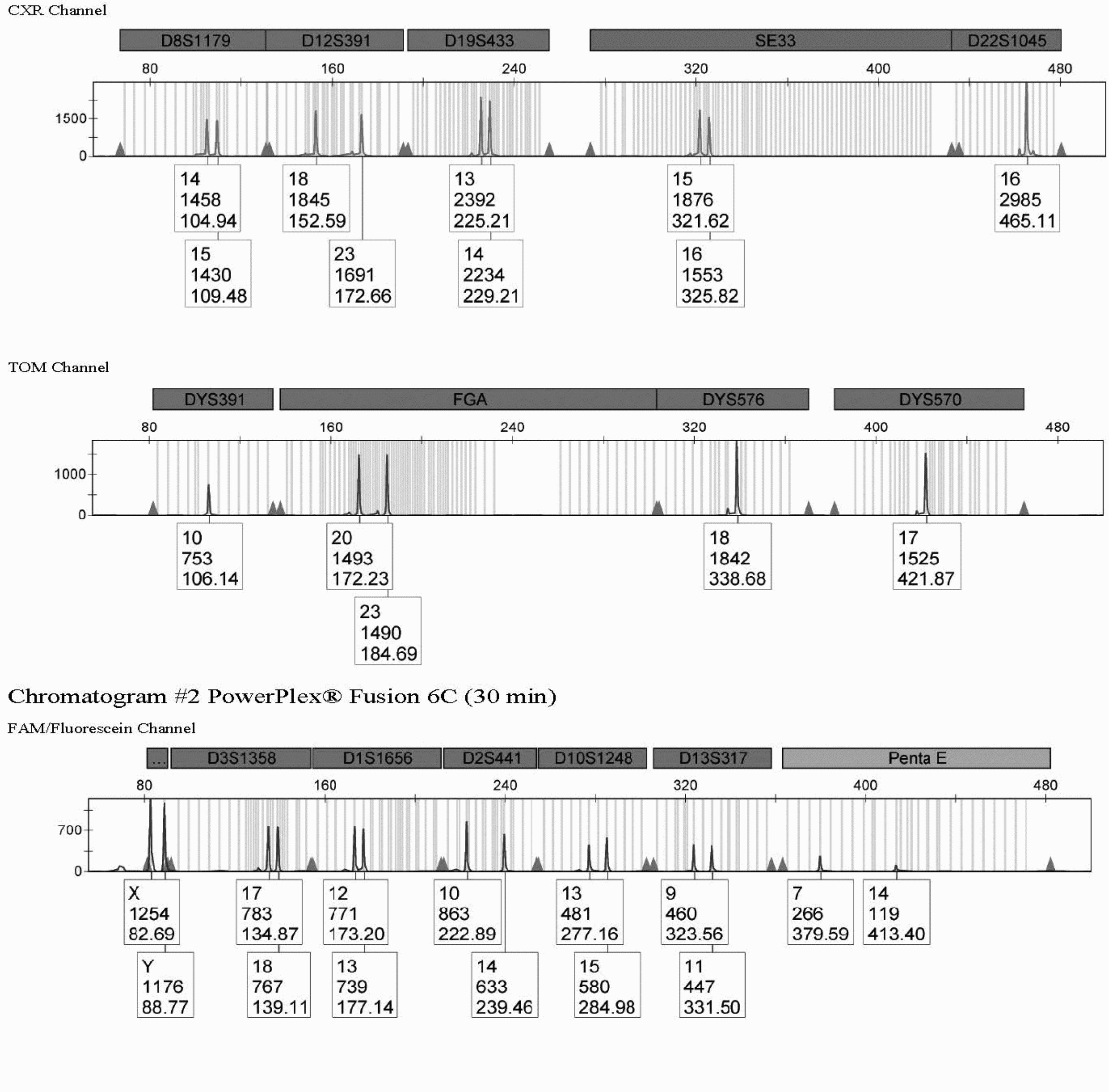

Figure 9B (continued)

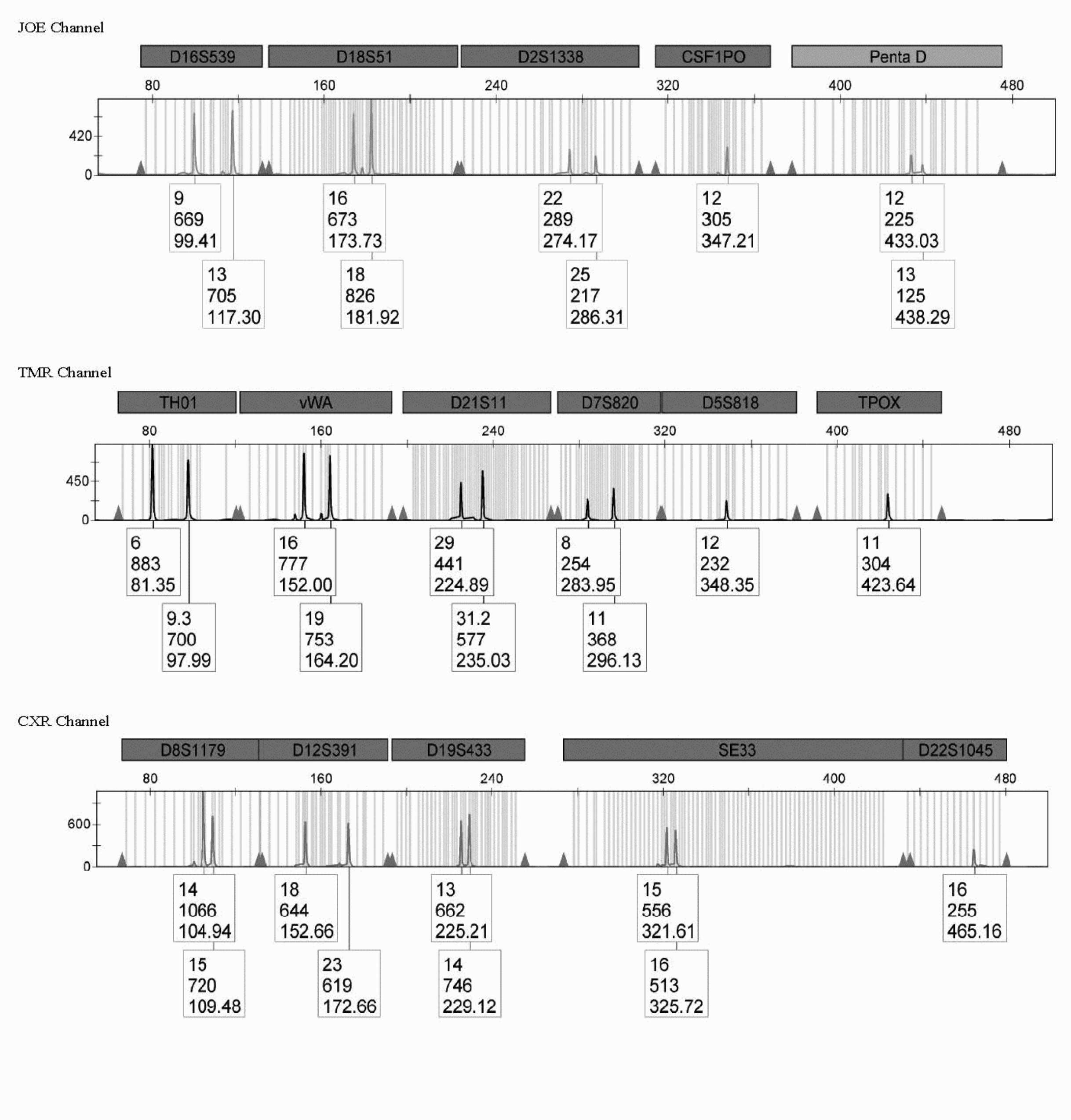

Figure 9B (continued)

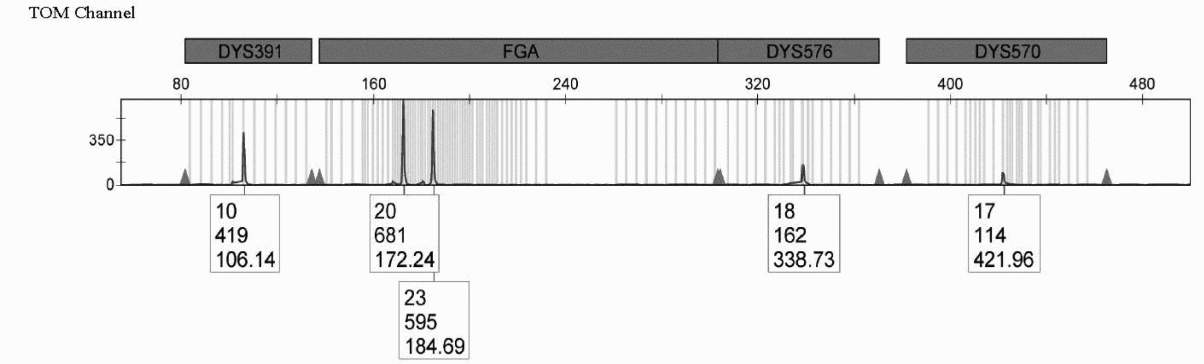

Chromatogram #3 PowerPlex® Fusion 6C (60 min)

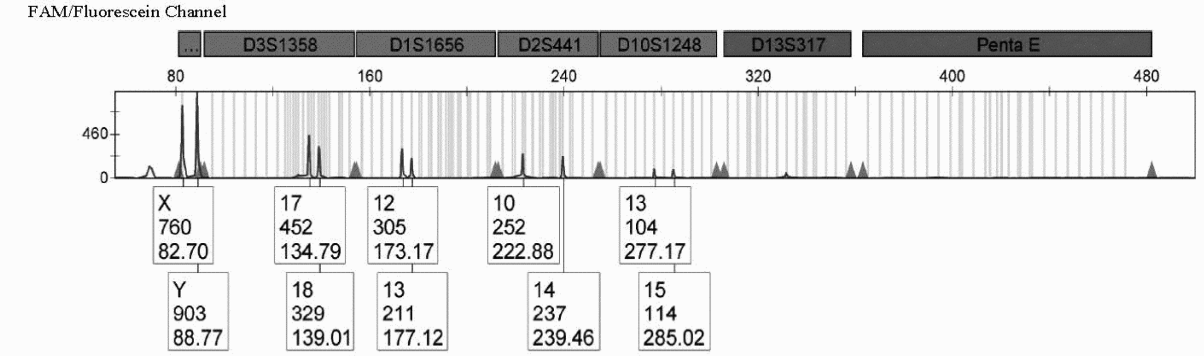

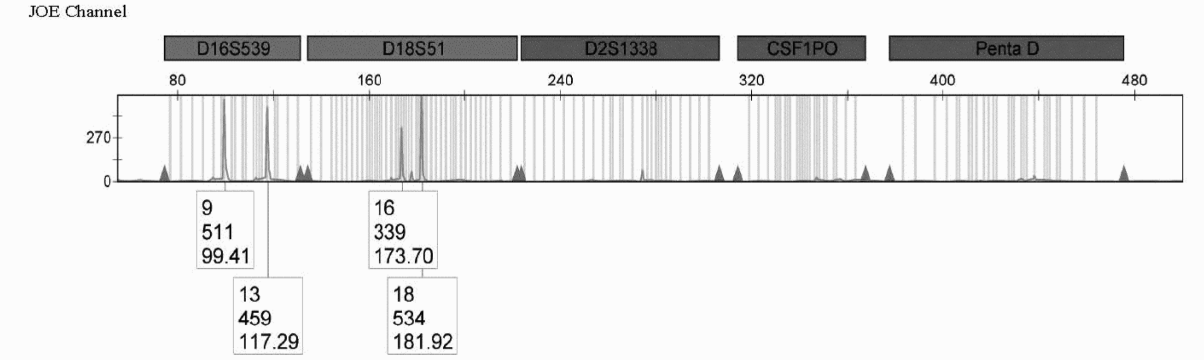

Figure 9B (continued)

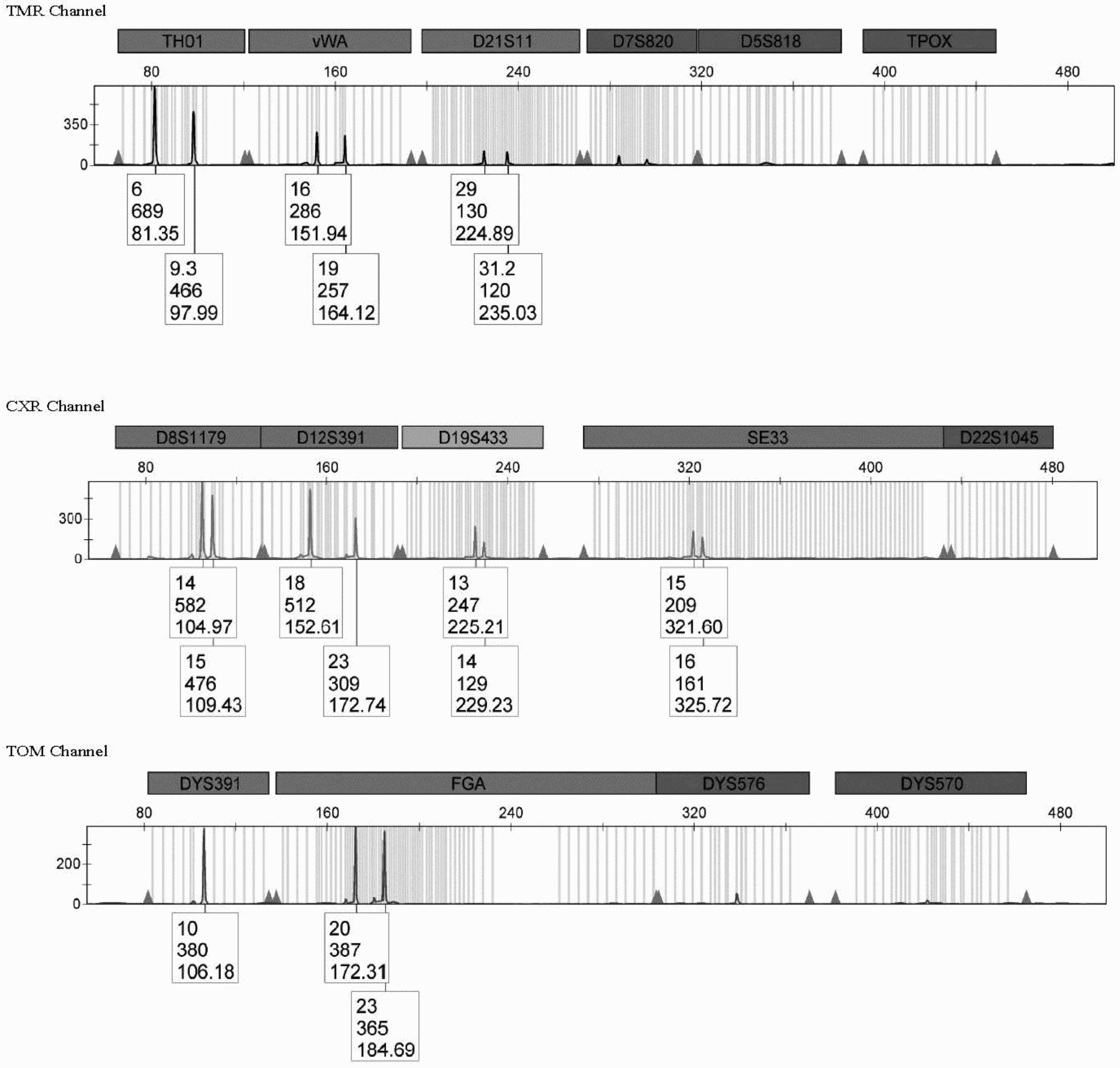

Figure 9B (continued)

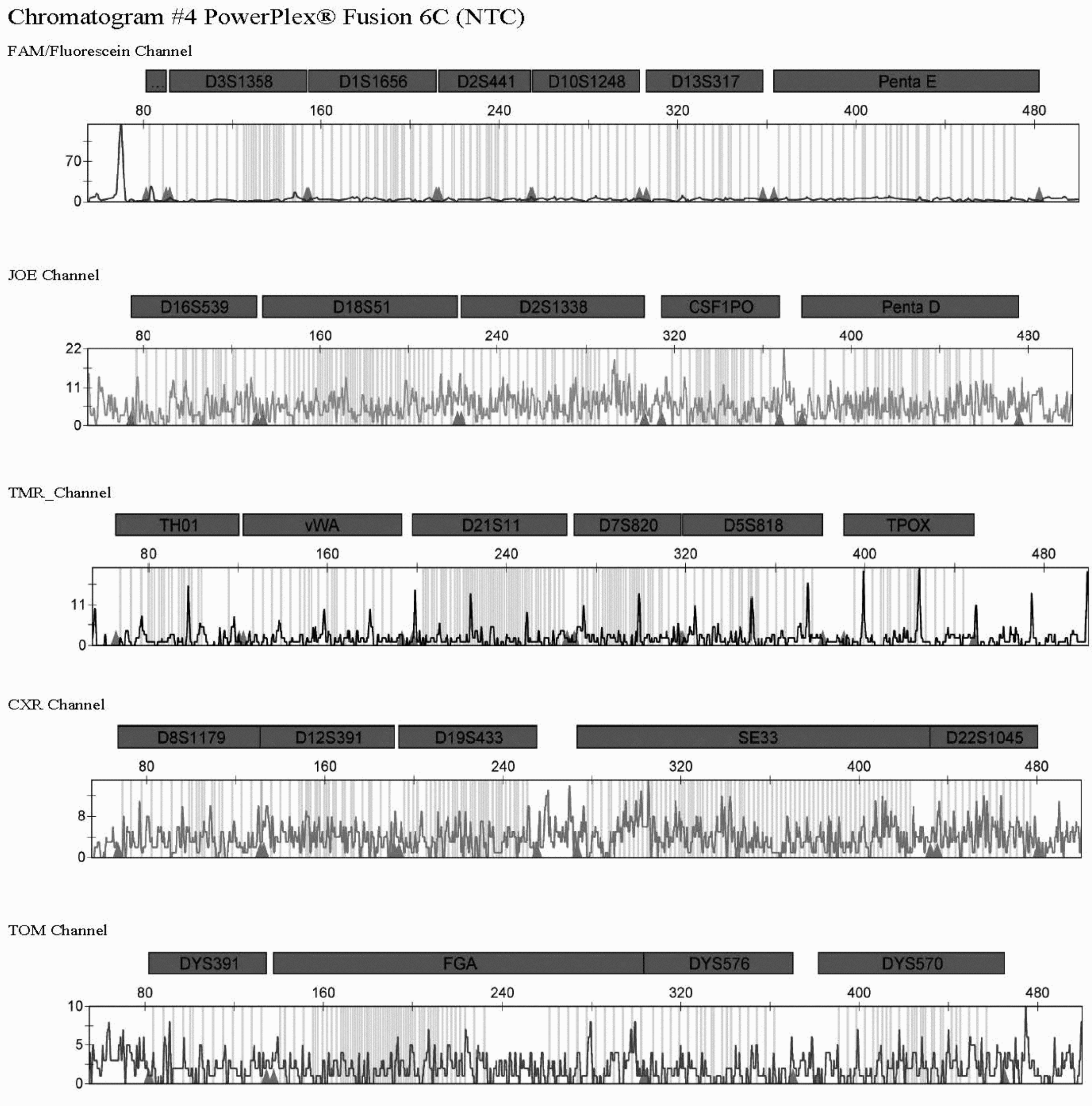

Figure 9B (continued)

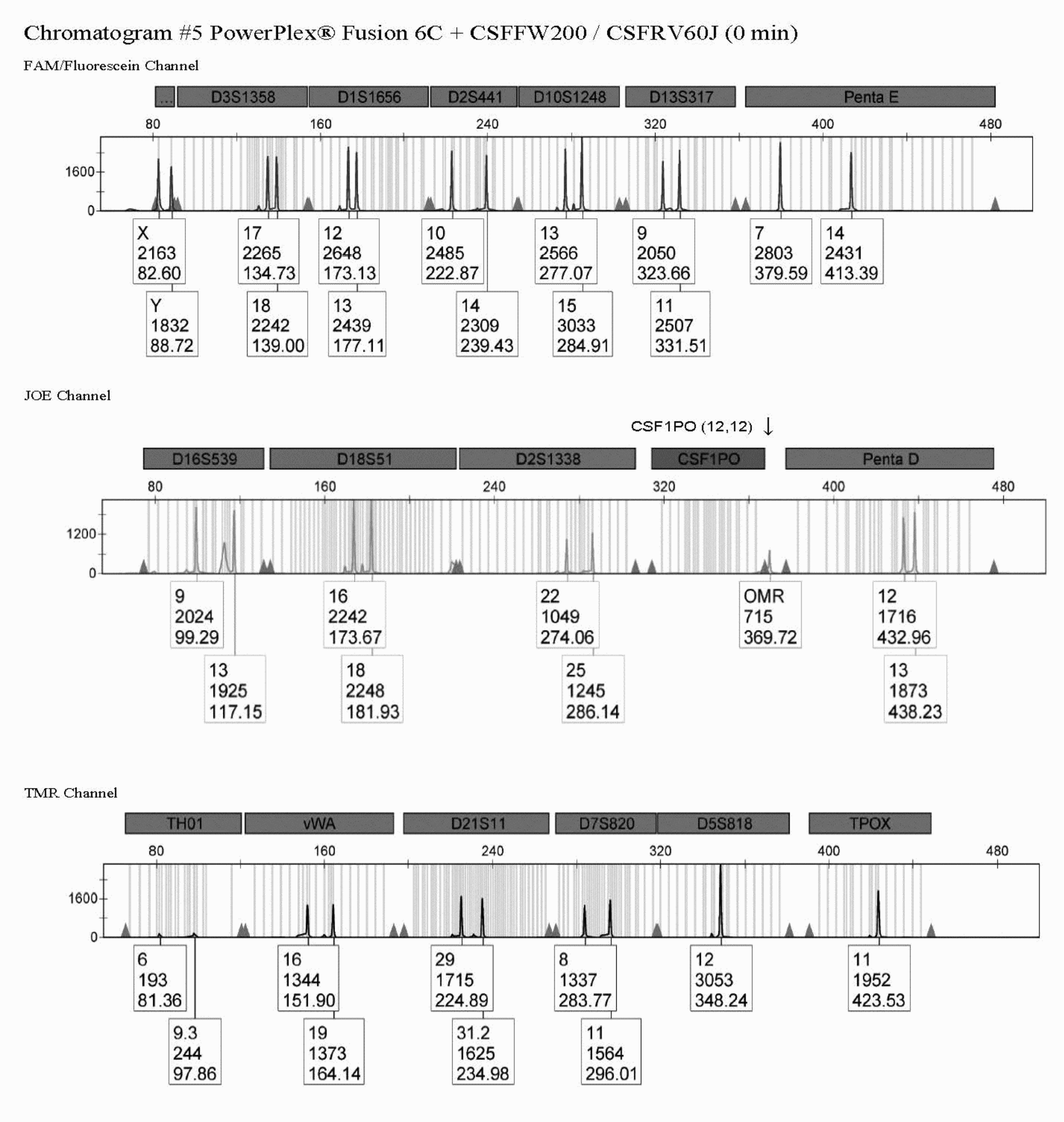

Figure 9B (continued)

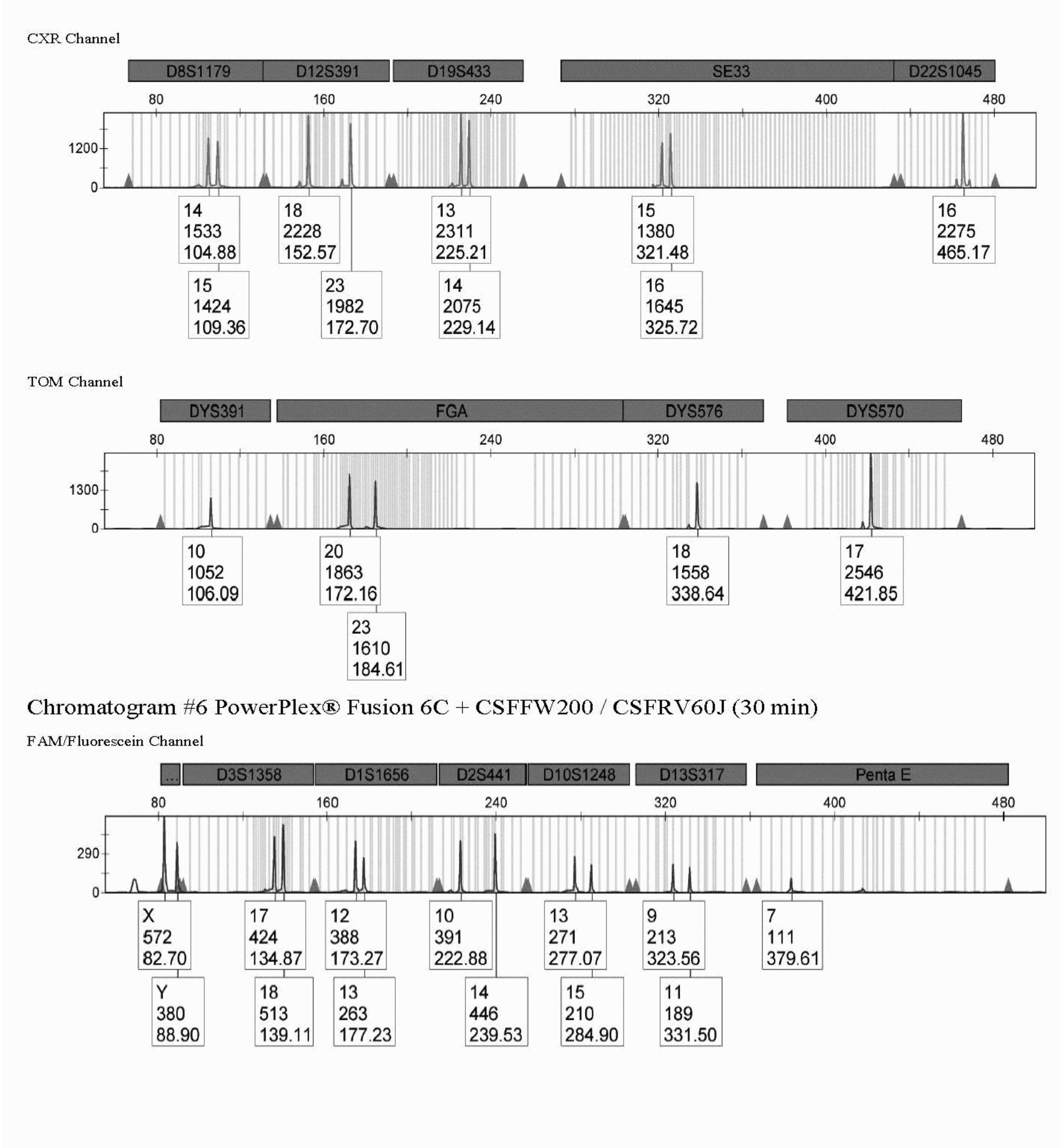

Figure 9B (continued)

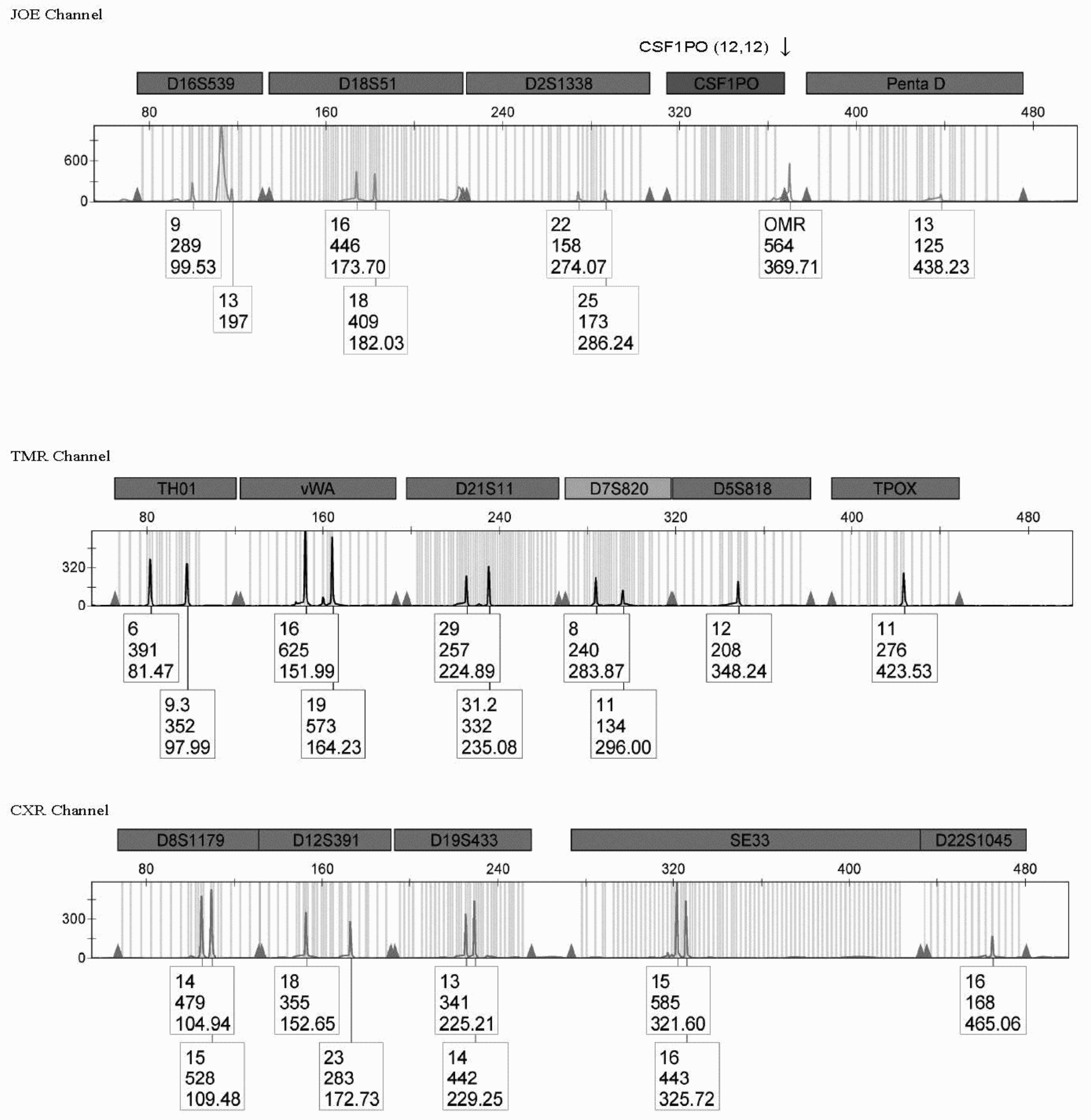

Figure 9B (continued)

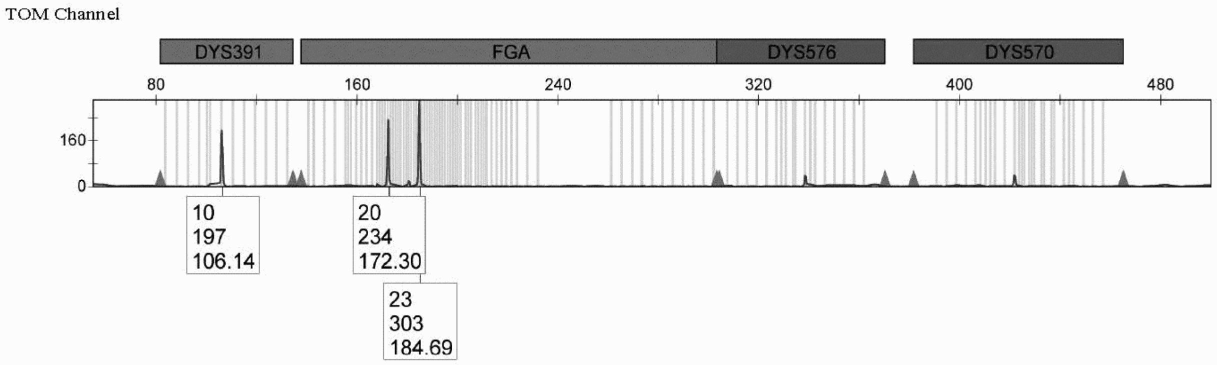

Chromatogram #7 PowerPlex® Fusion 6C + CSFFW200 / CSFRV60J (60 min)

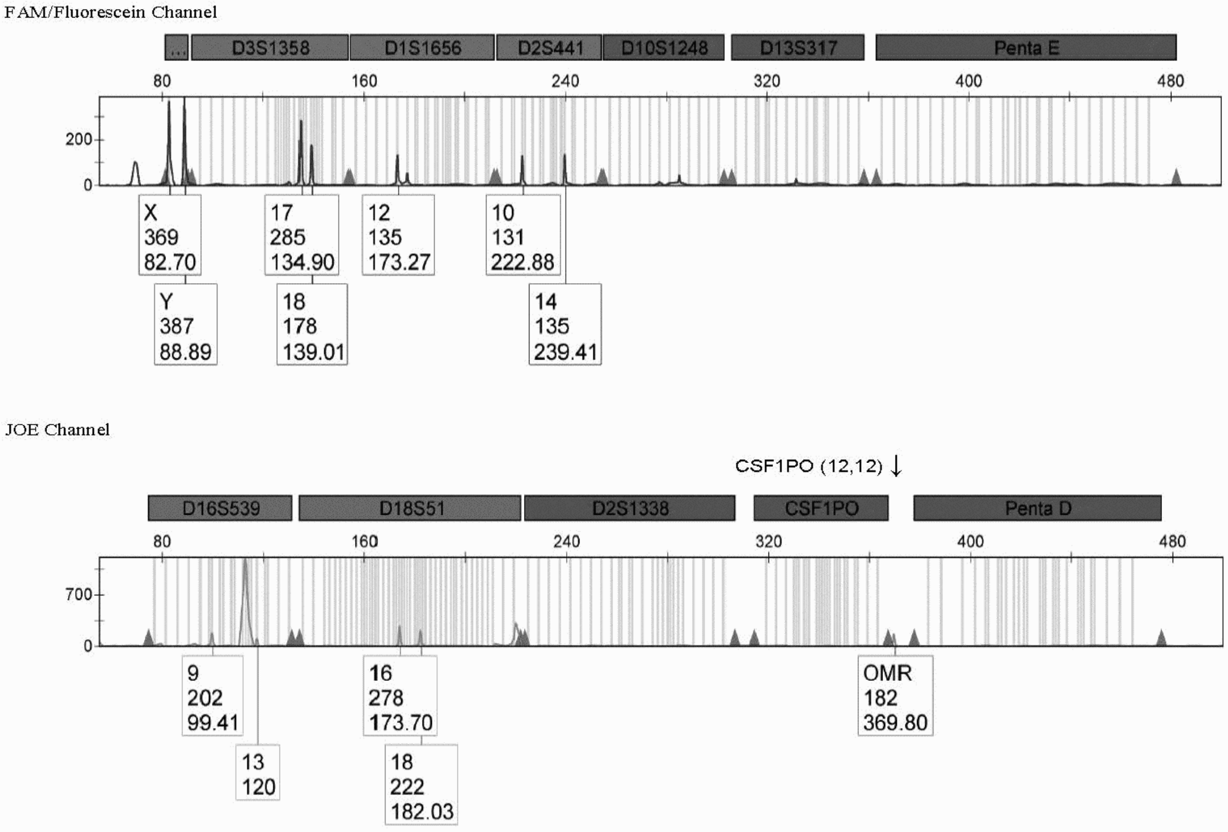

Figure 9B (continued)

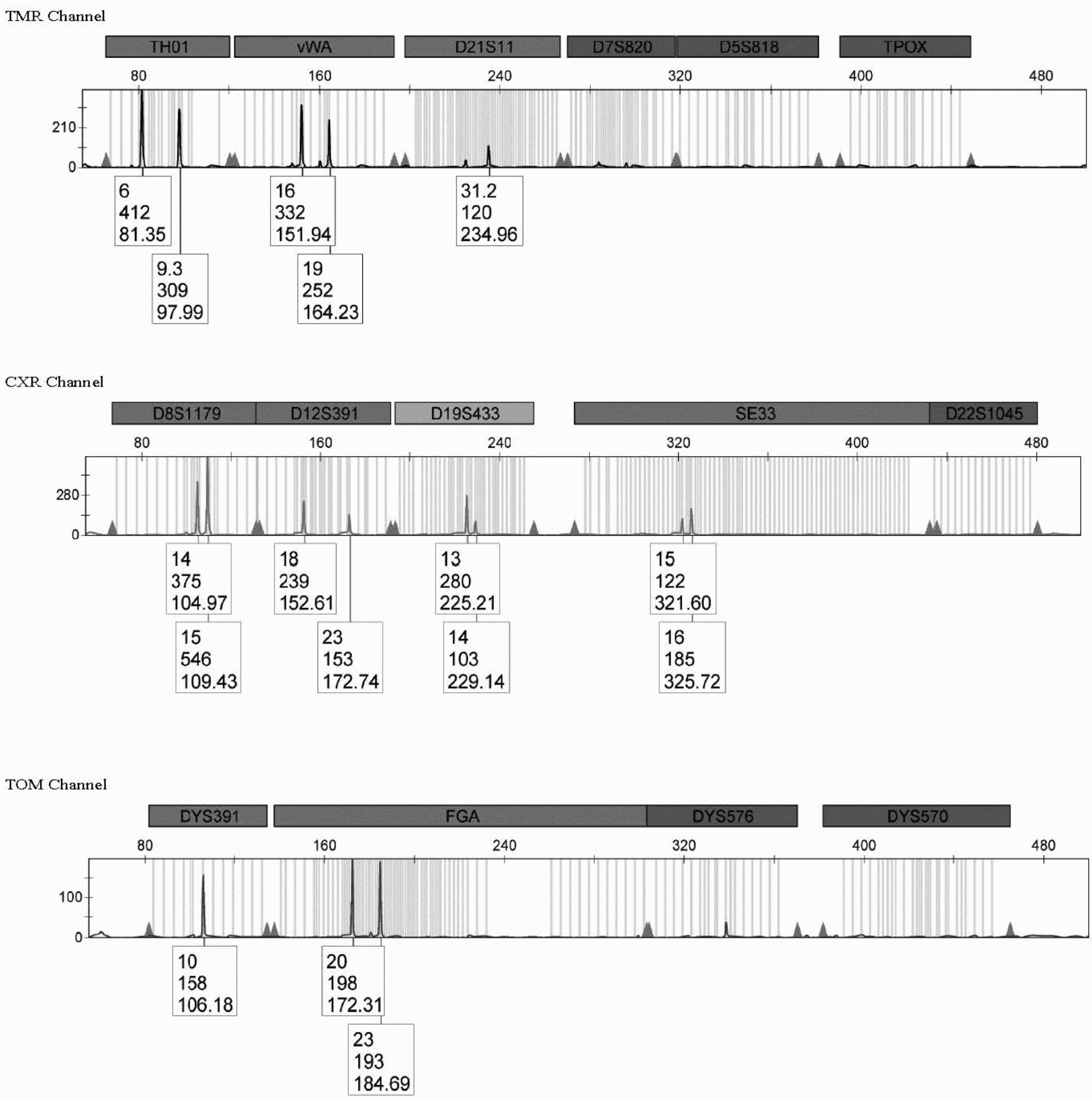

Figure 9B (continued)

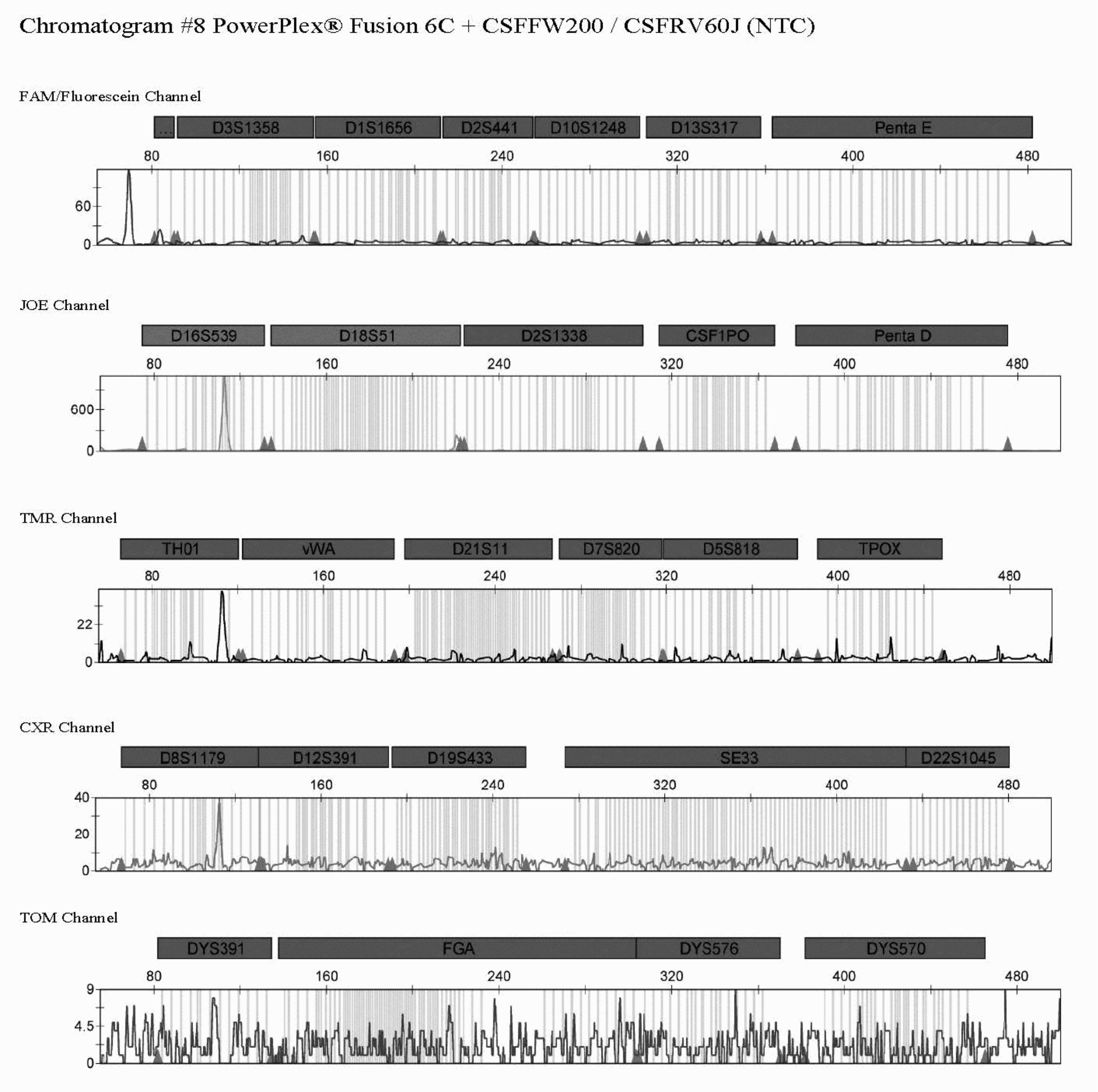
Chromatogram #8 PowerPlex® Fusion 6C + CSFFW200 / CSFRV60J (NTC)
FAM/Fluorescein Channel
D3S1358
D1S1656
D2S441
D10S1248
D13S317
Penta E
JOE Channel
D16S539
D18S51
D2S1338
CSF1PO
Penta D
TMR Channel
TH01
vWA
D21S11
D7S820
D5S818
TPOX
CXR Channel
D8S1179
D12S391
D19S433
SE33
D22S1045
TOM Channel
DYS391
FGA
DYS576
DYS570
80
160
240
320
400
480

Figure 9B

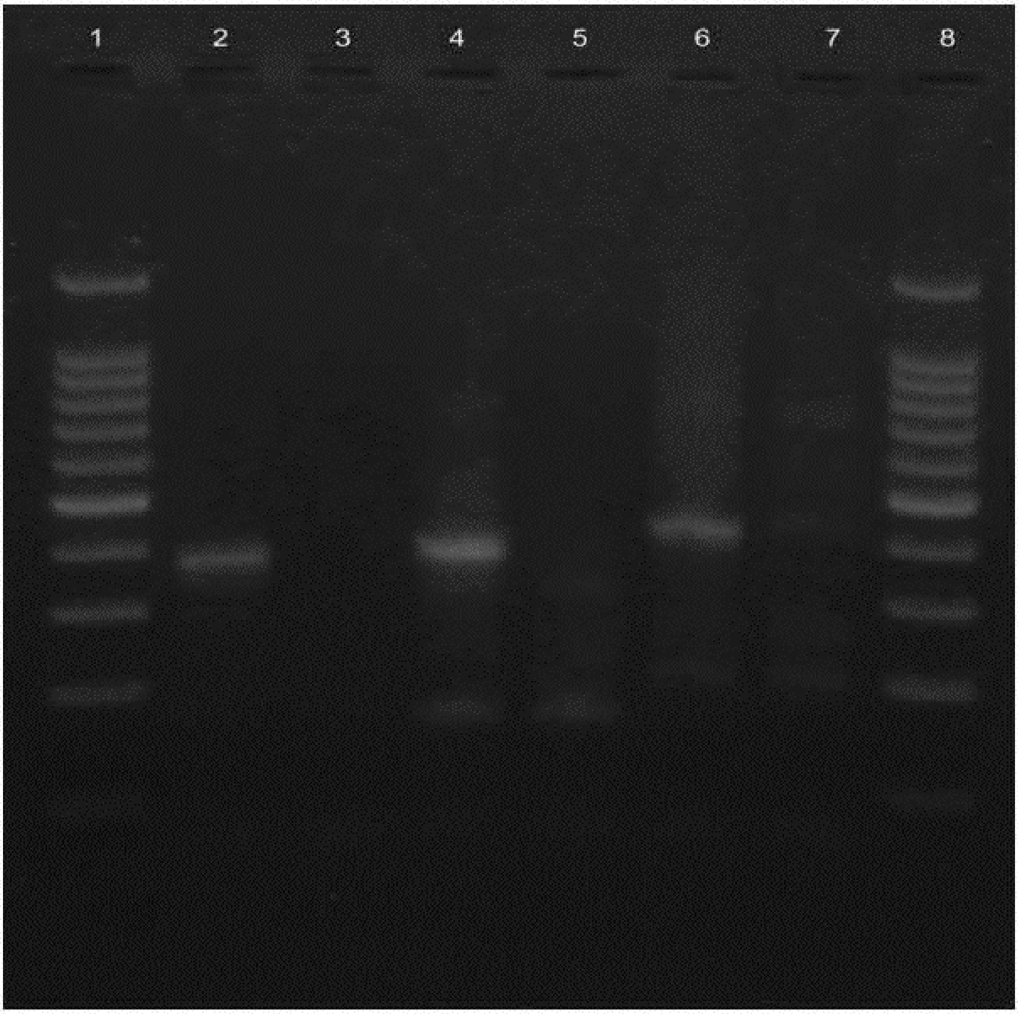

Figure 10A

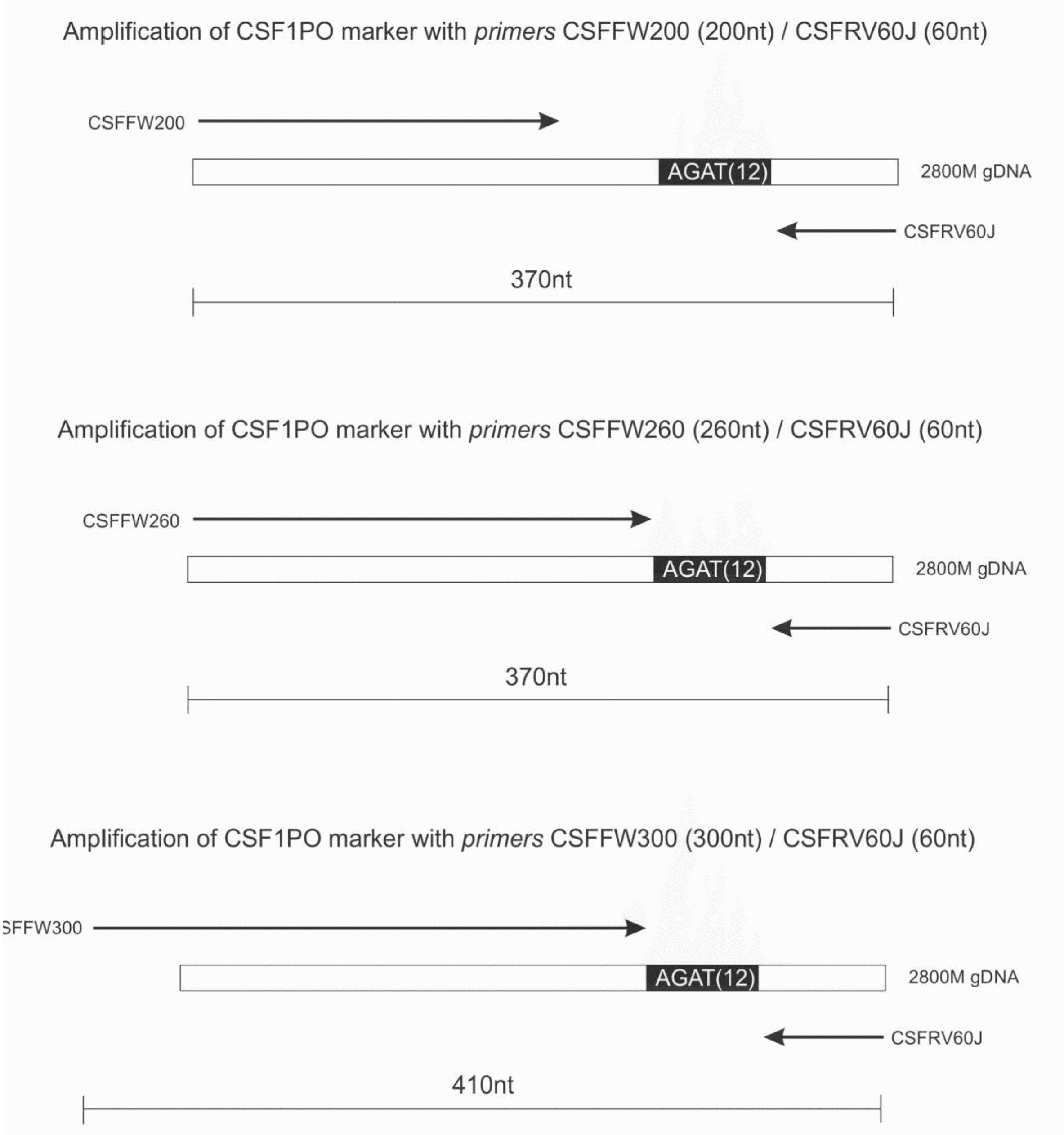
Amplification of CSF1PO marker with *primers* CSFFW200 (200nt) / CSFRV60J (60nt)
CSFFW200
AGAT(12)
2800M gDNA
CSFRV60J
370nt
Amplification of CSF1PO marker with *primers* CSFFW260 (260nt) / CSFRV60J (60nt)
CSFFW260
AGAT(12)
2800M gDNA
CSFRV60J
370nt
Amplification of CSF1PO marker with *primers* CSFFW300 (300nt) / CSFRV60J (60nt)
SFFW300
AGAT(12)
2800M gDNA
CSFRV60J
410nt

Figure 10B
